# Accelerated versus standard epirubicin followed by cyclophosphamide, methotrexate, and fluorouracil or capecitabine as adjuvant therapy for breast cancer in the randomised UK TACT2 trial (CRUK/05/19): a multicentre, phase 3, open-label, randomised, controlled trial

**DOI:** 10.1016/S1470-2045(17)30404-7

**Published:** 2017-07

**Authors:** David Cameron, James P Morden, Peter Canney, Galina Velikova, Robert Coleman, John Bartlett, Rajiv Agrawal, Jane Banerji, Gianfilippo Bertelli, David Bloomfield, A Murray Brunt, Helena Earl, Paul Ellis, Claire Gaunt, Alexa Gillman, Nicholas Hearfield, Robert Laing, Nicholas Murray, Niki Couper, Robert C Stein, Mark Verrill, Andrew Wardley, Peter Barrett-Lee, Judith M Bliss, TACT2 Investigators

**Affiliations:** a Cancer Research UK Edinburgh Centre, MRC Institute of Genetics and Molecular Medicine, University of Edinburgh, Edinburgh, UK; b ICR-CTSU, Division of Clinical Studies, Institute of Cancer Research, London, UK; c Department of Oncology, Beatson Hospital, Glasgow, UK; d St James' Institute of Oncology, University of Leeds, Leeds, UK; e Clinical Trials Research Unit (CTRU), University of Leeds, Leeds, UK; f Department of Oncology, Weston Park Hospital, Sheffield, UK; g Department of Diagnostic Development, Ontario Institute for Cancer Research, Toronto, ON, Canada; h Department of Oncology, Shrewsbury & Telford Hospitals NHS Trust, Shrewsbury, UK; i Department of Oncology, Singleton Hospital, Swansea, UK; j Department of Oncology, Royal Sussex County Hospital, Brighton, UK; k Department of Oncology, Royal Stoke University Hospital, Stoke-on-Trent, UK; l Department of Oncology, Addenbrooke's Hospital, Cambridge, UK; m Department of Oncology, Guy's and St Thomas' NHS Foundation Trust, London, UK; n Cancer Research UK Clinical Trials Unit, University of Birmingham, UK; o Department of Oncology, Royal Surrey County Hospital, Guildford, UK; p Department of Oncology, Southampton General Hospital, Southampton, UK; q Cancer Clinical Trials Unit Scotland (CaCTUS), Glasgow, UK; r Department of Oncology, NIHR University College London Hospitals Biomedical Research Centre, London, UK; s Department of Oncology, Northern Centre for Cancer Care, Freeman Hospital, Newcastle, UK; t Department of Oncology, Christie Hospital, Manchester, UK; u Department of Oncology, Velindre NHS Trust, Cardiff, UK

## Abstract

**Background:**

Adjuvant chemotherapy for early breast cancer has improved outcomes but causes toxicity. The UK TACT2 trial used a 2×2 factorial design to test two hypotheses: whether use of accelerated epirubicin would improve time to tumour recurrence (TTR); and whether use of oral capecitabine instead of cyclophosphamide would be non-inferior in terms of patients' outcomes and would improve toxicity, quality of life, or both.

**Methods:**

In this multicentre, phase 3, randomised, controlled trial, we enrolled patients aged 18 years or older from 129 UK centres who had histologically confirmed node-positive or high-risk node-negative operable breast cancer, had undergone complete excision, and were due to receive adjuvant chemotherapy. Patients were randomly assigned to receive four cycles of 100 mg/m^2^ epirubicin either every 3 weeks (standard epirubicin) or every 2 weeks with 6 mg pegfilgrastim on day 2 of each cycle (accelerated epirubicin), followed by four 4-week cycles of either classic cyclophosphamide, methotrexate, and fluorouracil (CMF; 600 mg/m^2^ cyclophosphamide intravenously on days 1 and 8 or 100 mg/m^2^ orally on days 1–14; 40 mg/m^2^ methotrexate intravenously on days 1 and 8; and 600 mg/m^2^ fluorouracil intravenously on days 1 and 8 of each cycle) or four 3-week cycles of 2500 mg/m^2^ capecitabine (1250 mg/m^2^ given twice daily on days 1–14 of each cycle). The randomisation schedule was computer generated in random permuted blocks, stratified by centre, number of nodes involved (none vs one to three vs four or more), age (≤50 years vs >50 years), and planned endocrine treatment (yes vs no). The primary endpoint was TTR, defined as time from randomisation to first invasive relapse or breast cancer death, with intention-to-treat analysis of standard versus accelerated epirubicin and per-protocol analysis of CMF versus capecitabine. This trial is registered with ISRCTN, number 68068041, and with ClinicalTrials.gov, number NCT00301925.

**Findings:**

From Dec 16, 2005, to Dec 5, 2008, 4391 patients (4371 women and 20 men) were recruited. At a median follow-up of 85·6 months (IQR 80·6–95·9) no significant difference was seen in the proportions of patients free from TTR events between the accelerated and standard epirubicin groups (overall hazard ratio [HR] 0·94, 95% CI 0·81–1·09; stratified p=0·42). At 5 years, 85·9% (95% CI 84·3–87·3) of patients receiving standard epirubicin and 87·1% (85·6–88·4) of those receiving accelerated epirubicin were free from TTR events. 4358 patients were included in the per-protocol analysis, and no difference was seen in the proportions of patients free from TTR events between the CMF and capecitabine groups (HR 0·98, 95% CI 0·85–1.14; stratified p=0·00092 for non-inferiority). Compared with baseline, significantly more patients taking CMF than those taking capecitabine had clinically relevant worsening of quality of life at end of treatment (255 [58%] of 441 vs 235 [50%] of 475; p=0·011) and at 12 months (114 [34%] of 334 vs 89 [22%] of 401; p<0·001 at 12 months) and had worse quality of life over time (p<0·0001). Detailed toxicity and quality-of-life data were collected from 2115 (48%) of treated patients. The most common grade 3 or higher adverse events in cycles 1–4 were neutropenia (175 [16%]) and fatigue (56 [5%]) of the 1070 patients treated with standard epirubicin, and fatigue (63 [6%]) and infection (34 [3%]) of the 1045 patients treated with accelerated epirubicin. In cycles 5–8, the most common grade 3 or higher adverse events were neutropenia (321 [31%]) and fatigue (109 [11%]) in the patients treated with CMF, and hand-foot syndrome (129 [12%]) and diarrhoea (67 [6%]) in the 1044 patients treated with capcitabine.

**Interpretation:**

We found no benefit from increasing the dose density of the anthracycline component of chemotherapy. However, capecitabine could be used in place of CMF without significant loss of efficacy and with improved quality of life.

**Funding:**

Cancer Research UK, Amgen, Pfizer, and Roche.

**Research in context**

**Evidence before this study**

At the time this study was designed in 2004, a series of meta-analyses of individual patients' data had established that the use of adjuvant chemotherapy improved survival in those with early breast cancer. No optimum regimen had been established and, although the benefits of adding taxanes to the previously established standard anthracycline-based chemotherapy regimens had been shown, the absolute benefit for patients varied. Some evidence suggested that specific tumour types were not associated with much benefit. Findings from some studies indicated that shortening the interval between chemotherapy cycles by using growth factors to hasten the recovery of peripheral neutrophil concentrations improved efficacy. This approach was called accelerated chemotherapy, and was becoming the standard of care in parts of the world, but the designs of the trials on which use was based did not make it clear whether the benefits were due to the accelerated regimen alone. Perhaps the most important study that had been reported when we designed TACT2 was the US CALGB 9741 study, in which the schedule of the paclitaxel component (for which growth factor support is not needed) was also changed, but the relative contribution of that component was unclear. This is important because the use of growth factors not only adds to the cost of chemotherapy but also increases the risk of toxic effects. Toxicity of adjuvant chemotherapy was, and remains, an important issue for patients, and in particular for one of the standard UK regimens, epirubicin followed by cyclophosphamide, methotrexate, and fluorouracil (CMF), it was observed in an analysis of two trials that all treatment-related deaths occurred while patients were taking CMF. We designed TACT2 to investigate whether use of accelerated epirubicin chemotherapy would improve time to tumour recurrence and whether using oral capecitabine instead of CMF would be non-inferior for efficacy but superior for toxicity, quality of life, or both.

**Added value of this study**

We found no benefit from accelerating the anthracycline component of a standard UK chemotherapy regimen. Furthermore, detailed analysis of the toxicity and tolerability of accelerated chemotherapy showed that accelerated epirubicin was not associated with fewer hospital admissions than standard epirubicin, as had been hoped, nor with better quality of life from the patients' perspective. Use of capecitabine instead of CMF after epirubicin was not associated with inferior disease outcomes, and was, as hypothesised, associated with a different and generally better tolerated toxicity profile. Importantly, this difference was confirmed by better patient-reported quality of life scores for women during capecitabine treatment than during CMF treatment. A notable effect was that fewer women receiving capecitabine became permanently menopausal than those taking CMF.

**Implications of all the available evidence**

Several studies have indicated benefits or otherwise with accelerated chemotherapy in early breast cancer, but the regimens and schedules assessed have differed. A meta-analysis will be important to determine circumstances in which clear benefits can be achieved. The large size of TACT2 means it will be an important contributor to such an analysis. Our observations on the lack of subjective benefit on quality of life with accelerated epirubicin will be important to form part of any discussion between patients and doctors about the possible benefits and risks of using an accelerated chemotherapy regimen. We found also that capecitabine can be used after epirubicin without loss of efficacy and with improved toxicity and quality of life outcomes compared with CMF. Furthermore, for premenopausal women concerned about retaining ovarian function, epirubicin followed by capecitabine seemed to reduce the risk of permanent chemotherapy-induced menopause.

## Introduction

Current outcomes for patients with early breast cancer reflect advances in diagnosis and therapy, including the standardisation of adjuvant systemic therapy.^1^ Chemotherapy is administered to many patients with early breast cancer, and the use of anthracyclines has improved efficacy^2^ compared with older non-anthracycline regimens. Although the addition of taxanes to anthracycline-based regimens has shown modest benefits, the growing recognition of breast cancer as a heterogeneous disease has prompted post-hoc analyses which suggest that benefits with different regimens, with or without taxanes, could be restricted to specific subgroups.^3^, ^4^, ^5^ From the early 2000s, when little advantage to patients was expected from the addition of taxanes, block-sequential epirubicin and cyclophosphamide, methotrexate, and fluorouracil (CMF) was the anthracycline regimen of choice in many UK hospitals.^6^ However, in an analysis of two similar trials involving 2391 women who received epirubicin followed by CMF or CMF alone, all 20 treatment-related deaths occurred during treatment with CMF (six among those receiving epirubicin followed by CMF and 14 among those receiving CMF alone).^6^ A less toxic but equally effective alternative to CMF was, therefore, needed. The oral chemotherapy agent capecitabine was equally efficacious to, and less toxic than, CMF in the metastatic setting,^7^ which made it an obvious choice for testing as adjuvant therapy in the TACT2 trial as a substitution for CMF after an anthracycline.

Further improvements in chemotherapy efficacy were sought by shortening the interval between cycles from the standard 21 days, a concept that was termed accelerated chemotherapy. The CALGB 9741 trial^3^ showed a significant advantage with doxorubicin and cyclophosphamide followed by paclitaxel every 2 weeks compared with the standard of every 3 weeks. A clear advantage has also been reported for paclitaxel given once per week.^3^, ^8^, ^9^ The relative contributions of the accelerated anthracycline regimen versus the use of paclitaxel to the advantage, however, were unclear.

We designed the TACT2 trial to investigate two questions using standard epirubicin followed by CMF: whether efficacy could be improved without increasing toxicity when the pure anthracycline chemotherapy component was accelerated; and whether capecitabine instead of CMF would improve tolerability without loss of efficacy. We used a pragmatic 2×2 factorial study design, which allowed TACT2 to run throughout the National Institute for Health Research (NIHR)-funded National Cancer Research Network in England and affiliated research networks supported by the Departments of Health in other parts of the UK permitting recruitment of breast cancer patients from both teaching and district general hospitals across the UK.

## Methods

### Study design and patients

TACT2 is a multicentre, phase 3, randomised, controlled trial with a 2×2 factorial design testing two hypotheses: first that accelerated epirubicin regimen will be superior in terms of time to tumour recurrence; and, second, that substituting CMF with the oral prodrug capecitabine will not worsen patients' outcomes and will offer advantages in terms of toxicity, quality of life, or both (appendix pp 42–99). Patients were recruited from 129 UK centres (appendix pp 1–3). Eligible patients were women or men aged 18 years or older with histologically confirmed invasive primary breast carcinoma (T0–3, N0–2, M0)^10^ who had undergone complete excision and were due to receive adjuvant chemotherapy. Patients needed to be fit to receive any of the trial chemotherapy regimens and to have adequate bone marrow, hepatic, and renal function. Patients had to be randomised and able to start assigned treatments within 8 weeks of surgery. Exclusion criteria (appendix p 10) included malignant disease in the previous 10 years, except ductal carcinoma in situ, basal-cell carcinoma, and cervical carcinoma in situ, locally advanced or distant disease, surgical margins involved at any tumour site in the final operative resection, and severe cardiac or renal disorders.

The study was approved by the Scotland Multi-Research Ethics Committee (MREC 04/MRE00/88) and local research and development offices. Patients provided written informed consent before enrolment. The Clinical Trials and Statistics Unit at The Institute of Cancer Research, London, UK (ICR-CTSU), had overall responsibility for trial coordination with three collaborating clinical trials units (Cancer Clinical Trials Unit, Scotland, Edinburgh, UK; Leeds Clinical Trials Research Unit, Leeds, UK; and Cancer Research UK Clinical Trials Unit, Birmingham, UK) that were responsible for randomisation and data management of patients within their geographical regions. Safety and efficacy data were reviewed regularly by an independent data monitoring committee. An independent trial steering committee provided trial oversight on behalf of the funders and sponsors. The trial management group was responsible for the day-to-day running of the trial. ICR-CTSU did all central statistical monitoring and the interim and final analyses.

### Randomisation and masking

The randomisation schedule was generated by computer at ICR-CTSU in permuted blocks with sizes of eight and 12. Patients were assigned in a 1:1:1:1 ratio to receive standard epirubicin followed by CMF, accelerated epirubicin followed by CMF, standard epirubicin followed by capecitabine, or accelerated epirubicin followed by capecitabine. Randomisation was performed via a telephone call to one of the four clinical trials units. Patients were stratified by centre, number of nodes involved (none *vs* one to three *vs* four or more), age (≤50 years *vs* >50 years), and planned endocrine treatment (yes *vs* no). TACT2 was open label because the different treatment schedules made a double-blind design impractical.

### Procedures

Patients assigned to the standard epirubicin group received four treatment cycles with 100 mg/m^2^ epirubicin delivered every 3 weeks, and those assigned to the accelerated epirubicin group received four cycles with 100 mg/m^2^ epirubicin delivered every 2 weeks plus 6 mg pegfilgrastim given on day 2 of each cycle. In the standard epirubicin groups, patients could receive pegfilgrastim as secondary prophylaxis against neutropenia. Patients subsequently received four 4-week cycles of CMF (600 mg/m^2^ cyclophosphamide intravenously on days 1 and 8 or 100 mg/m^2^ orally on days 1–14; 40 mg/m^2^ methotrexate intravenously on days 1 and 8; and 600 mg/m^2^ fluorouracil intravenously on days 1 and 8 of each cycle) or four 3-week cycles of 2500 mg/m^2^ capecitabine per day (1250 mg/m^2^ given twice daily on days 1–14 of each cycle).

Clinical, haematological, and biochemical assessments were done before the start of each cycle. Chemotherapy was administered only if neutrophil counts were 1·0×10^9^ cells per L or more and platelet counts were 100×10^9^ platelets per L or more. Supportive care was provided as per the local policy. The protocol stressed the need to maintain the interval between cycles, such that during standard epirubicin and CMF chemotherapy, any delay of longer than 7 days required a 20% dose reduction to minimise the risk of further dose delays (appendix pp 4–5).

After chemotherapy, standard radiotherapy, endocrine therapy, and 1 year of trastuzumab treatment were given according to local and national guidelines, and patients were permitted to enter further selected trials of adjuvant therapy provided these did not compromise the aims of TACT2 (appendix p 6).

Patients were followed up at 12, 18, and 24 months and then yearly for at least 10 years after randomisation, according to local practice for patients in trials of early breast cancer. Toxicity and clinical assessments were done at least every 6 months until the end of year 2 and annually thereafter. Imaging of the breasts (eg, by mammography or MRI) was done every 1 or 2 years for at least 10 years, in accordance with local practice. Patients in selected centres participated in a quality-of-life and toxicity substudy, for which data were obtained via questionnaires completed by patients and by collection of detailed information by clinicians. Due to logistical issues, collection of quality-of-life data was temporarily suspended part way through the trial and, therefore, lower numbers of patients are available for this analysis than that for acute toxicity. We used the European Organisation for Research and Treatment of Cancer (EORTC) QLQ-C30-BR23,^11^, ^12^ Hospital Anxiety and Depression Scale (HADS), Wu Cancer Fatigue Scale, Fatigue Symptoms Inventory (FSI), and the EQ-5D, and included TACT2-treatment-specific questions on toxicities during the treatment period (appendix pp 32–33). Adverse events were assessed by clinicians after each chemotherapy cycle and graded with the National Cancer Institute Common Toxicity Criteria for Adverse Events (version 3.0) and coded by use of the Medical Dictionary for Regulatory Activities (version 10), with central clinical review done by DC, PC, and PB-L when needed. Data on use of National Health Service (NHS) resources, including hospital admissions, were collected after each chemotherapy cycle and at each clinical follow-up visit for up to 5 years after treatment. Data on other adjuvant treatments were collected 12 months after randomisation. Data on ovarian function in women who were premenopausal at the start of chemotherapy were collected 18 months after randomisation.

### Outcomes

The primary endpoint was time to tumour recurrence (TTR), defined as the time from randomisation to first invasive relapse or breast cancer death. Patients who remained free from TTR events, including those who died from other causes in the absence of breast cancer relapse, were censored at their date of last follow-up or death. Deaths occurring after distant recurrence were classified as breast cancer deaths, irrespective of stated cause. Secondary endpoints were overall survival (time from randomisation to death from any cause), invasive-disease-free survival (time from randomisation to first invasive relapse, new second primary breast cancer, or death from any cause), time to distant tumour recurrence (time from randomisation to first invasive distant relapse, excluding ipsilateral supraclavicular fossa, or to breast cancer death), and tolerability of the regimens (assessed by treatment adherence and frequency and nature of acute adverse events).^11^ Questionnaires on quality of life and TACT2-treatment-specific toxicities were administered at baseline (after consent but before randomisation), at the end of standard or accelerated epirubicin, at the end of CMF or capecitabine, and at 12 and 24 months after the end of chemotherapy. The primary analysis of standard versus accelerated epirubicin was by intention to treat and the analysis of CMF versus capecitabine was per protocol.

### Statistical analysis

We assumed the 5-year proportion of patients free from TTR events would be 80% after standard epirubicin followed by CMF. To test whether accelerated epirubicin was superior to standard epirubicin, we calculated that 3876 patients would be needed to detect a 4% absolute improvement from 80% to 84% (hazard ratio [HR] 0·78) with 5% two-sided significance and 90% power. To test the second hypothesis that capecitabine would be non-inferior to CMF, we calculated that 4400 patients would be needed to provide 80% probability that the lower 90% CI for the difference between the regimens would exclude 3% if the groups were truly equivalent. Owing to a lower than expected number of TTR events, the independent data monitoring committee revised the hazard ratio threshold as the trial evolved. Therefore, a target accrual of 4400 patients was chosen, giving approximately 92% power for the comparison of standard versus accelerated epirubicin (appendix p 7).

For survival-related endpoints, we plotted Kaplan-Meier curves and compared treatment groups with the log-rank test. HRs and 95% CIs were calculated with Cox proportional hazards regression models, with HRs less than 1·0 taken to favour accelerated epirubicin or capecitabine. Unless stated otherwise, analyses were unadjusted and stratified by the companion randomisation. Analyses adjusted for randomisation stratification factors are also presented; all centres that assigned fewer than 25 patients to treatment are grouped into one category. Superiority analyses for all efficacy endpoints were done by intention to treat. The non-inferiority analysis and additional sensitivity analyses of the primary endpoint were done per protocol (ie, including all patients who received at least one cycle of allocated treatment). We did further analyses in a priori defined subgroups: companion randomisation regimen, oestrogen-receptor status, HER2 status, nodal status, age, tumour grade, tumour size, and vascular invasion. We also did exploratory subgroup analyses by menopausal status and molecular subtype (local oestrogen-receptor, progesterone-receptor, and HER2 status).

For the standard epirubicin versus accelerated epirubicin comparison, we calculated the actual fraction of the intended dose density for epirubicin, defined as the observed dose density divided by the protocol planned dose density for standard epirubicin, with the mean expected to be 1·5 times higher than that in patients who received standard epirubicin. For CMF and capecitabine, we calculated relative dose intensities (separately for each drug), defined as the observed dose intensity divided by protocol planned dose intensity. For both comparisons, for each chemotherapy cycle not received a relative dose intensity of zero was assumed.

The acute safety analysis population included all patients in the quality-of-life and toxicity substudy who received at least one cycle of allocated chemotherapy. The analysis of late toxicities compared all signs and symptoms reported 12 months or later after randomisation. The worst grades of adverse events were compared between treatment groups using the χ^2^ test for trend. The proportion of patients experiencing grade 3 or 4 adverse events in each treatment group was compared with Fisher's exact test. We took p values lower than 0·01 to be significant, which allowed some adjustment for multiple testing of safety endpoints.

The protocol specified two primary quality-of-life outcomes by which to compare CMF and capecitabine: overall quality of life, measured with the EORTC QLQ-C30 global health status quality-of-life scale (GHS/QOL), and HADS total score. Secondary prespecified measures of interest included EORTC QLQ-C30 subscales for physical function, role function, and fatigue; EORTC QLQ-C30-BR23 subscales for sexual function and systemic therapy side-effects; and the FSI, Wu Cancer Fatigue Scale, and TACT2-specific toxicity questions. Here we report only the findings from the primary quality-of-life assessment with EORTC QLQ-C30 GHS/QOL and the questions on TACT2-specific toxicity to supplement the adverse event data. The other findings will be reported separately. Cross-sectional analyses were done of differences in continuous EORTC QLQ-C30 GHS/QOL scores at the end of standard or accelerated epirubicin treatment, at the end of CMF or capecitabine treatment, and at 12 and 24 months after the end of the chemotherapy. For analyses of changes in EORTC QLQ-C30 GHS/QOL scores between baseline and these timepoints, subscales were taken to be continuous and were dichotomised by whether or not a patient's quality of life had worsened by ten or more points.^13^ Changes from baseline to each timepoint were compared between groups with ANCOVA with adjustment made for baseline scores. Generalised estimating equations models were used to analyse data longitudinally across all timepoints. In addition to randomised treatment, generalised estimating equations models included baseline score, time from baseline to follow-up questionnaire completion, component of quality-of-life study (before or after suspension), and age at randomisation. For patient-reported toxicity at each timepoint, the proportions of patients who reported suffering “quite a bit” or “very much” were compared between treatment groups with Fisher's exact test. Proportions of patients reporting each score were compared across treatment groups with a trend test.

Analyses presented here are based on a database frozen on Aug 25, 2015. All analyses were done with STATA version 13.0. This study is registered with the ISRCTN, number 68068041, and with ClinicalTrials.gov, number NCT00301925.

### Role of the funding sources

The funders of the study had no role in study design, data collection, data analysis, data interpretation, or writing of the report. The corresponding author had full access to all data in the study and had final responsibility for the decision to submit for publication.

## Results

**Figure 1:**
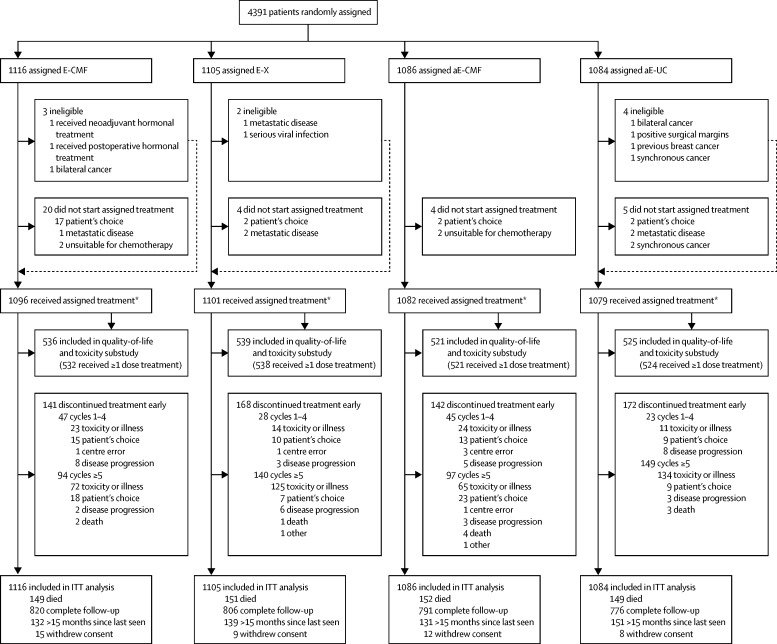
Trial profile E=standard epirubicin. aE=accelerated epirubicin. CMF=cyclophosphamide, methotrexate, and fluorouracil. X=capecitabine. ITT=intention-to-treat. *Per-protocol population.

Between Dec 16, 2005, to Dec 5, 2008, 4391 patients from 129 hospitals were enrolled, with 2221 patients allocated to receive standard epirubicin (1116 followed by CMF and 1105 followed by capecitabine) and 2170 to receive accelerated epirubicin (1086 followed by CMF and 1084 followed by capecitabine; figure 1). Baseline characteristics were well balanced across treatment groups (table 1). Of 4391 patients, 2711 (62%) were postmenopausal women and 20 (<1%) were men, 2337 (53%) were node positive, 2520 (57%) had grade 3 disease, 3196 (73%) had oestrogen-receptor-positive and/or progesterone-receptor-positive tumours, and 831 (19%) had HER2-positive tumours based on local assessments.

**Table 1:** Baseline characteristics and details of adjuvant treatments

			**Standard epirubicin followed by CMF (n=1116)**	**Standard epirubicin followed by capecitabine (n=1105)**	**Accelerated epirubicin followed by CMF (n=1086)**	**Accelerated epirubicin followed by capecitabine (n=1084)**
Age (years)			51·7 (45·8–59·6)	51·3 (45·0–59·0)	51·9 (45·8–59·3)	51·9 (45·9–58·8)
Sex						
	Female		1107 (99%)	1101 (>99 %)	1082 (>99%)	1081 (>99%)
	Male		9 (1%)	4 (<0·5%)	4 (<0·5%)	3 (<0·5%)
Menopausal status (female patients)						
	Premenopausal		428 (38%)	434 (39%)	396 (36%)	402 (37%)
	Postmenopausal		679 (61%)	667 (60%)	686 (63%)	679 (63%)
Local treatment*						
	Wide local excision		594 (53%)	578 (52%)	599 (55%)	621 (57%)
		With radiotherapy	573 (96%)	564 (98%)	584 (98%)	607 (98%)
	Mastectomy†		522 (47%)	527 (48%)	487 (45%)	462 (43%)
		With radiotherapy	316 (61%)	344 (65%)	296 (61%)	287 (62%)
Tumour size (cm)‡						
	≤2		473 (42%)	440 (40%)	451 (42%)	438 (40%)
	>2 to ≤5		584 (52%)	601 (54%)	572 (53%)	588 (54 %)
	>5		59 (5%)	63 (6%)	61 (6%)	58 (5%)
Tumour grade§						
	1		47 (4%)	39 (4%)	37 (3%)	52 (5%)
	2		415 (37%)	442 (40%)	418 (38%)	415 (38%)
	3		654 (59%)	622 (56%)	630 (58%)	614 (57%)
Nodes involved						
	0		533 (48%)	508 (46%)	508 (47%)	505 (47%)
	1–3		427 (38%)	470 (43%)	447 (41%)	436 (40%)
	≥ 4		156 (14%)	127 (11%)	131 (12%)	143 (13%)
Phenotype¶						
	Oestrogen-receptor positive, and/or progesterone-receptor positive, and HER2 negative (luminal)		686 (61%)	677 (61%)	643 (59%)	662 (61%)
	HER2 positive, oestrogen-receptor positive, and/or progesterone-receptor positive		123 (11%)	137 (12%)	141 (13%)	127 (12%)
	HER2 positive, oestrogen-receptor negative, and progesterone-receptor negative		73 (7%)	75 (7%)	77 (7%)	78 (7%)
	Triple negative‖		229 (21%)	211 (19%)	219 (20%)	208 (19%)
Endocrine therapy in patients with oestrogen-receptor and progesterone-receptor positive tumours**						
	Tamoxifen monotherapy		419 (51%)	429 (52%)	389 (49%)	399 (50%)
	Tamoxifen followed by aromatase inhibitor		120 (15%)	110 (13%)	103 (13%)	117 (15%)
	Aromatase inhibitor monotherapy		250 (31%)	258 (32%)	272 (35%)	260 (33%)
	Other		2 (<0·5%)	1 (<0·5%)	3 (<0·5%)	2 (<0·5%)
	None or unknown		23 (3%)	20 (2%)	21 (3%)	18 (2%)
Anti-HER2 therapy in patients with HER2-positive tumours††						
	Trastuzumab		185 (94%)	195 (92%)	198 (91%)	183 (89%)
	Lapatinib		1 (1%)	1 (<0·5%)	1 (<0·5%)	2 (1%)
	None or unknown		10 (5%)	16 (8%)	19 (9%)	20 (10%)

Data are median (IQR) or number (%). CMF=cyclophosphamide, methotrexate, and fluorouracil.

* One additional patient in the accelerated epirubicin followed by capecitabine group underwent axillary dissection only.

† Includes patients who had wide local excision and mastectomy.

‡ Tumour size not known for three patients (one in the standard epirubicin followed by capecitabine group and two in the accelerated epirubicin followed by CMF group).

§ Tumour grade not known for six patients (two in the standard epirubicin followed by capecitabine group, one in the accelerated epirubicin followed by CMF group, and three in the accelerated epirubicin followed by capecitabine group).

¶ Grouped according to locally assessed oestrogen-receptor, progesterone-receptor, and HER2 status. Progesterone-receptor status available for 3564 (81%) patients. Excludes 25 patients with HER2 status borderline or unknown (five in the standard epirubicin followed by CMF group, five in the standard epirubicin followed by capecitabine group, six in the accelerated epirubicin followed by CMF group, and nine in the accelerated epirubicin followed by capecitabine group).

‖ Includes 58 patients with oestrogen-receptor and HER2-negative tumours but unknown progesterone-receptor status (14 in the standard epirubicin followed by CMF group, 13 in the standard epirubicin followed by capecitabine group, 14 in the in the accelerated epirubicin followed by CMF group, and 17 in the accelerated epirubicin followed by capecitabine group).

** 814 standard epirubicin followed by CMF, 818 standard epirubicin followed by capecitabine, 788 epirubicin followed by CMF, and 796 accelerated epirubicin followed by capecitabine. Planned at 1 year after randomisation.

†† 196 standard epirubicin followed by CMF, 212 standard epirubicin followed by capecitabine, 218 epirubicin followed by CMF, and 205 accelerated epirubicin followed by capecitabine.

3735 (85%) of 4391 patients received all eight cycles of allocated treatment, with proportions being similar in the standard and accelerated epirubicin groups (appendix pp 8–9, figure 1). The mean actual fraction of the intended dose density for standard epirubicin over the first four cycles of treatment was 99·5% (IQR 95·0–100·0) and for accelerated epirubicin was 149·4% (141·9–150·3), giving a ratio between treatment groups of 1·51 (95% CI 1·49–1·52, appendix p 10). Of 2197 patients receiving at least one cycle of standard epirubicin, 124 (6%) received pegfilgrastim as secondary prophylaxis against neutropenia during epirubicin treatment. In the CMF and capecitabine groups, similar proportions of patients received all four cycles (appendix pp 8–9, figure 1). The median relative dose intensity across treatment cycles five to eight was 94·7% (IQR 80·4–99·7) for cyclophosphamide, 96·6% (82·4–99·9) for methotrexate, 96·2% (82·9–99·7) for fluorouracil, and 94·2% (75·9–99·4) for capecitabine. Use of other adjuvant therapies was similar across the two epirubicin groups (appendix pp 11–12).

**Figure 2:**
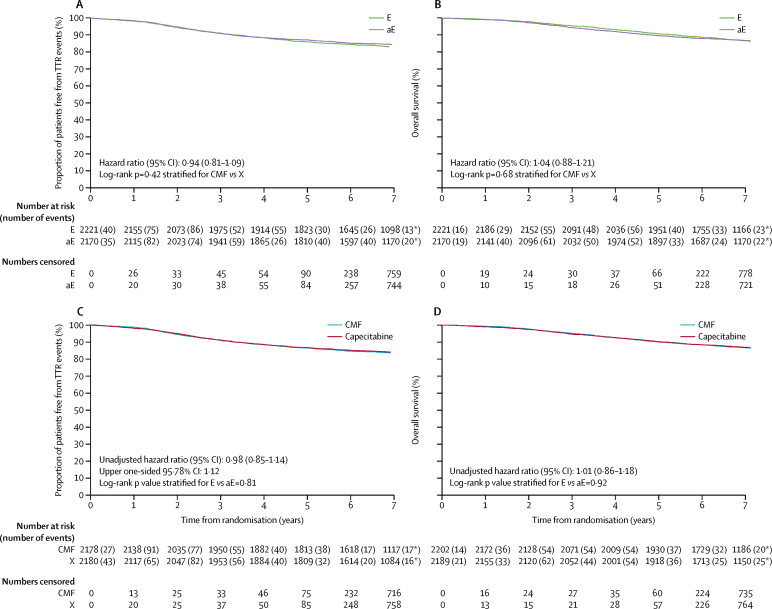
Kaplan-Meier plots for efficacy endpoints (A) Time to tumour recurrence in the intention-to-treat population for standard and accelerated epirubicin. (B) Overall survival in the intention-to-treat population for standard and accelerated epirubicin. (C) Time to tumour recurrence in the per-protocol population for CMF and capecitabine. (D) Overall survival in the intention-to-treat population for CMF and capecitabine. *Events that occurred after year 7. E=standard epirubicin. aE=accelerated epirubicin. CMF=cyclophosphamide, methotrexate, and fluorouracil. X=capecitabine. TTR=time to tumour recurrence.

Median follow-up in patients was 85·6 months (IQR 80·6–95·9), with 3193 (85%) of 3748 known to be alive without withdrawing consent followed up within the previous 15-month period. Median follow-up was 85·8 months (IQR 80·7–96·1) in the standard epirubicin and CMF group, 85·3 months (80·2–95·7) in the standard epirubicin and capecitabine group, 85·8 months (81·5–95·9) in the accelerated epirubicin and CMF group, and 85·6 months (79·8–95·8) in the accelerated epirubicin and capecitabine group. TTR events were reported in 724 (17%) of 4391 patients in the intention-to-treat population and in 716 (16%) of 4358 in the per-protocol population (table 2). There was no significant difference in TTR between the two epirubicin groups (377 [17%] of 2221 patients in the standard epirubicin group had TTR events, compared with 347 [16%] of 2170 patients in the accelerated epirubicin group, overall HR 0·94, 95% CI 0·81–1·09, stratified log-rank test p=0·42; figure 2). There was also no significant difference in TTR between the CMF and capecitabine groups (362 [17%] of 2178 patients in the CMF group had TTR events compared with 354 [16%] of 2180 patients in the capecitabine alone group, overall HR 0·98, upper 95·78% CI limit 1·12; 95% CI 0·85–1·14, p=0·00092 for non-inferiority and stratified log-rank test p=0·81 for superiority of capecitabine compared with CMF; figure 2). Results were similar for CMF and capecitabine in the intention-to-treat population (HR 0·99, upper 95·78% CI limit 1·12, 95% CI 0·86–1·15, p=0·0014 for non-inferiority and stratified log-rank test p=0·91 for superiority of capecitabine).

**Table 2:** Events contributing to analyses of time to tumour recurrence and numbers of distant relapses, second cancers, and deaths

			**Intention-to-treat analysis**		**Intention-to-treat analysis**		**Per-protocol analysis**	
			Standard epirubicin (n=2221)	Accelerated epirubicin (n=2170)	CMF (n=2202)	Capecitabine (n=2189)	CMF (n=2178)	Capecitabine (n=2180)
Number of patients with events contributing to time to tumour recurrence survival analysis*			377 (17%)	347 (16%)	365 (17%)	359 (16%)	362 (17%)	354 (16%)
	Distant recurrence		297 (13%)	274 (13%)	300 (14%)	271 (12%)	297 (14%)	267 (12%)
	Locoregional recurrence		80 (4%)	73 (3%)	65 (3%)	88 (4%)	65 (3%)	87 (4%)
New breast disease†			50 (2%)	48 (2%)	47 (2%)	51 (2%)	46 (2%)	51 (2%)
	Ipsilateral breast		4 (<0·*5*%)	14 (1%)	8 (<0·*5%*)	10 (1%)	8 (<0·*5%*)	10 (<0·5%)
	Contralateral breast		46 (2%)	34 (2%)	39 (2%)	41 (2%)	38 (2%)	41 (2%)
Non-breast second primary cancer			66 (3%)	68 (3%)	70 (3%)	64 (3%)	70 (3%)	64 (3%)
	Myeloid leukaemia		2 (<0·*5%*)	4 (<0·*5%*)	3 (<0·*5%*)	3 (<0·*5*%)	3 (<0·*5%*)	3 (<0·*5*%)
Deaths								
	All		300 (14%)	301 (14%)	301 (14%)	300 (14%)	299 (14%)	297 (14%)
	Breast cancer‡		253 (11%)	257 (12%)	257 (12%)	253 (12%)	255 (12%)	250 (11%)
	Other causes		47 (2%)	44 (2%)	44 (2%)	47 (2%)	44 (2%)	47 (2%)
		Infection related to chemotherapy	3 (<0·*5%*)	6 (<0·*5%*)	8 (<0·*5%*)	1 (<0·*5%*)	8 (<0·*5*%)	1 (<0·*5*%)
		Infection not related to chemotherapy	0	2 (<0·*5%*)	1 (<0·*5%*)	1 (<0·*5%*)	1 (<0·*5%*)	1 (<0·*5*%)
		Vascular	8 (<0·*5*%)	6 (<0·*5%*)	4 (<0·*5%*)	10 (<0·5%)	4 (<0·*5%*)	10 (<0·5%)
		Second cancer	28 (1%)	20 (1%)	21 (1%)	27 (1%)	21 (1%)	27 (1%)
		Other§	8 (<0·*5%*)	10 (<0·5%)	10 (1%)	8 (<0·*5%*)	10 (1%)	8 (<0·*5%*)

CMF=cyclophosphamide, methotrexate, and fluorouracil.

* First time to recurrence events are reported in this table. In 81 patients, distant recurrence was reported concurrently or within 30 days of locoregional recurrence, and these instances are reported as distant recurrence. Locoregional recurrence includes those to the ipsilateral supraclavicular fossa.

† Includes contralateral breast recurrence and new contralateral and ipsilateral breast second primary tumours.

‡ Includes six deaths from other causes after distant disease recurrence: infective endocarditis (n=1 in the standard epirubicin followed by CMF group), metabolic acidosis and sepsis from bronchopneumonia (n=1 in the standard epirubicin followed by CMF group), pancreatic cancer (n=1 standard epirubicin followed by capecitabine group and n=1 in the accelerated epirubicin followed by CMF group), a fall (n=1 in the accelerated epirubicin followed by capecitabine group), and ovarian cancer (n=1 in the accelerated epirubicin followed by capecitabine group).

§ Includes one death of unknown cause in the standard epirubicin followed by CMF group.

**Figure 3:**
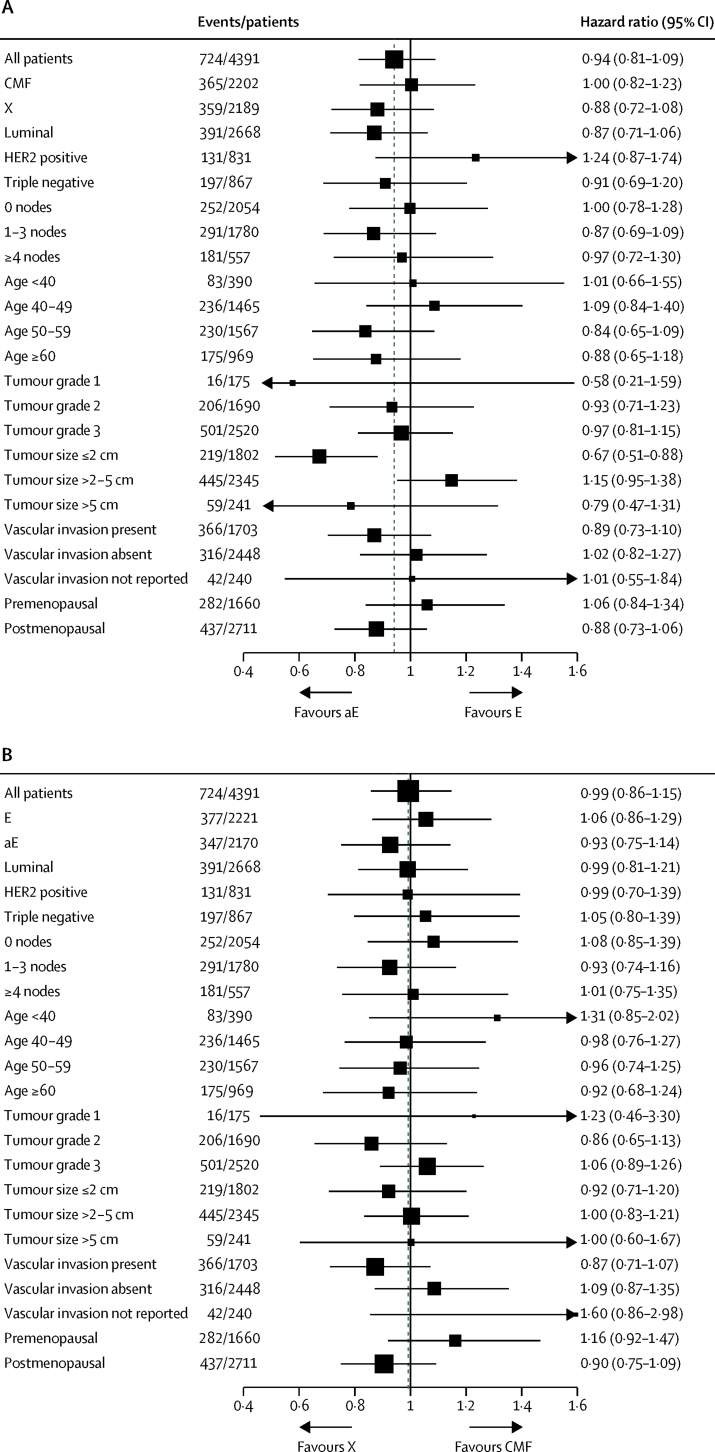
Forest plots of time to tumour recurrence, by characteristics of patients and tumours (A) Cycles one to four of treatment (E or aE). HER2 status was borderline or unknown for 25 patients (ten receiving E and 15 receiving aE). HER2-negative phenotype includes patients with borderline HER2 results and without fluorescence in situ hybridisation (n=10) and patients with no HER2 results (n=15 in luminal and triple negative subgroups). Triple negative phenotype group includes patients with oestrogen-receptor-negative tumours and unknown progesterone-receptor status (n=58). Tumour size was unknown for three patients (one receiving E and two receiving aE). Tumour grade unknown for six patients (two receiving E, and four receiving aE). (B) Cycles five to eight of treatment (CMF or X). HER2 status was borderline or unknown for 25 patients (11 receiving CMF and 14 receiving X). HER2-negative phenotype group includes patients with borderline HER2 results and without fluorescence in situ hybridisation (n=10) and patients with no HER2 result (n=15 in luminal and triple negative subgroups). Triple negative phenotype group includes patients with oestrogen-receptor-negative tumours and unknown progesterone-receptor status (n=58). Tumour size unknown for three patients (two receiving CMF and one receiving X). Tumour grade unknown for six patients (one receiving CMF and five receiving X). Analyses were done in the intention-to-treat population. E=standard epirubicin. aE=accelerated epirubicin. CMF=cyclophosphamide, methotrexate, and fluorouracil. X=capecitabine.

The proportion of patients free from TTR events at 3 years was 90·8% (95% CI 89·5–92·0) for the standard epirubicin group versus 91·1% (89·8–92·2) in the accelerated epirubicin group, and 91·0% (89·7–92·1) for the CMF group versus 91·2% (90·0–92·3) in the capecitabine group. The proportions of patients free from TTR events at 5 years were 85·9% (95% CI 84·3–87·3) for the standard epirubicin group versus 87·1% (85·6–88·4) for the accelerated epirubicin group, and 86·5% (85·0–87·9) for the CMF group versus 86·7% (85·2–88·1) for the capecitabine group. When the HR for TTR events was adjusted for factors known to affect prognosis (age, vascular invasion, oestrogen-receptor status, HER2 status, nodal status, tumour grade, and tumour size) the value for accelerated epirubicin compared with standard epirubicin was 0·95 (95% CI 0·82–1·10; p=0·48). We found no evidence of heterogeneity in treatment effect by subsequent treatment with CMF or capecitabine or clinical subgroups (figure 3). After adjustment for randomisation stratification factors and allocation to either standard or accelerated epirubicin, the HR for capecitabine compared with CMF was 1·01 (95% CI 0·87–1·17, p=0·88). No evidence of heterogeneity in treatment effect was seen by previous treatment with standard or accelerated epirubicin or clinical subgroups (figure 3).

601 (14%) patients died (table 2; figure 2). Nine deaths were reported to be due to infection related to chemotherapy (CMF n=8, capecitabine n=1), but none of these occurred during epirubicin treatment. Similarly, overall survival did not differ between the CMF and capecitabine groups (figure 2). No evidence of heterogeneity in treatment effect was seen according to the other randomised treatment or the other clinical subgroups (data not shown). Results for other secondary clinical outcome endpoints, such as invasive-disease-free survival and time to distant tumour recurrence, were consistent with those presented for TTR (data not shown). Overall survival at 3 years was 95·5% (95% CI 94·5–96·2) in the standard epirubicin groups versus 94·4% (93·4–95·3) in the accelerated epirubicin groups, and 95·2% (94·3–96·1) in the CMF groups versus 94·7% (93·6–95·5) in the capecitabine groups. At 5 years, overall survival was 90·7% (95% CI 89·4–91·8) in the standard epirubicin groups versus 89·7% (88·3–90·9) in the accelerated epirubicin groups, and 90·2% (88·9–91·4) in the CMF groups versus 90·1% (88·8–91·3) in the capecitabine groups.

Of the 1399 premenopausal women with data available on ovarian function, disruption or discontinuation of periods during chemotherapy was significantly higher with accelerated epirubicin than standard epirubicin (558 [83%] of 672 *vs* 556 [76%] of 728; p=0·0020) and with CMF than capecitabine (593 [86%] of 687 *vs* 520 [73%] of 712; p<0·0001). 18 months after randomisation, the proportions of patients with permanently discontinued periods did not differ between the epirubicin groups (standard epirubicin 345 [49%] of 710 and accelerated epirubicin 305 [47%] of 651; p=0·52). Between the CMF and capecitabine groups, however, the proportions of patients with permanently discontinued periods at 18 months remained significantly different (CMF 499 [75%] of 667 and capecitabine 294 [42%] of 695; absolute difference in proportions 32·5%, 95% CI 27·5–37·4, p<0·0001), as did those for patients receiving adjuvant ovarian suppression (67 [10%] of 687 and 133 [19%] of 713; 8·9%, 5·3–12·5, p<0·0001).

The quality-of-life and toxicity substudy included 2121 patients, 2115 (99·7%) of whom received at least one cycle of allocated chemotherapy (table 3, appendix pp 13–19). The proportion of completed quality-of-life questionnaires received at 12 months from those who consented to particpate in the quality-of-life substudy was 172 (53%) of 326 patients in the standard epirubicin and CMF group, 181 (58%) of 314 in the accelerated epirubicin and CMF group, 206 (65%) of 319 in the standard epirubicin and capecitabine group, and 208 (65%) of 322 in the accelerated epirubicin and capecitabine group. During cycles one to four of chemotherapy, the numbers of adverse events of grade 3 or worse were lower in the accelerated epirubicin group than in the standard epirubicin group for leucopenia, neutropenia, and febrile neutropenia (table 3). However, there were significantly more cases in the accelerated epirubicin group of grade 3 or worse hand–foot syndrome and any grade anaemia, arthralgia, and back pain (table 3). The number of hospital admissions for neutropenic sepsis during chemotherapy cycles one to four was higher among patients receiving standard epirubicin than among those receiving accelerated epirubicin (appendix p 20).

**Table 3:** Adverse events during cycles one to four of chemotherapy among patients in the quality-of-life and toxicity substudy

	**Standard epirubicin (n=1070)**			**Accelerated epirubicin (n=1045)**			p value for trend*	**p value for events grade ≥3**
	Grade 1–2	Grade 3	Grade 4	Grade 1–2	Grade 3	Grade 4		
**Events prespecified on case report form**								
Fatigue	919 (86%)	52 (5%)	4 (<0·5%)	889 (85%)	62 (6%)	1 (<0·5%)	0·16	0·45
Nausea	806 (75%)	19 (2%)	1 (<0·5%)	801 (77%)	23 (2%)	0	0·24	0·65
Vomiting	361 (34%)	23 (2%)	1 (<0·5%)	338 (32%)	20 (2%)	2 (<0·5%)	0·47	0·88
Alopecia†	1023 (96%)	0	0	995 (95%)	0	0	0·43	..
Neuropathy	132 (12%)	1 (<0·5%)	0	113 (11%)	2 (<0·5%)	0	0·27	0·62
Mucositis/stomatitis	723 (68%)	10 (1%)	0	718 (69%)	13 (1%)	0	0·66	0·53
Hand–foot syndrome	36 (3%)	0	0	80 (8%)	9 (1%)	0	<0·0001	0·0017
Skin	291 (27%)	3 (<0·5%)	0	309 (30%)	4 (<0·5%)	0	0·084	0·72
Superficial thrombophlebitis†	247 (23%)	0	0	232 (22%)	0	0	0·63	..
Diarrhoea	324 (30%)	11 (1%)	0	359 (34%)	14 (1%)	0	0·061	0·55
Constipation	696 (65%)	7 (1%)	0	695 (67%)	4 (<0·5%)	0	0·46	0·55
Infection	330 (31%)	34 (3%)	4 (<0·5%)	296 (28%)	33 (3%)	1 (<0·5%)	0·18	0·72
Anaemia	264 (25%)	0	0	421 (40%)	1 (<0·5%)	0	<0·0001	0·49
Leucopenia	276 (26%)	37 (3%)	4 (<0·5%)	35 (3%)	8 (1%)	2 (<0·5%)	<0·0001	<0·0001
Neutropenia	318 (30%)	125 (12%)	50 (5%)	46 (4%)	11 (1%)	7 (1%)	<0·0001	<0·0001
Thrombocytopenia	11 (1%)	1 (<0·5%)	1 (<0·5%)	30 (3%)	0	0	0·10	0·50
Febrile neutropenia	0	34 (3%)	4 (<0·5%)	0	14 (1%)	1 (<0·5%)	0·0019	0·002
**Other adverse events**‡								
Alanine aminotransferase increased	20 (2%)	1 (<0·5%)	0	50 (5%)	2 (<0·5%)	0	0·00018	0·62
Arthralgia	30 (3%)	1 (<0·5%)	0	58 (6%)	2 (<0·5%)	0	0·0022	0·62
Back pain	22 (2%)	0	0	40 (4%)	1 (<0·5%)	0	0·0028	0·49
Blood alkaline phosphatase	0	0	0	8 (1%)	0	0	0·0017	..
Blood alkaline phosphatase increased	4 (<0·5%)	0	0	33 (3%)	0	0	<0·0001	..
Cough	53 (5%)	0	0	68 (7%)	1 (<0·5%)	0	0·17	0·49
Dry eye	33 (3%)	0	0	17 (2%)	0	0	0·023	..
Dry mouth	44 (4%)	0	0	57 (5%)	1 (<0·5%)	0	0·11	0·49
Dysgeusia	146 (14%)	1 (<0·5%)	0	165 (16%)	1 (<0·5%)	0	0·20	>0·99
Dyspepsia	234 (22%)	1 (<0·5%)	0	268 (26%)	3 (<0·5%)	0	0·078	0·37
Epistaxis	7 (1%)	0	0	22 (2%)	0	0	0·0039	..
Haemorrhoids	10 (1%)	0	0	25 (2%)	0	0	0·0066	..
Headache	97 (9%)	5 (<0·5%)	0	114 (11%)	2 (<0·5%)	0	0·14	0·45
Lacrimation increased	67 (6%)	0	0	89 (9%)	0	0	0·10	..
Liver function test abnormal	27 (3%)	3 (<0·5%)	0	41 (4%)	3 (<0·5%)	0	0·10	>0·99
Lymphoedema	23 (2%)	2 (<0·5%)	0	11 (1%)	0	0	0·028	0·50
Oropharyngeal pain	42 (4%)	1 (<0·5%)	1 (<0·5%)	53 (5%)	1 (<0·5%)	0	0·23	>0·99
Tearfulness	0	0	0	6 (1%)	0	0	0·0071	..

Adverse events were graded with the Common Terminology Criteria for Adverse Events version 3.0. Events are shown that meet at least one one of the following criteria: difference in proportion of patients reporting an event of any grade is >1% between the epirubicin groups; the proportion of patients experiencing an event of any grade is >10% in either the standard epirubicin or the accelerated epirubicin group; and the difference between epirubicin groups in the proportion of patients experiencing an event of any grade is significant (p<0·01). No patients died from these events (grade 5).

* Trend tests combine grade 3–5 adverse events.

† Common Terminology Criteria for Adverse Events grades 3 and 4 are not applicable.

‡ Free-text preferred terms of Medical Dictionary for Regulatory Activities version 10 are used.

Among the toxicity and quality-of-life substudy patients, 2074 (98%) of 2121 patients received at least one cycle of allocated CMF or capecitabine chemotherapy (table 4, appendix pp 21–27). During cycles five to eight of chemotherapy, the proportion of patients who had grade 3 or worse adverse events was significantly greater in the CMF group for fatigue, nausea, mucositis/stomatitis, thrombosis/embolism, infection, anaemia, leucopenia, neutropenia, thrombocytopenia, and febrile neutropenia than in the capecitabine group, whereas grade 3 or worse hand–foot syndrome was more prevalent in the capecitabine group.

**Table 4:** Adverse events during cycles five to eight of chemotherapy among patients in the quality-of-life and toxicity substudy

	**CMF (n=1030)**			**Capecitabine (n=1044)**			p value for trend*	**p value for events grade ≥3**
	Grade 1–2	Grade 3	Grade 4	Grade 1–2	Grade 3	Grade 4		
**Events prespecified on case report form**								
Fatigue	825 (80%)	107 (10%)	2 (<0·5%)	773 (74%)	52 (5%)	0	<0·0001	<0·0001
Nausea	675 (66%)	21 (2%)	2 (<0·5%)	548 (52c%)	7 (1%)	0	<0·0001	0·0029
Vomiting	303 (29%)	23 (2%)	0	203 (19%)	14 (1%)	1 (<0·5%)	<0·0001	0·19
Alopecia†	963 (93%)	0	0	902 (86%)	0	0	<0·0001	..
Neuropathy	162 (16%)	2 (<0·5%)	0	225 (22%)	2 (<0·5%)	1 (<0·5%)	0·00027	>0·99
Mucositis/stomatitis	617 (60%)	48 (5%)	4 (<0·5%)	426 (41%)	9 (1%)	1 (<0·5%)	<0·0001	<0·0001
Hand–foot syndrome	118 (11%)	3 (<0·5%)	0	649 (62%)	129 (12%)	0	<0·0001	<0·0001
Skin	209 (20%)	0	0	289 (28%)	4 (<0·5%)	0	0·89	0·12
Thrombosis/embolism	16 (2%)	14 (1%)	7 (1%)	14 (1%)	5 (<0·5%)	2 (<0·5%)	0·014	0·0074
Superficial thrombophlebitis†	162 (16%)	0	0	144 (14%)	0	0	0·21	..
Diarrhoea	434 (42%)	44 (4%)	2 (<0·5%)	490 (47%)	63 (6%)	4 (<0·5%)	0·0069	0·053
Constipation	476 (46%)	4 (<0·5%)	0	189 (18%)	2 (<0·5%)	0	<0·0001	0·45
Infection	260 (25%)	96 (9%)	6 (1%)	200 (19%)	18 (2%)	0	<0·0001	<0·0001
Anaemia	411 (40%)	29 (3%)	1 (<0·5%)	250 (24%)	4 (<0·5%)	2 (<0·5%)	<0·0001	<0·0001
Leucopenia	376 (37%)	95 (9%)	80 (8%)	136 (13%)	7 (1%)	1 (<0·5%)	<0·0001	<0·0001
Neutropenia	263 (26%)	155 (15%)	166 (16%)	165 (16%)	17 (2%)	6 (1%)	<0·0001	<0·0001
Thrombocytopenia	109 (11%)	13 (1%)	3 (<0·5%)	32 (3%)	1 (<0·5%)	1 (<0·5%)	<0·0001	0·0065
Febrile Neutropenia	0	96 (9%)	19 (2%)	0	7 (1%)	1 (<0·5%)	<0·0001	<0·0001
**Other adverse events**‡								
Abdominal pain	24 (2%)	0	0	33 (3%)	3 (<0·5%)	0	0·15	0·25
Alanine aminotransferase increased	54 (5%)	2 (<0·5%)	1 (<0·5%)	33 (3%)	3 (<0·5%)	0	0·026	>0·99
Arthralgia	56 (5%)	0	0	39 (4%)	0	0	0·074	..
Blood bilirubin	0	0	0	9 (1%)	0	0	0·0038	..
Chest pain	5 (<0·5%)	0	0	15 (1%)	3 (<0·5%)	0	0·010	0·25
Chills	14 (1%)	0	0	1 (<0·5%)	0	0	0·00046	..
Cough	50 (5%)	0	0	37 (4%)	0	0	0·15	..
Dry mouth	60 (6%)	1 (<0·5%)	0	80 (8%)	1 (<0·5%)	0	0·10	>0·99
Dysgeusia	112 (11%)	2 (<0·5%)	0	116 (11%)	0	0	>0·99	0·25
Dyspepsia	107 (10%)	0	0	78 (8%)	0	0	0·021	..
Dyspnoea	66 (6%)	7 (1%)	2 (<0·5%)	31 (3%)	1 (<0·5%)	2 (<0·5%)	<0·0001	0·089
Epistaxis	28 (3%)	0	0	10 (1%)	0	0	0·0029	..
Eye pain	39 (4%)	0	0	17 (2%)	0	0	0·0027	..
Foreign body sensation in eyes	29 (3%)	0	0	9 (1%)	0	0	0·00090	..
Hot flush	94 (9%)	1 (<0·5%)	1 (<0·5%)	55 (5%)	0	0	0·00038	0·25
Insomnia	42 (4%)	0	0	24 (2%)	2 (<0·5%)	0	0·048	0·50
Lacrimation increased	81 (8%)	0	0	52 (5%)	1 (<0·5%)	0	0·012	>0·99
Lymphoedema	31 (3%)	0	0	19 (2%)	0	0	0·086	..
Nasopharyngitis	32 (3%)	0	0	13 (1%)	0	0	0·0039	..
Non-cardiac chest pain	6 (1%)	2 (<0·5%)	0	11 (1%)	7 (1%)	1 (<0·5%)	0·051	0·11
Oral candidiasis	13 (1%)	1 (<0·5%)	0	1 (<0·5%)	0	0	0·00046	0·50
Pyrexia	38 (4%)	0	0	14 (1%)	0	0	0·00064	..
Vision blurred	11 (1%)	0	0	1 (<0·5%)	0	0	0·0032	..

Adverse events were graded with the Common Terminology Criteria for Adverse Events version 3.0. Events are shown that meet at least one of the following criteria: difference in proportion of patients reporting an event of any grade is >1% between the CMF and capecitabine groups; the proportion of patients experiencing an event of any grade is >10% in either the CMF or capecitabine group; and the difference between the CMF and capecitabine groups in the proportion of patients experiencing an event of any grade is significant (p<0·01). Three substudy patients in the CMF group died from infections (grade 5 adverse events). Table 2 shows cause of death data for the whole cohort. CMF=cyclophosphamide, methotrexate, and fluorouracil.

* Trend tests combine grade 3–5 adverse events.

† Common Terminology Criteria for Adverse Events grades 3 and 4 are not applicable.

‡ Free-text preferred terms of Medical Dictionary for Regulatory Activities version 10 are used.

**Figure 4:**
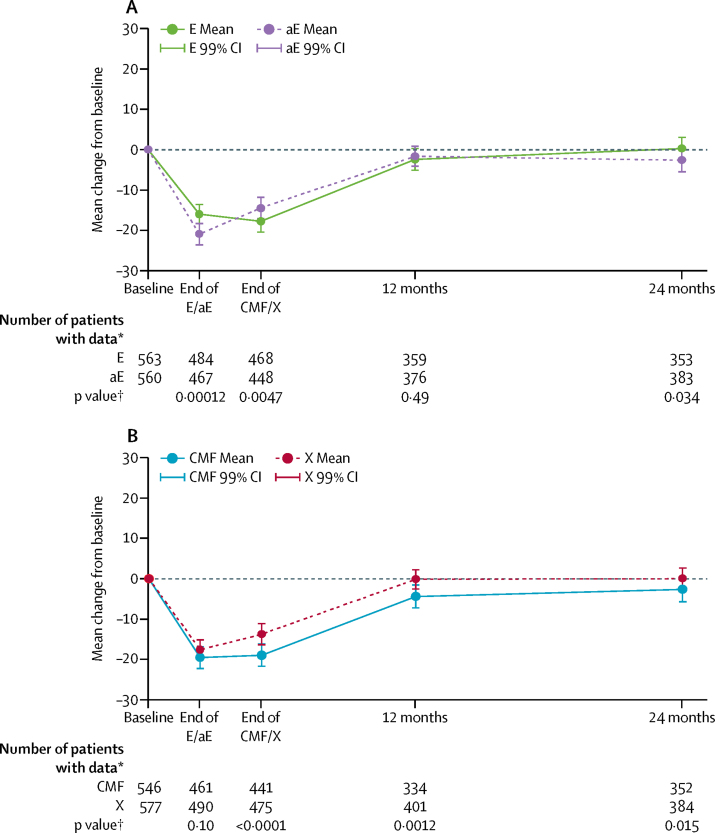
Mean change in scores on the EORTC QLQ-C30 global health status quality-of-life scale from baseline. by treatment EORTC=European Organisation for Research and Treatment of Cancer. E=standard epirubicin. aE=accelerated epirubicin. CMF=cyclophosphamide, methotrexate, and fluorouracil. X=capecitabine. *At baseline and all timepoints of interest. †p value from ANCOVA analysis of E versus aE and for CMF versus X, adjusted for baseline subscale score.

At the end of epirubicin treatment, accelerated epirubicin was associated with significantly worse self-reported tingling, numb, or sore hands and feet (consistent with clinician-reported acute adverse events) than standard epirubicin. By contrast, patients receiving standard epirubicin reported more weight gain (appendix pp 34–40). These differences did not persist in assessments at the end of CMF or capecitabine treatment or at 12 and 24 months. In the cross-sectional analysis at the end of epirubicin treatment, EORTC QLQ-C30 GHS/QOL was significantly worse among patients who had received accelerated epirubicin than among those who had received standard epirubicin (mean scores 54·3, 95% CI 52·3–56·3 *vs* 59·4, 57·8–61·2, respectively). These differences did not persist over time (at 12 months, 72·4–76·2 *vs* 73·6, 71·7–75·5; at 24 months, 74·3, 72·3–76·2 *vs* 76·9, 75·0–78·8, figure 4). A higher proportion of patients receiving accelerated epirubicin than those receiving standard epirubicin had a clinically meaningful deterioration of ten points or more in EORTC QLQ-C30 GHS/QOL from baseline (313 [67%] of 467 *vs* 278 [57%] of 484). Longitudinal analysis with generalised estimating equations models showed no differences in quality of life over time (p=0·96, appendix p 41).

At the end of CMF or capecitabine treatment, self-reported tiredness, constipation, sore mouth, mouth ulcers, and breathlessness were worse in the CMF group, and dry, flaky, or sensitive skin and tingling, numb, or sore hands and feet were worse in the capecitabine group (appendix pp 34–40). The severity of self-reported diarrhoea did not differ between groups. Patients receiving CMF had significantly worse pain in muscles, bones, and joints at 12 months and worse tiredness at 24 months than those in the capecitabine group (appendix pp 34–40). EORTC QLQ-C30 GHS/QOL was significantly worse at the end of CMF treatment than at the end of capecitabine treatment (mean scores 57·3, 95% CI 55·2–59·4 *vs* 62·4, 60·6–64·3) and at 12 and 24 months (12 months 72·1, 70·1–74·1 *vs* 75·5, 73·7–77·3; 24 months 73·8, 71·7–75·8 *vs* 77·1, 75·3–79·0); analysis of change from baseline showed a similar pattern (figure 4). A higher proportion of patients receiving CMF than those receiving capecitabine had a clinically meaningful deterioration of ten points or more in EORTC QLQ-C30 GHS/QOL scores both from baseline and end of treatment (255 [58%] of 441 patients receiving CMF *vs* 235 [50%] of 475 receiving capecitabine; p=0·011) and at 12 months (114 [34%] of 334 *vs* 89 [22%] of 401; p<0·001). Longitudinal analysis with generalised estimating equations models confirmed that patients receiving CMF had a significantly worse quality of life over time (p<0·0001, appendix p 41). No significant interaction was found between the previous epirubicin regimen and CMF or capecitabine treatment (appendix p 41).

The proportions of patients with grade 3 or worse late adverse events 12 months or later after randomisation did not differ significantly between the standard and accelerated epirubicin groups, irrespective of whether or not events were prespecified (table 5, appendix pp 28–29). Likewise, we found no significant differences in the proportion of patients who had late grade 3 or worse adverse events between the CMF and capecitabine groups (table 6, appendix pp 30–31). A significant proportion of patients in the capecitabine groups than in the CMF groups had late hand–foot syndrome of any grade (211 [10%] of 2172 *vs* 90 [4%] of 2176, p<0·0001).

**Table 5:** Late adverse events by epirubicin regimen

	**Standard epirubicin (n=2193)**			**Accelerated epirubicin (n=2155)**			p value for trend*	**p value for events grade ≥3**
	Grade 1–2	Grade 3	Grade 4	Grade 1–2	Grade 3	Grade 4		
**Events prespecified on case-report form**								
Fatigue	773 (35%)	26 (1%)	3 (<0·5%)	764 (36%)	15 (1%)	2 (<0·5%)	0·63	0·10
**Other adverse events**†								
Anxiety	25 (1%)	1 (<0·5%)	0	7 (<0·5%)	1 (<0·5%)	0	0·0029	>0·99
Arthralgia	229 (10%)	11 (1%)	1 (<0·5%)	234 (11%)	8 (<0·5%)	0	0·81	0·50
Hot flush	250 (11%)	12 (1%)	0	212 (10%)	6 (<0·5%)	1 (<0·5%)	0·066	0·36

Analysis of late safety compared all signs and symptoms reported at or after 12 months from randomisation and included all patients who were randomly assigned treatment, followed up for at least 9 months, and received at least one cycle of allocated chemotherapy. Late safety analysis was censored at the point of disease recurrence or second primary cancer. Adverse events were graded with the Common Terminology Criteria for Adverse Events version 3.0. Events are shown that meet at least one of the following criteria: difference in proportion of patients reporting an event is >1% between epirubicin groups; the proportion of patients experiencing the event is >10% in either group; the difference between epirubicin groups in the proportion experiencing an event is significant (p<0·01). No patients died from these events (grade 5).

* Trend tests combine grade 3–5 adverse events.

† Free-text preferred terms of Medical Dictionary for Regulatory Activities version 10 are used.

**Table 6:** Late adverse events by CMF or capecitabine group

	**CMF (n=2176)**			**Capecitabine (n=2172)**			p value for trend*	**p value for events grade ≥3**
	Grade 1–2	Grade 3	Grade 4	Grade 1–2	Grade 3	Grade 4		
**Events prespecified on case-report form**								
Fatigue	781 (36%)	21 (1%)	2 (<0·5%)	758 (35%)	20 (1%)	3 (<0·5)	0·14	>0·99
Hand–foot syndrome	89 (4%)	1 (<0·5%)	0	208 (10%)	3 (<0·5%)	0	<0·0001	0·37
**Other adverse events**†								
Arthralgia	263 (12%)	13 (1%)	0	200 (9%)	6 (<0·5%)	1 (<0·5%)	0·0010	0·26
Hot flush	253 (12%)	7 (<0·5%)	0	209 (10%)	11 (<0·5%)	1 (<1%)	0·066	0·26
Joint stiffness	50 (2%)	0	0	28 (1%)	0	0	0·016	..
Nail disorder	24 (1%)	0	0	72 (3%)	0	0	<0·0001	..
Onychoclasis	7 (<0·5%)	0	0	21 (1%)	0	0	0·0079	..

Analysis of late safety compared all signs and symptoms reported at or after 12 months from randomisation and included all patients randomly assigned treatment, followed up for at least 9 months, and received at least one cycle of allocated chemotherapy. Late safety analysis was censored at the point of disease recurrence or second primary cancer. Adverse events were graded with the Common Terminology Criteria for Adverse Events version 3.0. Events are shown that meet at least one of the following criteria: difference in proportion of patients reporting event is >1% between CMF and capecitabine groups; the proportion of patients experiencing an event of any grade is >10% in either the CMF or capecitabine group; and the difference between the CMF and capecitabine groups in the proportion experiencing an event is significant (p<0·01). No patients died from these events (grade 5). CMF=cyclophosphamide, methotrexate, and fluorouracil.

* Trend tests combine grade 3–5 adverse events.

† Free-text preferred terms of Medical Dictionary for Regulatory Activities version 10 are used.

## Discussion

In the TACT2 trial, despite achieving the intended 50% increase in epirubicin dose density, our findings did not support the hypothesis that this would lead to significant improvements in breast cancer outcomes. The two treatment groups were well balanced for standard clinicopathological factors likely to influence outcomes, and we found no evidence of heterogeneity of effect by breast cancer subtype or nodal status. By contrast, our results support the second hypothesis, that capecitabine can be safely substituted for CMF, when given after single-agent epirubicin, without loss of efficacy and with improvements in toxicity profile and overall quality of life. We found no evidence in any subgroup of significantly inferior efficacy. Patients randomly assigned to capecitabine had fewer adverse events in most categories, with more adverse events seen than with CMF in only three: diarrhoea, hand–foot syndrome, and neuropathy. For diarrhoea, which is a known side-effect of capecitabine, patients' self-reported severity did not differ significantly between the CMF and capecitabine treatment groups.

Patients in all groups had 5-year overall survival of around 90% with no unexpected adverse events. Fatal adverse events were rare and occurred at similar frequencies in all groups, although numerically more deaths due to infection were seen among patients receiving CMF and more vascular deaths were seen among patients receiving capecitabine. These deaths serve as a reminder that adjuvant chemotherapy is toxic. Thus, although even with an improved toxicity profile, use of capecitabine did not prevent all treatment-related deaths.

The results of our analysis of accelerated versus standard epirubicin might seem to be at odds with those from other studies. However, a meta-analysis of 3418 patients in four trials that included the CALGB 9741 study showed an overall significant benefit in terms of survival free from invasive disease for accelerated chemotherapy (HR 0·85, 95% CI 0·73–0·95),^14^ an estimate consistent with the data reported in TACT2, which assessed a larger population than these four trials combined. The CALGB 9741 study^3^ reported a benefit in patients who received adjuvant chemotherapy with increased dose density compared with standard therapy, but in contrast to TACT2, the anthracycline and the taxane components were both accelerated. In the Italian GIM2 study^15^ better outcomes were also reported with accelerated chemotherapy than with standard chemotherapy. The CALGB 9840 study has further shown inferiority of paclitaxel given every 3 weeks compared with when given once per week.^16^ In both of these studies, however, how much of the reported benefits were due to the altered paclitaxel schedule and how much to the accelerated administration of the anthracycline and cyclophosphamide-based components remains unclear. The TACT2 data indicate that acceleration of only the anthracycline component alone provides little benefit to patients.

The TACT2 population differs from the populations in other trials in several ways. 54% of TACT2 patients had involved axillary nodes, whereas CALGB 9741^3^ and GIM2^15^ only enrolled women with confirmed axillary involvement. 40% of patients in CALGB 9741 had more than three involved nodes compared with only 12% in TACT2. The benefits seen in the CALGB 9741 and GIM2 studies, therefore, might have been driven by the inclusion of higher-risk populations, and particularly patients with extensive nodal involvement. This possibility is supported by data from the AGO-ETC trial,^17^ in which the HR at 5 years for a dose-dense schedule was 0·64 among patients with ten or more involved nodes, compared with 0·79 for patients with four to nine involved nodes, although the difference was not significant. However, despite what other studies have suggested, we saw no benefit from dose-dense chemotherapy in patients with more than ten involved nodes (HR 0·82, 95% CI 0·51–1·31). This difference between findings highlights the need for a meta-analysis of individual patients' data to determine whether or not traditional prognostic factors, such as extent of nodal involvement, identify subgroups of patients who will gain increased benefit from accelerated chemotherapy (table 7).

**Table 7:** Risk of disease recurrence by nodal status in trials of dose-dense chemotherapy

	**Node negative**	**1–3 nodes**	**>3 nodes**
TACT2	1·00 (0·78–1·28)	0·87 (0·69–1·09)	0·97 (0·72–1·30)
GIM2^15^*	N/A	0·88 (0·67–1·11)	0·68 (0·54–0·86)
AGO ETC^17^	N/A	N/A	0·72 (0·59–0·87)

Data are hazard ratios (95% CI) for the primary endpoint (recurrence) by nodal subgroups. N/A=not applicable.

* Data supplied by authors on request; similar data for CALGB 9741 were unavailable.

In TACT2, patients with HER2-positive tumours had access to routine treatment with trastuzumab upon completion of their chemotherapy, which was given to 761 (92%) of 831 atients with known HER2-positive disease. Of the other studies, GIM2 was the only one to be done in the trastuzumab era, but only 130 (27%) of 480 patients with HER2-positive disease received this drug, which constituted 6% of the overall trial population (compared with 17% in our study), and in this small group no benefit was associated with accelerated chemotherapy.^15^ This difference alone, however, cannot explain the absence of benefit from accelerated chemotherapy in TACT2.

We had anticipated that epirubicin given every 2 weeks in our accelerated regimen would be associated with reduced risk of toxicity because of the use of primary prophylactic granulocyte-colony-stimulating factor. Apart from the expected significant reduction in neutropenia and febrile neutropenia, patients in the accelerated epirubicin group had more adverse events, particularly low-grade events, than those in the standard epirubicin group. The overall quality of life was worse during accelerated epirubicin treatment than during standard epirubicin treatment, although this difference did not persist after this treatment ended.

Although other studies have tested the efficacy of additional capecitabine in early breast cancer (FINXX^18^, GBG - GeparQuattro^19^, CREATE-X^20^), only one other study of which we are aware, the CALGB 49907 study,^21^ has compared CMF and capecitabine as adjuvant chemotherapy for breast cancer, and that trial showed inferior outcomes for patients assigned capecitabine. Older women at increased risk of recurrence were assigned to receive standard chemotherapy (a choice of four cycles of cyclophosphamide and doxorubicin or six cycles of CMF) or single-agent capecitabine at a reduced dose of 2000 mg/m^2^ per day. The study was stopped early due to inferiority of capecitabine when just over 600 patients had been enrolled, of whom less than half of the standard chemotherapy group had opted for CMF. The delivery of the chemotherapy seems to have been challenging in both groups. The overall poorer result for the capecitabine group was based on an early analysis (18 *vs* 24 distant recurrence events). An updated analysis suggested that the difference in efficacy between the two groups had reduced, and might only be significant in patients with hormone-receptor-negative tumours.^22^ In the TACT2 trial, we used capecitabine after anthracycline chemotherapy, which might explain further the difference between our results and those in the CALGB 49997 study.

We conducted the acute substudy in only a large subset of patients since both regimens were well characterised clinically and the number needed to assess patient-reported outcomes and adverse events was not as large as that required for assessing the efficacy endpoint. This pragmatic solution aimed to maximise trial efficiency and is not seen as a limitation to the interpretation of the study.

Defining whether a toxicity profile is better for one treatment than another from the patients' perspective is not as straightforward as assessing relative efficacy with one disease-based endpoint, such as TTR. Patients, nurses, and doctors might classify the relevance of toxicity differently, and the inclusion of patient-reported adverse events is recommended to supplement clinician reporting.^23^, ^24^, ^25^ From a medical perspective, of the 12 adverse events for which the frequency of grade 3 or 4 events differed significantly between the CMF and capecitabine groups only two were more frequent with capecitabine (table 4). The differences are what would be expected from these therapies, with fatigue, nausea/vomiting, gastrointestinal and bone marrow events, and thromboembolism all being more frequent in patients receiving CMF, whereas only diarrhoea and hand–foot syndrome were more frequent in patients receiving capecitabine.

How to synthesise from a patient's perspective a shift in toxicity profile between two similarly effective regimens is a conundrum. For some patients, although toxicities were numerically fewer with capecitabine, experiencing grade 3–4 diarrhoea while taking this drug might be less acceptable than, for example, experiencing grade 3–4 nausea, fatigue, and asymptomatic neutropenia while taking CMF.

Quality-of-life scales are designed to measure overall burden on patients, globally and within various subdomains. Our hypothesis that capecitabine would have better tolerability than CMF is confirmed by the main quality-of-life outcome, which showed worse self-reported global quality of life at the end of CMF treatment than at the end of capecitabine treatment, and this difference persisted at 12 and 24 months. Although the numbers of patients who completed quality-of-life questionnaires differed between groups, additional analyses suggested that at 12 months these differences were not associated with physician-reported adverse events at the same timepoint (data not shown).

An important difference in the toxicity profiles of capecitabine and CMF was the recovery of menstrual function in women who were premenopausal at enrolment. Evidence suggests that recovery of function could have resulted in a slightly less active endocrine regimen,^26^ but our findings do not allow us to draw conclusions on the relation between this and disease-related outcome. Importantly, recovery of menstrual function might be of benefit to younger women, given the increasing age at which many are starting families, with increasing numbers of women with early breast cancer requiring chemotherapy but also wishing to preserve their fertility. Although we do not know the numbers of women in this study who maintained fertility, the increased frequency of return of menses among those receiving capecitabine seems to offer this as an option to try and preserve ovarian function. Using ovarian suppression during chemotherapy to preserve menstrual function has had mixed results.^27^

There is no one universal standard regimen for adjuvant chemotherapy in breast cancer. In the TACT2 study, we used a UK regimen of epirubicin followed by CMF that was standard care at the time. This regimen, along with fluorouracil, epirubicin, and cyclophosphamide chemotherapy, was compared in the TACT trial^4^ with an anthracycline and taxane regimen, against which there was no evidence of inferiority. In TACT2 we have shown that increasing the dose density of the anthracycline component of chemotherapy did not improve disease outcomes or quality of life. However, we did confirm that capecitabine may be used in place of CMF with reduced overall toxicity. For the adjuvant treatment of early breast cancer, in patients in whom taxanes are not indicated or contraindicated, treatment with epirubicin followed by capecitabine in 3-week cycles is an effective, safe, and well tolerated option.
