# Supplementary material for: Accelerated versus standard epirubicin followed by cyclophosphamide, methotrexate, and fluorouracil or capecitabine as adjuvant therapy for breast cancer in the randomised UK TACT2 trial (CRUK/05/19): a multicentre, phase 3, open-label, randomised, controlled trial
Source: Lancet Oncol. 2017 Jul;18(7):929–45. doi: 10.1016/S1470-2045(17)30404-7 (PMC5489700; doi:10.1016/S1470-2045(17)30404-7)
Supplement: Supplementary appendix [file mmc1.pdf]

THE LANCET
Oncology

Supplementary appendix

This appendix formed part of the original submission and has been peer reviewed. We post it as supplied by the authors.

Supplement to: Cameron D, Morden JP, Canney P, et al, on behalf of the TACT2 Investigators. Accelerated versus standard epirubicin followed by cyclophosphamide, methotrexate, and fluorouracil or capecitabine as adjuvant therapy for breast cancer in the randomised UK TACT2 trial (CRUK/05/19): a multicentre, phase 3, open-label, randomised, controlled trial. *Lancet Oncol* 2017; published online June 6. http://dx.doi.org/10.1016/S1470-2045(17)30404-7.

Web appendix

Table A1 - List of TACT2 participating centres and number of patients recruited

Recruiting site	Principal investigator (original)	Principal investigator (current)	Patients recruited
Beatson Oncology Centre	Peter Canney	Iain MacPherson	290
Christie Hospital, Manchester	Andrew Wardley	Andrew Wardley	284
Southampton General Hospital	Nicholas Murray	Peter Simmonds	128
Royal Surrey County Hospital	Robert Laing	Robert Laing	115
Singleton Hospital, Swansea	Gianfilippo Bertelli	Gianfilippo Bertelli	109
Royal Shrewsbury Hospital	Rajiv Agrawal	Laura Pettit	104
University Hospital of North Staffordshire, Stoke-on-Trent	Adrian Murray Brunt	Adrian Murray Brunt	100
Broomfield Hospital, Chelmsford	Neville Davidson	Sunil Skaria	95
Western General Hospital, Edinburgh	Angela Bowman	Angela Bowman	94
Torbay District General Hospital	Peter Bliss	Peter Bliss	89
Royal Devon and Exeter Hospital, Exeter	Andrew Goodman	Andrew Goodman	84
Falkirk and District Royal Infirmary	Peter Canney	Judith Fraser	80
University Hospital, Coventry	Robert Grieve	Robert Grieve	75
Bradford Royal Infirmary	Chris Bradley	Chris Bradley	67
Southend General Hospital	Anne Robinson	Hafiz Algurafi	66
Newcastle General Hospital, NCCT	Mark Verrill	Mark Verrill	65
Essex County Hospital	Philip Murray	Mukesh Bindlish Mukesh	62
Norfolk and Norwich University Hospital	Adrian Harnett	Adrian Harnett	61
Weston Park Hospital, Sheffield	Robert Coleman	Robert Coleman	61
North Cheshire Hospitals, Halton and Warrington	Susan O'Reilly	Peter Clark	59
Wishaw General Hospital, Wishaw	Jonathan Hicks	Jonathan Hicks	57
Crosshouse Hospital, Kilmarnock	Lucy Scott	Lucy Scott	55
Royal Sussex County Hospital, Brighton	David Bloomfield	David Bloomfield	54
Maidstone Hospital	Catherine Harper-Wynne	Catherine Harper-Wynne	53
Queen Elizabeth Hospital, King’s Lynn	Athar Ahmad	Athar Ahmad	53
Queen’s Hospital, Burton-on-Trent	Shan Chetiyawardana	Mojca Persic	50
Guy’s Hospital	Paul Ellis	Paul Ellis	47
Hairmyres Hospital, East Kilbride	Hosney Yosef	Grainne Dunn	47
Macclesfield District General Hospital	Lisa Barraclough	Lisa Barraclough	47
Royal Liverpool University Hospital	Susan O’Reilly	Susan O’Reilly	47
Bristol Haematology and Oncology Centre	Jeremy Braybrooke	Jeremy Braybrooke	46
Wansbeck Hospital, Ashington	Daniela Lee	Anthony Branson	46
Charing Cross Hospital	Charles Lowdell	Susan Cleator	44
Royal Preston Hospital	Elaine Young	Elaine Young	44
St Bartholomew’s Hospital, London	Chris Gallagher	Chris Gallagher	43
Blackpool Victoria Hospital	Andrew Hindley	Pavel Bezecny	42
Royal Cornwall Hospital, Treliske	Duncan Wheatley	Duncan Wheatley	42
Royal Hampshire County Hospital	Nicholas Murray	Sanjay Ray	42
North Devon District Hospital	Mark Napier	Mark Napier	40
Russells Hall Hospital, Dudley	Rozenn Allerton	Rozenn Allerton	40
Aberdeen Royal Infirmary	Radha Todd	Ravi Sharma	37
North Tyneside General Hospital	Anthony Branson	Radha Todd	37
Derbyshire Royal Infirmary	Pamela Woodings	Pamela Woodings	35
Queen Margaret Hospital, Dunfirmline	Angela Bowman	Angela Bowman	35
Ninewells Hospital, Dundee	Douglas Adamson	Douglas Adamson	34
Diana, Princess of Wales Hospital, Grimsby	Sunil Upadhyay	Sunil Upadhyay	33
Dumfries and Galloway Royal Infirmary	Tamasin Evans	Marjory Maclennan	33
Addenbrooke’s Hospital, Cambridge	Karen McAdam	Luke Hughes-Davies	32
Inverclyde Royal Hospital, Greenock	Richard Jones	Abdulla Al-Hasso	32
Queen Elizabeth Hospital, Birmingham	Christopher Poole	Daniel Rea	31

Recruiting site	Principal investigator (original)	Principal investigator (current)	Patients recruited
Ysbyty Gwynedd, Bangor	Jill Bishop	Jill Bishop	30
Royal Lancaster Infirmary	Malcolm McIllimurray	David Eaton	29
Glan Clwyd Hospital, Rhyl	Jill Bishop	Jill Bishop	28
Peterborough District Hospital	Karen McAdam	Karen McAdam	28
Eastbourne District General Hospital	Alistair Ring	Sarah Westwell	27
Northampton General Hospital	Craig Macmillan	Craig Macmillan	27
Kettering General Hospital	Roy Mathew	Salam Musa	26
University Hospital of North Durham	Wendy Taylor	Wendy Taylor	26
Withybush Hospital, Haverfordwest	Gianfilippo Bertelli	David Mark Davies	26
Raigmore Hospital, Inverness	David Whillis	Carol MacGregor	25
St Mary's Hospital, Portsmouth	Tim Gulliford	Tim Gulliford	25
Velindre Hospital, Cardiff	Peter Barrett-Lee	Peter Barrett-Lee	25
West Suffolk Hospital	Margaret Moody	Margaret Moody	25
Doncaster Royal Infirmary	Kathleen Dunn	Kathleen Dunn	24
Queen Elizabeth Hospital, Gateshead	Helen Lucraft	Daniela Lee	24
St James's University Hospital, Leeds	Timothy Perren	Timothy Perren	24
Oldchurch Hospital now called Queens, Essex	Mary Quigley	Mary Quigley	22
St Mary's Hospital, Paddington, London	Susan Cleator	Susan Cleator	22
St Mary's Hospital, Isle of Wight	Jennifer Marshall	Jennifer Marshall	21
Wrexham Maelor Hospital	Audrey Champion	Win Soe	21
Clatterbridge Centre for Oncology	Peter Clark	Susan O'Reilly	20
South Tyneside District Hospital	Goudarz Mazdai	Radha Todd	20
St John's Hospital, West Lothian	Frances Yuille	Frances Yuille	20
Yeovil District Hospital	Geoffrey Sparrow	Geoffrey Sparrow	20
Great Western Hospital, Swindon	David Cole	Anne Kendall	19
James Paget Hospital, Great Yarmouth	Adrian Harnett	Adrian Harnett	19
Cheltenham General Hospital	Kim Benstead	Kim Benstead	18
Princess Royal University Hospital, Orpington	Mark Harries	Mark Harries	18
Ayr Hospital	Lucy Scott	Lucy Scott	17
Royal Oldham Hospital	Vivek Misra	Vivek Misra	16
Basildon University Hospital	Colin Trask	Wendy Ella	15
Southport & Formby District General Hospital	Khizar Hayat	Helen Neville-Webb	15
Stepping Hill Hospital, Stockport	Abbasi Chittalia	Abbasi Chittalia	14
Ipswich Hospital	Elizabeth Sherwin	Elizabeth Sherwin	13
Weston General Hospital, Avon	Christopher Gerard Price	Thomas Wells	13
Burnley General Hospital	Martin Hogg	Martin Hogg	12
Glasgow Royal Infirmary	Peter Canney	Iain MacPherson	12
James Cook University Hospital, Middlesbrough	Alison Humphreys	Alison Humphreys	12
King George Hospital, Ilford	Mary Quigley	Mary Quigley	12
St George's Hospital, Tooting	Laura Assersohn	Laura Assersohn	12
Alexandra Hospital, Redditch	Christopher Gerard Price	Christopher Gerard Price	11
Gloucestershire Royal Hospital	Kim Benstead	Kim Benstead	11
Huddersfield Royal Infirmary	Jonathan Joffe	Jonathan Joffe	11
Belfast City Hospital	Alison Clayton	Alison Clayton	10
Royal Gwent Hospital	Christopher Gaffney	Simon Waters	10
Airedale General Hospital, Keighley	Michael Crawford	Shazza Rehman	9
Cookridge Hospital, Leeds	David Dodwell	Timothy Perren	9
Kent and Canterbury Hospital	Natasha Mithal	Natasha Mithal	9
North Hampshire Hospital now Basingstoke and North Hampshire	Sandra Tinkler	Sandra Tinkler	9
Nottingham City Hospital	Samreen Ahmed	Sarah Khan	9
Bronglais General Hospital, Aberystwyth	Sajid K Durrani	Elin Jones	8
Hereford County Hospital	Sam Guglani	Jessica Scaife	8
Kings Mill Hospital, Sutton-in-Ashfield	Sarah Khan	Sarah Khan	8
Solihull Hospital	Andrew Stockdale	Medy Tsalic	7

Recruiting site	Principal investigator (original)	Principal investigator (current)	Patients recruited
George Eliot Hospital, Nuneaton	Lydia Fresco	Susan Lupton	6
Manor Hospital, Walsall	Indrajit Fernando	Indrajit Fernando	6
York District General Hospital	David Dodwell	Andrew Proctor	6
Chesterfield and North Derbyshire Royal Hospital	Omar Din	Omar Din	5
Royal Glamorgan Hospital	Jacinta Abraham	Jacinta Abraham	5
Wycombe General Hospital	Bernadette Lavery	Ketan Shah	5
Worcestershire Royal Hospital	Joanne Bowen	Chris Price	4
Churchill Hospital, Oxford	Adrian Harris	Margaret Moody	3
King's College Hospital, Denmark Hill	Anne Sagar Rigg	Anne Sagar Rigg	3
North Middlesex University Hospital	Jacqueline Newby	Stephen Karp	3
Royal Free Hospital, London	Alison Jones	Jackie Newby	3
Sunderland Royal Hospital	Ujjal Mallick	Kathryn Wright	3
Taunton & Somerset Hospital, Musgrove Park Taunton	John Graham	John Graham	3
University Hospital Aintree	Julie O'Hagan	Julie O'Hagan	3
Barnsley District General Hospital	Caroline Bridgewater	Caroline Lee	2
City Hospital, Birmingham	Daniel Rea	Daniel Rea	2
Furness General Hospital	David Eaton	David Eaton	2
University College Hospital, London	Robert Stein	Robert Stein	2
West Middlesex University Hospital	Pippa Riddle	Pippa Riddle	2
Dewsbury and District Hospital	Sri Kumar	Sri Kumar	1
Pinderfields Hospital, Wakefield	Sri Kumar	Sri Kumar	1
Princess Royal Hospital now called Castle Hill Hospital, Hull	Amulya Chaturvedi	Amandeep Dhadda	1
Scarborough General Hospital	Michael Holmes	Amandeep Dhadda	1
Whittington Hospital, London	Alison Jones	Jayant S Vaidya	1
William Harvey Hospital, Ashford	Natasha Mithal	Natasha Mithal	1
**TOTAL**			**4391**

Protocol guidelines for dose reduction and delay

DOSE MODIFICATIONS IN RESPONSE TO TOXICITY
Every effort must be made to deliver chemotherapy on schedule except where clinically a delay is indicated. In such circumstances, delays should be kept to a minimum, and clinicians should avoid the practice of automatically deferring by one week for minor haematological reasons: patients should be re-treated as soon as is clinically appropriate. The secondary prophylactic use of GCSF in the control groups (groups 1 and 3) after an episode of significant neutropenic delay is encouraged but not mandated.

Nadir full blood tests
Routine nadir blood counts are not required: dose modifications on the basis of nadir counts are specified for patients who have blood counts measured between treatments when experiencing significant toxicity.

Recommended Dose Modifications
Recommended dose modifications are specified separately for each component of the chemotherapy regimens.

Epirubicin
Patients experiencing nadir platelet counts ≤20 x 109/L, absolute neutrophil counts (ANC) < 0.25 x 109/L, neutropenic fever, or grades 3/4 non-haematological toxicity (e.g. mucositis) should have the subsequent day 1 dose of epirubicin reduced to 80% of previous dose. Day 1 chemotherapy should be delayed until platelet counts are ≥ 100 x 109/L, ANC ≥ 1.0 x 109/L, and non-haematological toxicities have recovered to ≤ CTC grade 1. If delay of a week or more is required, doses should be reduced to 80% of original.

Cardiac toxicity
This is not anticipated at the cumulative doses of epirubicin achieved in this protocol, namely 400mg/m2 however, occasional patients with pre-existing cardiac pathology may develop problems, and clinicians should be alert to this possibility. In the event of congestive cardiac failure developing, patients should be investigated and treated as appropriate. If confirmed, epirubicin should be discontinued, and other chemotherapy may be given at the discretion of the investigator.

CMF
Patients experiencing nadir platelet counts ≤20 x 109/L, absolute neutrophil counts (ANC) < 0.25 x 109/L, neutropenic fever, or grades 3/4 non-haematological toxicity (e.g. mucositis) should have the subsequent day 1 doses of cyclophosphamide, methotrexate and 5-FU reduced to 80% of previous. Day 1 chemotherapy should be delayed until platelet counts are ≥ 100 x 109/L, ANC ≥ 1.0 x 109/L, and non-haematological toxicities (except
alopecia) have recovered to ≤ CTCAE grade 1. If a delay of a week or more is required, doses should be reduced to 80% of original. In the event that on day 8 the platelet counts are < 100 x 109/L or ANC < 1.0 x 109/L, then that dose should be omitted, and the patient re-treated with day 1 of the next cycle being on the same date as originally planned.

CAPECITABINE
Patients should be carefully monitored for toxicity, particularly during the first cycle. All patients receiving capecitabine should be reviewed on day 8 of the first cycle in order to identify the very small proportion of patients with extreme sensitivity to 5-FU as a consequence of DPD deficiency. Such patients will also have problems with CMF and would be identified by the day 8 visit of their first CMF cycle. ANY patient experiencing ≥ grade 2 diarrhoea, mucositis or hand-foot syndrome (PPE, palms of the hands or soles of the feet tingle, become numb, painful, swollen, or red) by day 8 of their first cycle should stop their capecitabine and discontinue study treatment. Toxicity due to capecitabine administration may be managed by symptomatic treatment, dose interruptions and adjustment of capecitabine dose. Once the dose has been reduced it should not be increased at a later time.

A maximum of two dose reductions are allowed per patient. capecitabine treatment interruptions are regarded as lost treatment days and the planned treatment schedule should be maintained. Patients who do not experience a recovery of the toxicity to grade < 1 after a maximum of 21 days delay (measured from the last planned treatment day) must discontinue study treatment.

The following rules will apply:

Grade 1:	Maintain dose level	
Grade 2/ 3:	1st episode	Interrupt until resolved to grade 0-1, then continue at 80% full dose
Grade 2/3:	2nd episode	Interrupt until resolved to grade 0-1, then continue at 64% full dose
Grade 2/3	3rd episode	Discontinue treatment permanently
Grade 4:	1st episode	Discontinue treatment permanently

Administration of capecitabine should be interrupted if treatment-related elevations in bilirubin of >3.0 x ULN or treatment-related elevations in hepatic aminotransferases (ALT, AST) of > 2.5 x ULN occur. Treatment should be resumed when bilirubin decreases to < 3.0 x ULN or hepatic aminotransferases decrease to < 2.5 ULN.

Neutropenia or thrombocytopenia
In case of neutropenia (neutrophils <1 x109/L) or thrombocytopenia (platelets < 100x109/L) on the planned treatment day, the treatment should be delayed until recovery of neutrophils (neutrophils >1 x 10 9/L) and/or platelets (platelets >100 x109/L) for a maximum of 21 days.

- In case of a first episode of hematological non-recovery (neutrophils <1 x109/L or platelets <100 x109/L) on the planned start day of a new cycle, the treatment should be delayed until recovery of neutrophils (neutrophils ≥1 x109/L) and/or platelets (platelets ≥100 x109/L) up to a maximum of 21 days. Subsequent doses should be given at 80%.

- In case of a second episode of hematological non-recovery on the planned start day of a new cycle, the treatment should be delayed until recovery of neutrophils and/or platelets up to a maximum of 21 days. All subsequent doses should be reduced again by 20% to 64% of the initial dose.

- Patients whose counts do not recover after a maximum treatment delay of 21 days (from the planned day of drug delivery) and patients who present a third episode of haematological non-recovery must discontinue study treatment .

It is not anticipated that capecitabine at the dose of 2,500 mg/m2 in divided doses for 14 days every 3 weeks will cause significant myelosuppression. However in the case of febrile neutropenia or life threatening infection, any further drug delivery should be discontinued for the remainder of that cycle and until full recovery. If on the planned start day of a new cycle, a patient has febrile neutropenia, the next cycle should be delayed until complete resolution. A maximum of 15 days delay is allowed. Doses in all subsequent cycles should be reduced by 20% after a first episode, and by a further 20% (36% of the starting dose) after a second episode. Patients who have febrile neutropenia after a 36% dose reduction should discontinue.

Renal function
It is well recognised that in patients with impaired renal function capecitabine can cause increased toxicity. Therefore, not withstanding the above rules for dosing in the face of toxicity, any patient whose calculated creatinine clearance drops below 50 ml/min should have the dose of capecitabine reduced to 75% of the dose of the previous cycle. If the calculated creatinine clearance falls below 40 ml/min, then they cannot be treated with capecitabine, and if there is no recovery within 21 days of a cycle being due, they must come off study treatment.

Table A2 - Additional breast cancer treatment and participation in other clinical trials by TACT2 patients

Any previous hormone replacement therapy or pre-operative endocrine therapy was to be stopped at least 4 weeks prior to chemotherapy. Additional breast cancer treatment was to be given according to current standard local practice (including the potential for these to change during the recruitment period), without influence of the patient's allocation in TACT2. All patients with ER and/or PgR positive tumours (defined as per local pathology guidelines) were to be prescribed tamoxifen or an aromatase inhibitor (AI) (or a sequence of the two) for 5 years, within 4 weeks cessation of chemotherapy. In patients for whom tamoxifen was not appropriate (e.g. history of deep venous thrombosis, contravenes agreed local protocol), an aromatase inhibitor was permitted as an alternative in accordance with local, regional or network policy. HER2 positive patients were prescribed trastuzumab for 1 year. LHRH analogue for ovarian protection in pre-menopausal women was permitted concurrently with chemotherapy and as adjuvant therapy concomitantly with tamoxifen. Radiotherapy was given after chemotherapy if required in accordance with local practice, but with clear protocol guidelines.

Patients were also permitted to enter further selected trials of adjuvant therapy provided they did not compromise the aims of this study TACT2, see table below:

	E-CMF (n=1116)		E-X (n=1105)		aE-CMF (n=1086)		aE-X (n=1084)		Total (n=4391)	
	n	%	n	%	n	%	n	%	n	%
**Radiotherapy trials**	**11**	**1.0**	**14**	**1.3**	**8**	**0.7**	**17**	**1.6**	**50**	**1.1**
SUPREMO	8	0.7	10	0.9	4	0.4	15	1.4	37	0.8
IMRT trial	1	0.1	3	0.3	2	0.2	1	0.1	7	0.2
BI-ARM	1	0.1	1	0.1	1	0.1	0	0.0	3	0.1
IMPORT LOW	0	0.0	0	0.0	1	0.1	1	0.1	2	0.0
TARGIT	1	0.1	0	0.0	0	0.0	0	0.0	1	0.0
**Endocrine therapy trials**	**9**	**0.8**	**6**	**0.5**	**9**	**0.8**	**5**	**0.5**	**29**	**0.7**
FACE	8	0.7	4	0.4	9	0.8	4	0.4	25	0.6
aTTom	0	0.0	2	0.2	0	0.0	0	0.0	2	0.0
Details not given	1	0.1	0	0.0	0	0.0	1	0.1	2	0.0
**Ovarian ablation trials**	**0**	**0.0**	**4**	**0.4**	**0**	**0.0**	**1**	**0.1**	**5**	**0.1**
TEXT	0	0.0	2	0.2	0	0.0	0	0.0	2	0.0
SOFT	0	0.0	1	0.1	0	0.0	0	0.0	1	0.0
OPTION	0	0.0	1	0.1	1	0.1	0	0.0	2	0.0
**Other biological therapy trials**	**25**	**2.2**	**25**	**2.3**	**26**	**2.4**	**24**	**2.2**	**100**	**2.3**
ALLTO	11	1.0	7	0.6	10	0.9	8	0.7	36	0.8
PERSEPHONE	5	0.4	9	0.8	7	0.6	8	0.7	29	0.7
REACT	7	0.6	7	0.6	4	0.4	4	0.4	22	0.5
O'HERA	2	0.2	2	0.2	5	0.5	4	0.4	13	0.3

Statistical considerations for non-inferiority comparison of CMF versus X

The study was designed to test non-inferiority for efficacy of X compared with CMF to give 80% probability that the lower 90% confidence limit for the difference between the X and CMF schedules would exclude 3% if the groups were truly equivalent. Assuming a 5-year TTR rate of 80% after standard E-CMF chemotherapy, a target accrual of 4400 was required

For survival-related endpoints, Kaplan-Meier curves were plotted and treatment groups compared using the log-rank test. Hazard ratios (HRs) with CIs were obtained from Cox proportional hazards regression models with HR <1 favouring X. X could be declared non-inferior to CMF if the upper 95.78% CI for the TTR HR was <1.24 (excluding a reduction in 5 year TTR from 86% to 83%). A reduction in TTR from 80% to 77% (requiring the upper 95.78% CI for the TTR HR to be <1.17) was originally hypothesised but later revised, in discussion with the Independent Data Management Committee (IDMC), due to lower than expected TTR event rates. The 95·78% confidence interval used is based on O'Brien-Fleming design, given five annual interim analyses performed, at which point inferiority of X could have been concluded if the one-sided lower 98·0% confidence bound for the TTR HR was >1.0, and non-inferiority concluded if the one sided upper 99.5% confidence bound for the TTR HR was <1·17[1]. Superiority of X compared with CMF could be tested if its non-inferiority had been established[2]. Unless stated otherwise, all analyses were unadjusted and stratified by randomisation to E or aE. Analysis of TTR non-inferiority was based on the per-protocol population, defined as all patients receiving at least one cycle of allocated protocol (CMF or X) treatment.

References

1 - Korn EL, Hunsberger S, Freidlin B, Smith MA, Abrams JS. Preliminary data release for randomized clinical trials of noninferiority: a new proposal. J Clin Oncol. 2005;23(24):5831-6.

2 - US FDA. Guidance for Industry Non-Inferiority Clinical Trials. 2010.

Table A3 - Compliance with randomised treatment

	Standard epirubicin (E) (N=2221)		Accelerated epirubicin (aE) (N=2170)		CMF (N=2202)		Capecitabine (X) (N=2189)		Total (N=4391)	
	n (%)	Cumulative proportion (%)	n (%)	Cumulative proportion (%)	n (%)	Cumulative proportion (%)	n (%)	Cumulative proportion (%)	n (%)	Cumulative proportion (%)
**Total cycles of protocol chemotherapy received**										
0	24 (1.1)	1.1	9 (0.4)	0.4	24 (1.1)	1.1	9 (0.4)	0.4	33 (0.8)	0.8
1	17 (0.8)	1.8	17 (0.8)	1.2	20 (0.9)	2	14 (0.6)	1.1	34 (0.8)	1.5
2	20 (0.9)	2.7	9 (0.4)	1.6	19 (0.9)	2.9	10 (0.5)	1.5	29 (0.7)	2.2
3	12 (0.5)	3.3	9 (0.4)	2	19 (0.9)	3.7	2 (0.1)	1.6	21 (0.5)	2.7
4	21 (0.9)	4.2	25 (1.2)	3.2	27 (1.2)	5	19 (0.9)	2.5	46 (1.0)	3.7
5	85 (3.8)	8.1	98 (4.5)	7.7	63 (2.9)	7.8	120 (5.5)	7.9	183 (4.2)	7.9
6	66 (3.0)	11	70 (3.2)	10.9	61 (2.8)	10.6	75 (3.4)	11.4	136 (3.1)	11
7	88 (4.0)	15	86 (4.0)	14.9	74 (3.4)	13.9	100 (4.6)	15.9	174 (4.0)	14.9
8	1888 (85.0)	100	1847 (85.1)	100	1895 (86.1)	100	1840 (84.1)	100	3735 (85.1)	100
**Cycles of CMF/X received**										
0	93 (4.2)	4.2	66 (3.0)	3.0	106 (4.8)	4.8	53 (2.4)	2.4	159 (3.6)	3.6
1	86 (3.9)	8.1	100 (4.6)	7.6	65 (3.0)	7.8	121 (5.5)	7.9	186 (4.2)	7.9
2	65 (2.9)	11.0	69 (3.2)	10.8	61 (2.8)	10.5	73 (3.3)	11.3	134 (3.1)	10.9
3	85 (3.8)	14.8	82 (3.8)	14.6	69 (3.1)	13.7	98 (4.5)	15.8	167 (3.8)	14.7
4	1892 (85.2)	100	1853 (85.4)	100	1901 (86.3)	100	1844 (84.2)	100	3745 (85.3)	100

Note: According to the protocol, patients not completing all four cycles of E/aE were still expected to complete all 4 cycles of CMF/X

Table A4 - Dose reductions and delays reported by cycle

Cycle		E-CMF		aE-CMF		E-X		aE-X		Total	
		n	%	n	%	n	%	n	%	n	%
1	Cycle given	1096		1082		1101		1079		4358	
	Delay only	36	*3.3*	30	*2.8*	42	*3.8*	40	*3.7*	148	*3.4*
	Reduction only	2	*0.2*	1	*0.1*	1	*0.1*	0	*0.0*	4	*0.1*
	Both delay and reduction	0	*0.0*	0	*0.0*	0	*0.0*	0	*0.0*	0	*0.0*
2	Cycle given	1086		1070		1093		1073		4322	
	Delay only	127	*11.7*	100	*9.3*	133	*12.2*	110	*10.3*	470	*10.9*
	Reduction only	14	*1.3*	13	*1.2*	16	*1.5*	11	*1.0*	54	*1.2*
	Both delay and reduction	15	*1.4*	7	*0.7*	10	*0.9*	7	*0.7*	39	*0.9*
3	Cycle given	1074		1061		1086		1070		4291	
	Delay only	109	*10.1*	76	*7.2*	102	*9.4*	70	*6.5*	357	*8.3*
	Reduction only	6	*0.6*	3	*0.3*	8	*0.7*	8	*0.7*	25	*0.6*
	Both delay and reduction	7	*0.7*	4	*0.4*	9	*0.8*	1	*0.1*	21	*0.5*
4	Cycle given	1062		1053		1081		1066		4262	
	Delay only	107	*10.1*	91	*8.6*	103	*9.5*	81	*7.6*	382	*9.0*
	Reduction only	12	*1.1*	4	*0.4*	8	*0.7*	5	*0.5*	29	*0.7*
	Both delay and reduction	4	*0.4*	8	*0.8*	7	*0.6*	13	*1.2*	32	*0.8*
5	Cycle given	1051		1044		1077		1059		4231	
	Delay only	111	*10.6*	141	*13.5*	116	*10.8*	136	*12.8*	504	*11.9*
	Reduction only	44	*4.2*	52	*5.0*	11	*1.0*	10	*0.9*	117	*2.8*
	Both delay and reduction	8	*0.8*	7	*0.7*	7	*0.6*	8	*0.8*	30	*0.7*
6	Cycle given	1024		1007		1018		997		4046	
	Delay only	122	*11.9*	131	*13.0*	49	*4.8*	55	*5.5*	357	*8.8*
	Reduction only	78	*7.6*	81	*8.0*	72	*7.1*	71	*7.1*	302	*7.5*
	Both delay and reduction	44	*4.3*	61	*6.1*	38	*3.7*	47	*4.7*	190	*4.7*
7	Cycle given	992		979		985		957		3913	
	Delay only	118	*11.9*	123	*12.6*	41	*4.2*	43	*4.5*	325	*8.3*
	Reduction only	70	*7.1*	58	*5.9*	95	*9.6*	91	*9.5*	314	*8.0*
	Both delay and reduction	28	*2.8*	47	*4.8*	94	*9.5*	108	*11.3*	277	*7.1*
8	Cycle given	956		945		936		908		3745	
	Delay only	117	*12.2*	112	*11.9*	56	*6.0*	49	*5.4*	334	*8.9*
	Reduction only	53	*5.5*	47	*5.0*	83	*8.9*	77	*8.5*	260	*6.9*
	Both delay and reduction	18	*1.9*	22	*2.3*	57	*6.1*	67	*7.4*	164	*4.4*

Percentages calculated from the number receiving the cycle

Figure A1 - Actual Fraction of the Intended Dose Density (AFIDD) across cycles 1-4

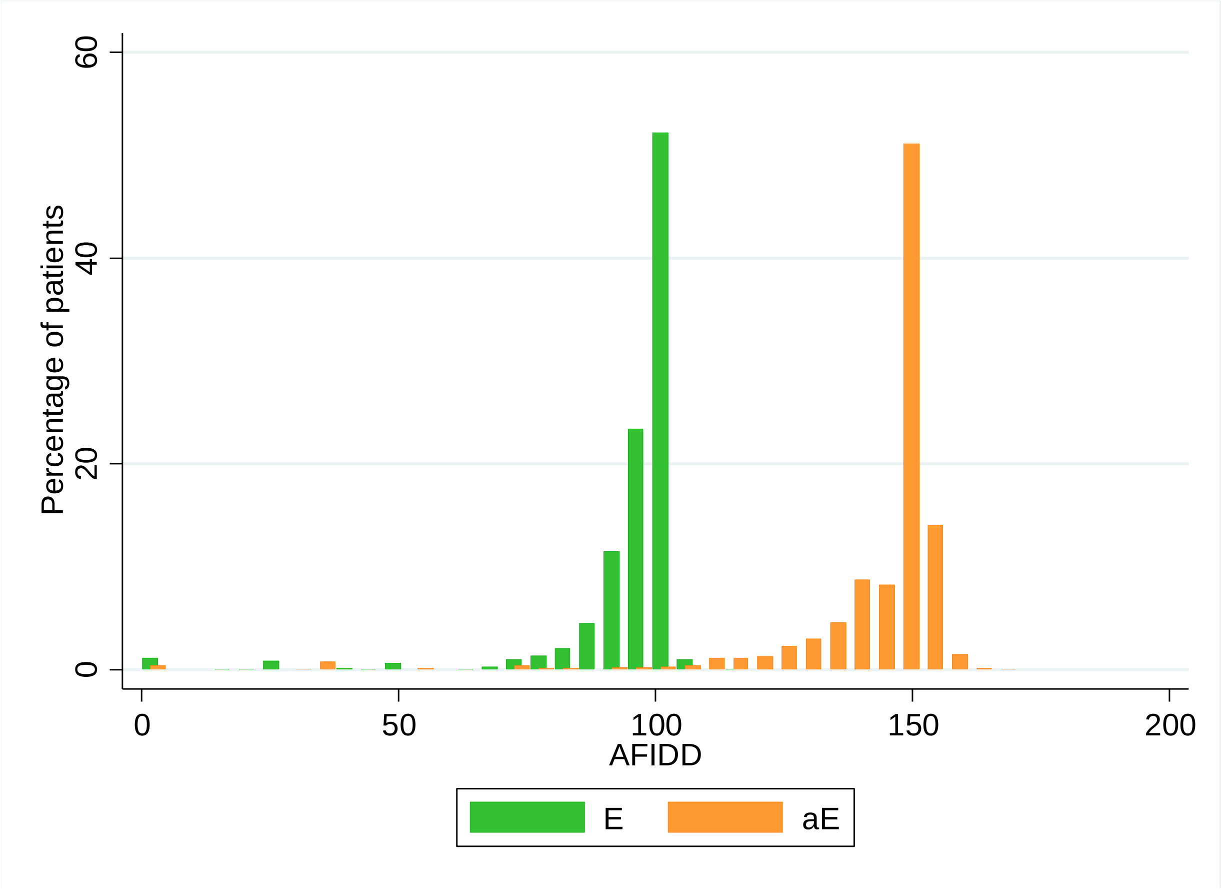

AIFIDD calculated as observed dose density divided by the protocol planned dose density for an E patient, with the mean expected to be 1.5 times higher in aE than E patients.

Table A5 - Post-epirubicin delivered therapies by E/aE

	E (N=2221)		aE (N=2170)		Total (N=4391)	
	n	%	n	%	n	%
**Cycles of CMF/Capecitabine received**						
0	93	4.2	66	3.0	159	3.6
1	86	3.9	100	4.6	186	4.2
2	65	2.9	69	3.2	134	3.1
3	85	3.8	82	3.8	167	3.8
4	1892	85.2	1853	85.4	3745	85.3
**Ovarian Ablation - ER or PGR positive, pre-menopausal patients**						
	**E (n = 669)**		**aE (n = 610)**		**Total (n = 1279)**	
Given	93	*13.9*	84	*13.8*	177	*13.8*
LHRH	69	*10.3*	61	*10*	130	*10.2*
Surgery	23	*3.4*	23	*3.8*	46	*3.6*
Radiotherapy	1	*0.1*	0	*0*	1	*0.1*
Not given	567	*84.8*	520	*85.2*	1087	*85*
Missing	9	*1.3*	6	*1*	15	*1.2*
**Planned Endocrine Therapy - ER or PgR positive patients**						
	**E (n = 1632)**		**aE (n = 1584)**		**Total (n = 3216)**	
Given	1590	*97.4*	1545	*97.5*	3135	*97.5*
Tamoxifen	848	*52*	788	*49.7*	1636	*50.9*
Tamoxifen→AI	230	*14.1*	220	*13.9*	450	*14*
AI	508	*31.1*	532	*33.6*	1040	*32.3*
AI→Tamoxifen	3	*0.2*	5	*0.3*	8	*0.2*
Not known	1	*0.1*	0	*0*	1	*0*
Not given	31	*1.9*	30	*1.9*	61	*1.9*
Missing	11	*0.7*	9	*0.6*	20	*0.6*
**Planned Endocrine Therapy - ER negative & PgR negative or not known patients**						
	**E (n = 589)**		**aE (n = 586)**		**Total (n = 1175)**	
Given	11	*1.9*	10	*1.7*	21	*1.8*
Tamoxifen	4	*0.7*	3	*0.5*	7	*0.6*
Tamoxifen→AI	2	*0.3*	3	*0.5*	5	*0.4*
AI	5	*0.8*	4	*0.7*	9	*0.8*
Not given	572	*97.1*	575	*98.1*	1147	*97.6*
Missing	6	*1*	1	*0.2*	7	*0.6*
**Other Biological Therapy - HER2 positive patients**						
	**E (n = 408)**		**aE (n = 423)**		**Total (n = 831)**	
Given	382	*93.6*	384	*90.8*	766	*92.2*
Trastuzumab	371	*90.9*	371	*87.7*	742	*89.3*

	E (N=2221)		aE (N=2170)		Total (N=4391)	
	n	%	n	%	n	%
Lapatinib	2	*0.5*	3	*0.7*	5	*0.6*
Trastuzumab and Lapatanib	9	*2.2*	10	*2.4*	19	*2.3*
Not given	22	*5.4*	38	*9*	60	*7.2*
Missing	4	*1*	1	*0.2*	5	*0.6*
**Other Biological Therapy - HER2 negative, borderline or missing patients**						
	**E (n = 1813)**		**aE (n = 1747)**		**Total (n = 3560)**	
Given	16	*0.9*	17	*1*	33	*0.9*
Trastuzumab*	6	*0.3*	10	*0.6*	16	*0.4*
Celecoxib or Placebo**	10	*0.6*	7	*0.4*	17	*0.5*
Not given	1784	*98.4*	1721	*98.5*	3505	*98.5*
Missing	13	*0.7*	9	*0.5*	22	*0.6*
**Radiotherapy - Patients with WLE as definitive surgery**						
	**E (n = 1172)**		**aE (n = 1220)**		**Total (n = 2392)**	
Given	1137	*97*	1191	*97.6*	2328	*97.3*
Breast¥	1117	*95.3*	1170	*95.9*	2287	*95.6*
Chest wall¥	22	*1.9*	19	*1.6*	41	*1.7*
SCF¥	143	*12.2*	142	*11.6*	285	*11.9*
Axilla¥	89	*7.6*	104	*8.5*	193	*8.1*
Site unknown	5	*0.4*	3	*0.2*	8	*0.3*
Not given	26	*2.2*	23	*1.9*	49	*2*
Missing	9	*0.8*	6	*0.5*	15	*0.6*
**Radiotherapy - Patients with mastectomy as definitive surgery**						
	**E (n = 1049)**		**aE (n = 949)**		**Total (n = 1998)**	
Given	660	*62.9*	583	*61.4*	1243	*62.2*
Breast¥	34	*3.2*	28	*3*	62	*3.1*
Chest wall¥	617	*58.8*	550	*58*	1167	*58.4*
SCF¥	236	*22.5*	202	*21.3*	438	*21.9*
Axilla¥	86	*8.2*	63	*6.6*	149	*7.5*
Site unknown	4	*0.4*	4	*0.4*	8	*0.4*
Not given	381	*36.3*	362	*38.1*	743	*37.2*
Missing	8	*0.8*	4	*0.4*	12	*0.6*

* - Includes 2 HER2 borderline patients (1 E, 1 aE) and no HER2 missing patients
** - Blinded treatment as part of the REACT trial
¥ - Patients receiving radiotherapy to multiple sites are included in each appropriate row
One further aE patient for whom definitive surgery is not known received radiotherapy to the breast and SCF
Of the 61 ER or PgR +ve patients who did not receive endocrine therapy, 22 were ER +ve & PgR +ve, 21 were ER –ve & PgR +ve, 16 were ER +ve & PgR –ve, and 2 were ER +ve with PgR status not known

Table A6 - Frequency of adverse events during cycles 1-4 of chemotherapy by E/aE (QOL substudy patients only)

Adverse events are included if occurring either in >10% of patients at grade ≤2, or at grade ≥3 by any patient during cycles 1-4

	E (N = 1070)								aE (N = 1045)								Trend test: p-value[a]	Grade ≥3: Fisher's exact p-value
	Grade 1 + 2		Grade 3		Grade 4		Grade 5		Grade 1 + 2		Grade 3		Grade 4		Grade 5			
	n	%	n	%	n	%	n	%	n	%	n	%	n	%	n	%		
**Pre-specified CRF adverse events**																		
Fatigue	919	85.9	52	4.9	4	0.4	0	0.0	889	85.1	62	5.9	1	0.1	0	0.0	0.16	0.45
Nausea	806	75.3	19	1.8	1	0.1	0	0.0	801	76.7	23	2.2	0	0.0	0	0.0	0.24	0.65
Vomiting	361	33.7	23	2.1	1	0.1	0	0.0	338	32.3	20	1.9	2	0.2	0	0.0	0.47	0.88
Alopecia[b]	1023	95.6	0	0.0	0	0.0	0	0.0	995	95.2	0	0.0	0	0.0	0	0.0	0.43	-
Neuropathy	132	12.3	1	0.1	0	0.0	0	0.0	113	10.8	2	0.2	0	0.0	0	0.0	0.27	0.62
Mucositis/Stomatitis	723	67.6	10	0.9	0	0.0	0	0.0	718	68.7	13	1.2	0	0.0	0	0.0	0.66	0.53
Hand-foot syndrome	36	3.4	0	0.0	0	0.0	0	0.0	80	7.7	9	0.9	0	0.0	0	0.0	<0.0001	0.0017
Skin	291	27.2	3	0.3	0	0.0	0	0.0	309	29.6	4	0.4	0	0.0	0	0.0	0.084	0.72
Thrombosis/Embolism	10	0.9	5	0.5	2	0.2	0	0.0	6	0.6	2	0.2	0	0.0	0	0.0	0.063	0.18
Superficial thrombophlebitis[b]	247	23.1	0	0.0	0	0.0	0	0.0	232	22.2	0	0.0	0	0.0	0	0.0	0.63	-
Diarrhoea	324	30.3	11	1.0	0	0.0	0	0.0	359	34.4	14	1.3	0	0.0	0	0.0	0.061	0.55
Constipation	696	65.0	7	0.7	0	0.0	0	0.0	695	66.5	4	0.4	0	0.0	0	0.0	0.46	0.55
Infection	330	30.8	34	3.2	4	0.4	0	0.0	296	28.3	33	3.2	1	0.1	0	0.0	0.18	0.72
Anaemia	264	24.7	0	0.0	0	0.0	0	0.0	421	40.3	1	0.1	0	0.0	0	0.0	<0.0001	0.49
Leucopenia	276	25.8	37	3.5	4	0.4	0	0.0	35	3.3	8	0.8	2	0.2	0	0.0	<0.0001	<0.0001
Neutropenia	318	29.7	125	11.7	50	4.7	0	0.0	46	4.4	11	1.1	7	0.7	0	0.0	<0.0001	<0.0001
Thrombocytopenia	11	1.0	1	0.1	1	0.1	0	0.0	30	2.9	0	0.0	0	0.0	0	0.0	0.10	0.50
Febrile Neutropenia	0	0.0	34	3.2	4	0.4	0	0.0	0	0.0	14	1.3	1	0.1	0	0.0	0.0019	0.002
	E (N = 1070)								aE (N = 1045)								Any grade: Fisher's exact p-value	Grade ≥3: Fisher's exact p-value
	Grade 1 + 2		Grade 3		Grade 4		Grade 5		Grade 1 + 2		Grade 3		Grade 4		Grade 5			
	n	%	n	%	n	%	n	%	n	%	n	%	n	%	n	%		
**Other MedDRA coded adverse events[c]**																		
Abdominal pain	14	1.3	0	0.0	0	0.0	0	0.0	17	1.6	3	0.3	0	0.0	0	0.0	0.30	0.12
Acute myocardial infarction	0	0.0	0	0.0	1	0.1	0	0.0	0	0.0	0	0.0	0	0.0	0	0.0	>0.99	>0.99
Alanine aminotransferase	3	0.3	0	0.0	0	0.0	0	0.0	5	0.5	1	0.1	0	0.0	0	0.0	0.34	0.49
Alanine aminotransferase increased	20	1.9	1	0.1	0	0.0	0	0.0	50	4.8	2	0.2	0	0.0	0	0.0	0.00018	0.62
Angina pectoris	0	0.0	1	0.1	0	0.0	0	0.0	0	0.0	0	0.0	0	0.0	0	0.0	>0.99	>0.99
Arthralgia	32	3.0	1	0.1	0	0.0	0	0.0	59	5.6	2	0.2	0	0.0	0	0.0	0.0022	0.62
Back pain	22	2.1	0	0.0	0	0.0	0	0.0	45	4.3	1	0.1	0	0.0	0	0.0	0.0028	0.49
Blood bilirubin increased	1	0.1	1	0.1	0	0.0	0	0.0	0	0.0	0	0.0	0	0.0	0	0.0	0.50	>0.99
Blood test	0	0.0	0	0.0	0	0.0	0	0.0	0	0.0	1	0.1	0	0.0	0	0.0	0.49	0.49
C-reactive protein increased	1	0.1	1	0.1	0	0.0	0	0.0	0	0.0	0	0.0	0	0.0	0	0.0	0.50	>0.99
Colitis	0	0.0	1	0.1	0	0.0	0	0.0	1	0.1	0	0.0	0	0.0	0	0.0	>0.99	>0.99
Conjunctival disorder	0	0.0	1	0.1	0	0.0	0	0.0	0	0.0	0	0.0	0	0.0	0	0.0	>0.99	>0.99
Cough	56	5.2	0	0.0	0	0.0	0	0.0	69	6.6	1	0.1	0	0.0	0	0.0	0.17	0.49
Dehydration	0	0.0	0	0.0	0	0.0	0	0.0	0	0.0	1	0.1	0	0.0	0	0.0	0.49	0.49
Dermatitis allergic	0	0.0	1	0.1	0	0.0	0	0.0	0	0.0	0	0.0	0	0.0	0	0.0	>0.99	>0.99
Diabetes mellitus	0	0.0	0	0.0	0	0.0	0	0.0	0	0.0	1	0.1	0	0.0	0	0.0	0.49	0.49
Dizziness	24	2.2	1	0.1	0	0.0	0	0.0	19	1.8	0	0.0	0	0.0	0	0.0	0.45	>0.99
Drug hypersensitivity	0	0.0	0	0.0	0	0.0	0	0.0	1	0.1	1	0.1	0	0.0	0	0.0	0.24	0.49
Dry mouth	44	4.1	0	0.0	0	0.0	0	0.0	58	5.6	1	0.1	0	0.0	0	0.0	0.11	0.49
Dysgeusia	147	13.7	1	0.1	0	0.0	0	0.0	165	15.8	1	0.1	0	0.0	0	0.0	0.20	>0.99
Dyspepsia	246	23.0	1	0.1	0	0.0	0	0.0	273	26.1	3	0.3	0	0.0	0	0.0	0.078	0.37
Dysphagia	5	0.5	0	0.0	0	0.0	0	0.0	4	0.4	2	0.2	0	0.0	0	0.0	0.77	0.24
Dyspnoea	30	2.8	1	0.1	0	0.0	0	0.0	34	3.3	2	0.2	2	0.2	0	0.0	0.39	0.21
Gamma-glutamyltransferase	1	0.1	0	0.0	0	0.0	0	0.0	4	0.4	1	0.1	0	0.0	0	0.0	0.12	0.49

Headache	100	9.3	5	0.5	0	0.0	0	0.0	122	11.7	2	0.2	0	0.0	0	0.0	0.14	0.45
Hot flush	44	4.1	2	0.2	0	0.0	0	0.0	40	3.8	0	0.0	0	0.0	0	0.0	0.66	0.50
Hyperglycaemia	0	0.0	0	0.0	0	0.0	0	0.0	0	0.0	1	0.1	0	0.0	0	0.0	0.49	0.49
Hypoxia	0	0.0	0	0.0	0	0.0	0	0.0	0	0.0	0	0.0	1	0.1	0	0.0	0.49	0.49
Insomnia	48	4.5	2	0.2	0	0.0	0	0.0	54	5.2	1	0.1	0	0.0	0	0.0	0.55	>0.99
Intestinal perforation	0	0.0	1	0.1	0	0.0	0	0.0	0	0.0	0	0.0	0	0.0	0	0.0	>0.99	>0.99
Leukopenia	0	0.0	1	0.1	0	0.0	0	0.0	0	0.0	0	0.0	0	0.0	0	0.0	>0.99	>0.99
Liver function test abnormal	27	2.5	3	0.3	0	0.0	0	0.0	41	3.9	3	0.3	0	0.0	0	0.0	0.10	>0.99
Lymphoedema	23	2.1	2	0.2	0	0.0	0	0.0	11	1.1	0	0.0	0	0.0	0	0.0	0.028	0.50
Malaise	4	0.4	0	0.0	0	0.0	0	0.0	3	0.3	1	0.1	0	0.0	0	0.0	>0.99	0.49
Menstruation irregular	4	0.4	1	0.1	0	0.0	0	0.0	0	0.0	0	0.0	0	0.0	0	0.0	0.062	>0.99
Musculoskeletal pain	2	0.2	0	0.0	0	0.0	0	0.0	5	0.5	3	0.3	1	0.1	0	0.0	0.036	0.059
Myalgia	35	3.3	2	0.2	0	0.0	0	0.0	44	4.2	1	0.1	0	0.0	0	0.0	0.37	>0.99
Non-cardiac chest pain	2	0.2	0	0.0	0	0.0	0	0.0	4	0.4	1	0.1	1	0.1	0	0.0	0.17	0.24
Oral candidiasis	5	0.5	1	0.1	0	0.0	0	0.0	11	1.1	0	0.0	0	0.0	0	0.0	0.23	>0.99
Oropharyngeal pain	44	4.1	1	0.1	1	0.1	0	0.0	56	5.4	1	0.1	0	0.0	0	0.0	0.23	>0.99
Orthostatic hypotension	1	0.1	0	0.0	0	0.0	0	0.0	0	0.0	1	0.1	0	0.0	0	0.0	>0.99	0.49
Pain	4	0.4	1	0.1	0	0.0	0	0.0	5	0.5	0	0.0	0	0.0	0	0.0	>0.99	>0.99
Pericardial effusion	0	0.0	0	0.0	0	0.0	0	0.0	0	0.0	1	0.1	0	0.0	0	0.0	0.49	0.49
Presyncope	1	0.1	0	0.0	0	0.0	0	0.0	1	0.1	1	0.1	0	0.0	0	0.0	0.62	0.49
Pulmonary embolism	0	0.0	0	0.0	1	0.1	0	0.0	0	0.0	0	0.0	0	0.0	0	0.0	>0.99	>0.99
Pyrexia	26	2.4	2	0.2	0	0.0	0	0.0	21	2.0	1	0.1	0	0.0	0	0.0	0.48	>0.99
Sepsis	1	0.1	0	0.0	0	0.0	0	0.0	0	0.0	3	0.3	0	0.0	0	0.0	0.37	0.12
Syncope	2	0.2	1	0.1	0	0.0	0	0.0	0	0.0	1	0.1	0	0.0	0	0.0	0.62	>0.99
Tooth abscess	1	0.1	1	0.1	0	0.0	0	0.0	3	0.3	0	0.0	0	0.0	0	0.0	0.68	>0.99
Toothache	6	0.6	0	0.0	0	0.0	0	0.0	3	0.3	1	0.1	0	0.0	0	0.0	0.75	0.49
Transaminases increased	4	0.4	0	0.0	0	0.0	0	0.0	7	0.7	2	0.2	0	0.0	0	0.0	0.17	0.24
Tremor	3	0.3	1	0.1	0	0.0	0	0.0	2	0.2	0	0.0	0	0.0	0	0.0	0.69	>0.99
Vulvovaginal candidiasis	9	0.8	1	0.1	0	0.0	0	0.0	11	1.1	2	0.2	0	0.0	0	0.0	0.53	0.62

a - Trend tests combine grade 3, 4 and 5 adverse events

b - CTC grades 3 and 4 are not applicable for alopecia or superficial thrombophlebitis

c - Free text other adverse events have been MedDRA coded. Preferred terms are included here

Table A7 - Frequency of adverse events during cycles 5-8 of chemotherapy by E/aE (QOL substudy patients only)

Adverse events are included if occurring either in >10% of patients at grade ≤2, or at grade ≥3 by any patient during cycles 5-8

	E (N = 1042)								aE (N = 1032)								Trend test: p-value[a]	Grade ≥3: Fisher's exact p-value
	Grade 1 + 2		Grade 3		Grade 4		Grade 5		Grade 1 + 2		Grade 3		Grade 4		Grade 5			
	n	%	n	%	n	%	n	%	n	%	n	%	n	%	n	%		
**Pre-specified CRF adverse events**																		
Fatigue	805	77.3	80	7.7	1	0.1	0	0.0	793	76.8	79	7.7	1	0.1	0	0.0	0.44	>0.99
Nausea	621	59.6	16	1.5	1	0.1	0	0.0	602	58.3	12	1.2	1	0.1	0	0.0	0.27	0.58
Vomiting	272	26.1	21	2.0	1	0.1	0	0.0	234	22.7	16	1.6	0	0.0	0	0.0	0.15	0.41
Alopecia[b]	920	88.3	0	0.0	0	0.0	0	0.0	945	91.6	0	0.0	0	0.0	0	0.0	0.0011	-
Neuropathy	206	19.8	3	0.3	1	0.1	0	0.0	181	17.5	1	0.1	0	0.0	0	0.0	0.093	0.37
Mucositis/Stomatitis	542	52.0	29	2.8	2	0.2	0	0.0	501	48.5	28	2.7	3	0.3	0	0.0	0.034	>0.99
Hand-foot syndrome	355	34.1	73	7.0	0	0.0	0	0.0	412	39.9	59	5.7	0	0.0	0	0.0	0.22	0.24
Skin	291	27.9	2	0.2	0	0.0	0	0.0	288	27.9	2	0.2	0	0.0	0	0.0	0.86	>0.99
Thrombosis/Embolism	17	1.6	8	0.8	5	0.5	0	0.0	13	1.3	11	1.1	4	0.4	0	0.0	0.95	0.71
Superficial thrombophlebitis[b]	137	13.1	0	0.0	0	0.0	0	0.0	169	16.4	0	0.0	0	0.0	0	0.0	0.038	-
Diarrhoea	487	46.7	51	4.9	1	0.1	0	0.0	437	42.3	56	5.4	5	0.5	0	0.0	0.15	0.38
Constipation	331	31.8	3	0.3	0	0.0	0	0.0	334	32.4	3	0.3	0	0.0	0	0.0	0.86	>0.99
Infection	233	22.4	52	5.0	2	0.2	0	0.0	227	22.0	62	6.0	4	0.4	1	0.1	0.50	0.22
Anaemia	319	30.6	12	1.2	1	0.1	0	0.0	342	33.1	21	2.0	2	0.2	0	0.0	0.016	0.10
Leucopenia	259	24.9	52	5.0	29	2.8	0	0.0	253	24.5	50	4.8	52	5.0	0	0.0	0.21	0.10
Neutropenia	217	20.8	91	8.7	79	7.6	0	0.0	211	20.4	81	7.8	93	9.0	0	0.0	0.75	0.77
Thrombocytopenia	72	6.9	6	0.6	3	0.3	0	0.0	69	6.7	8	0.8	1	0.1	0	0.0	0.99	>0.99
Febrile Neutropenia	0	0.0	41	3.9	7	0.7	0	0.0	0	0.0	62	6.0	13	1.3	0	0.0	0.010	0.012

	E (N = 1042)								aE (N = 1032)								Any grade: Fisher's exact p-value	Grade ≥3: Fisher's exact p-value
	Grade 1 + 2		Grade 3		Grade 4		Grade 5		Grade 1 + 2		Grade 3		Grade 4		Grade 5			
	n	%	n	%	n	%	n	%	n	%	n	%	n	%	n	%		
**Other MedDRA coded adverse events[c]**																		
Abdominal discomfort	7	0.7	0	0.0	0	0.0	0	0.0	5	0.5	1	0.1	0	0.0	0	0.0	>0.99	0.50
Abdominal distension	8	0.8	1	0.1	0	0.0	0	0.0	8	0.8	0	0.0	0	0.0	0	0.0	>0.99	>0.99
Abdominal pain	29	2.8	2	0.2	0	0.0	0	0.0	28	2.7	1	0.1	0	0.0	0	0.0	0.90	>0.99
Abnormal weight gain	5	0.5	1	0.1	0	0.0	0	0.0	7	0.7	0	0.0	0	0.0	0	0.0	0.79	>0.99
Acute myocardial infarction	0	0.0	0	0.0	1	0.1	0	0.0	0	0.0	0	0.0	0	0.0	0	0.0	>0.99	>0.99
Acute respiratory distress syndrome	0	0.0	0	0.0	0	0.0	0	0.0	0	0.0	0	0.0	0	0.0	1	0.1	0.50	0.50
Alanine aminotransferase increased	48	4.6	4	0.4	1	0.1	0	0.0	39	3.8	1	0.1	0	0.0	0	0.0	0.20	0.22
Amenorrhoea	0	0.0	1	0.1	0	0.0	0	0.0	0	0.0	1	0.1	1	0.1	0	0.0	0.62	0.62
Anorexia	24	2.3	1	0.1	0	0.0	0	0.0	23	2.2	2	0.2	0	0.0	0	0.0	>0.99	0.62
Anxiety	23	2.2	0	0.0	0	0.0	0	0.0	12	1.2	2	0.2	0	0.0	0	0.0	0.18	0.25
Asthenia	1	0.1	0	0.0	0	0.0	0	0.0	1	0.1	2	0.2	0	0.0	0	0.0	0.37	0.25
Back pain	20	1.9	0	0.0	0	0.0	0	0.0	28	2.7	1	0.1	0	0.0	0	0.0	0.20	0.50
Blood bilirubin increased	10	1.0	1	0.1	0	0.0	0	0.0	8	0.8	0	0.0	0	0.0	0	0.0	0.65	>0.99
Blood glucose increased	1	0.1	0	0.0	0	0.0	0	0.0	1	0.1	1	0.1	0	0.0	0	0.0	0.62	0.50
C-reactive protein abnormal	0	0.0	0	0.0	0	0.0	0	0.0	0	0.0	1	0.1	0	0.0	0	0.0	0.50	0.50
Cardiotoxicity	2	0.2	1	0.1	0	0.0	0	0.0	0	0.0	0	0.0	0	0.0	0	0.0	0.25	>0.99
Catheter related infection	1	0.1	1	0.1	0	0.0	0	0.0	0	0.0	0	0.0	0	0.0	0	0.0	0.50	>0.99
Cellulitis	0	0.0	1	0.1	0	0.0	0	0.0	3	0.3	0	0.0	0	0.0	0	0.0	0.37	>0.99
Cerebrovascular accident	0	0.0	0	0.0	0	0.0	0	0.0	0	0.0	0	0.0	0	0.0	1	0.1	0.50	0.50
Chest pain	11	1.1	1	0.1	0	0.0	0	0.0	9	0.9	2	0.2	0	0.0	0	0.0	>0.99	0.62
Cholelithiasis	0	0.0	0	0.0	0	0.0	0	0.0	0	0.0	1	0.1	0	0.0	0	0.0	0.50	0.50
Confusional state	0	0.0	1	0.1	0	0.0	0	0.0	0	0.0	0	0.0	0	0.0	0	0.0	>0.99	>0.99
Deep vein thrombosis	0	0.0	1	0.1	0	0.0	0	0.0	2	0.2	0	0.0	0	0.0	0	0.0	0.62	>0.99

Dehydration	0	0.0	0	0.0	1	0.1	0	0.0	0	0.0	0	0.0	0	0.0	0	0.0	>0.99	>0.99
Depressed mood	38	3.6	1	0.1	0	0.0	0	0.0	23	2.2	1	0.1	0	0.0	0	0.0	0.073	>0.99
Depression	17	1.6	2	0.2	0	0.0	0	0.0	13	1.3	0	0.0	0	0.0	0	0.0	0.37	0.50
Dihydropyrimidine dehydrogenase deficiency	0	0.0	0	0.0	0	0.0	0	0.0	0	0.0	0	0.0	1	0.1	0	0.0	0.50	0.50
Dizziness	31	3.0	2	0.2	0	0.0	0	0.0	28	2.7	1	0.1	0	0.0	0	0.0	0.70	>0.99
Dry mouth	80	7.7	2	0.2	0	0.0	0	0.0	60	5.8	0	0.0	0	0.0	0	0.0	0.068	0.50
Dysgeusia	115	11.0	1	0.1	0	0.0	0	0.0	113	10.9	1	0.1	0	0.0	0	0.0	>0.99	>0.99
Dyspnoea	49	4.7	2	0.2	2	0.2	0	0.0	48	4.7	6	0.6	2	0.2	0	0.0	0.77	0.26
Gastroenteritis	1	0.1	1	0.1	0	0.0	0	0.0	2	0.2	0	0.0	0	0.0	0	0.0	>0.99	>0.99
Guillain-Barre syndrome	0	0.0	0	0.0	1	0.1	0	0.0	0	0.0	0	0.0	0	0.0	0	0.0	>0.99	>0.99
Headache	44	4.2	1	0.1	0	0.0	0	0.0	44	4.3	1	0.1	0	0.0	0	0.0	>0.99	>0.99
Hot flush	68	6.5	1	0.1	1	0.1	0	0.0	81	7.8	0	0.0	0	0.0	0	0.0	0.35	0.50
Hyperglycaemia	0	0.0	2	0.2	0	0.0	0	0.0	0	0.0	2	0.2	0	0.0	0	0.0	>0.99	>0.99
Hypokalaemia	2	0.2	1	0.1	0	0.0	0	0.0	3	0.3	1	0.1	1	0.1	0	0.0	0.50	0.62
Hypomagnesaemia	0	0.0	0	0.0	0	0.0	0	0.0	1	0.1	0	0.0	1	0.1	0	0.0	0.25	0.50
Hyponatraemia	0	0.0	0	0.0	1	0.1	0	0.0	0	0.0	1	0.1	0	0.0	0	0.0	>0.99	>0.99
Hypophosphataemia	0	0.0	0	0.0	0	0.0	0	0.0	0	0.0	1	0.1	0	0.0	0	0.0	0.50	0.50
Hypotension	1	0.1	0	0.0	1	0.1	0	0.0	1	0.1	0	0.0	0	0.0	0	0.0	>0.99	>0.99
Hypoxia	0	0.0	1	0.1	0	0.0	0	0.0	0	0.0	0	0.0	1	0.1	0	0.0	>0.99	>0.99
Insomnia	41	3.9	1	0.1	0	0.0	0	0.0	25	2.4	1	0.1	0	0.0	0	0.0	0.064	>0.99
International normalised ratio increased	0	0.0	1	0.1	0	0.0	0	0.0	0	0.0	0	0.0	0	0.0	0	0.0	>0.99	>0.99
Intestinal ischaemia	0	0.0	0	0.0	1	0.1	0	0.0	0	0.0	0	0.0	1	0.1	0	0.0	>0.99	>0.99
Ischaemic stroke	0	0.0	0	0.0	0	0.0	0	0.0	0	0.0	0	0.0	0	0.0	1	0.1	0.50	0.50
Labyrinthitis	0	0.0	0	0.0	0	0.0	0	0.0	0	0.0	1	0.1	0	0.0	0	0.0	0.50	0.50
Lacrimation increased	66	6.3	1	0.1	0	0.0	0	0.0	67	6.5	0	0.0	0	0.0	0	0.0	>0.99	>0.99
Lethargy	0	0.0	0	0.0	0	0.0	0	0.0	0	0.0	1	0.1	0	0.0	0	0.0	0.50	0.50
Liver function test abnormal	34	3.3	3	0.3	0	0.0	0	0.0	31	3.0	0	0.0	0	0.0	0	0.0	0.54	0.25
Menstrual disorder	5	0.5	0	0.0	0	0.0	0	0.0	1	0.1	1	0.1	0	0.0	0	0.0	0.45	0.50
Menstruation irregular	4	0.4	1	0.1	0	0.0	0	0.0	1	0.1	2	0.2	0	0.0	0	0.0	0.73	0.62
Migraine	3	0.3	2	0.2	0	0.0	0	0.0	5	0.5	1	0.1	0	0.0	0	0.0	0.77	>0.99
Mouth ulceration	5	0.5	1	0.1	0	0.0	0	0.0	3	0.3	0	0.0	0	0.0	0	0.0	0.51	>0.99
Multi-organ failure	0	0.0	0	0.0	1	0.1	0	0.0	0	0.0	0	0.0	0	0.0	0	0.0	>0.99	>0.99
Musculoskeletal pain	7	0.7	1	0.1	0	0.0	0	0.0	5	0.5	0	0.0	0	0.0	0	0.0	0.58	>0.99
Non-cardiac chest pain	5	0.5	3	0.3	1	0.1	0	0.0	12	1.2	6	0.6	0	0.0	0	0.0	0.08	0.55
Oral candidiasis	7	0.7	1	0.1	0	0.0	0	0.0	7	0.7	0	0.0	0	0.0	0	0.0	>0.99	>0.99
Oral pain	3	0.3	0	0.0	0	0.0	0	0.0	1	0.1	0	0.0	1	0.1	0	0.0	>0.99	0.50
Pain in extremity	33	3.2	0	0.0	0	0.0	0	0.0	30	2.9	1	0.1	0	0.0	0	0.0	0.90	0.50
Pancreatitis acute	0	0.0	0	0.0	0	0.0	0	0.0	0	0.0	1	0.1	0	0.0	0	0.0	0.50	0.50
Peripheral ischaemia	0	0.0	0	0.0	1	0.1	0	0.0	0	0.0	0	0.0	0	0.0	0	0.0	>0.99	>0.99
Photophobia	1	0.1	1	0.1	0	0.0	0	0.0	0	0.0	0	0.0	0	0.0	0	0.0	0.50	>0.99
Pulmonary embolism	0	0.0	0	0.0	0	0.0	0	0.0	0	0.0	0	0.0	1	0.1	0	0.0	0.50	0.50
Quality of life decreased	0	0.0	0	0.0	0	0.0	0	0.0	0	0.0	1	0.1	0	0.0	0	0.0	0.50	0.50
Renal failure	0	0.0	1	0.1	0	0.0	0	0.0	0	0.0	0	0.0	0	0.0	0	0.0	>0.99	>0.99
Sinus tachycardia	1	0.1	0	0.0	0	0.0	0	0.0	0	0.0	0	0.0	1	0.1	0	0.0	>0.99	0.50
Skin ulcer	2	0.2	1	0.1	0	0.0	0	0.0	0	0.0	0	0.0	0	0.0	0	0.0	0.25	>0.99
Stomatitis	7	0.7	0	0.0	0	0.0	0	0.0	3	0.3	1	0.1	0	0.0	0	0.0	0.55	0.50
Syncope	1	0.1	1	0.1	0	0.0	0	0.0	0	0.0	1	0.1	0	0.0	0	0.0	>0.99	>0.99
Tachycardia	2	0.2	0	0.0	0	0.0	0	0.0	1	0.1	1	0.1	0	0.0	0	0.0	>0.99	0.50
Vein pain	3	0.3	1	0.1	0	0.0	0	0.0	5	0.5	0	0.0	0	0.0	0	0.0	0.75	>0.99
Vertigo	1	0.1	1	0.1	0	0.0	0	0.0	0	0.0	0	0.0	0	0.0	0	0.0	0.50	>0.99
Vulvovaginal candidiasis	6	0.6	1	0.1	0	0.0	0	0.0	9	0.9	0	0.0	0	0.0	0	0.0	0.63	>0.99

a - Trend tests combine grade 3, 4 and 5 adverse events

b - CTC grades 3 and 4 are not applicable for alopecia or superficial thrombophlebitis

c - Free text other adverse events have been MedDRA coded. Preferred terms are included here

Table A8 - Frequency of adverse events during cycles 1-8 of chemotherapy by E/aE (QOL substudy patients only)

Adverse events are included if occurring either in >10% of patients at grade ≤2, or at grade ≥3 by any patient during cycles 1-8

	E (N = 1070)								aE (N = 1045)								Trend test: p-value[a]	Grade ≥3: Fisher's exact p-value
	Grade 1 + 2		Grade 3		Grade 4		Grade 5		Grade 1 + 2		Grade 3		Grade 4		Grade 5			
	n	%	n	%	n	%	n	%	n	%	n	%	n	%	n	%		
**Pre-specified CRF adverse events**																		
Fatigue	905	84.6	113	10.6	5	0.5	0	0.0	882	84.4	114	10.9	2	0.2	0	0.0	0.84	>0.99
Nausea	876	81.9	34	3.2	2	0.2	0	0.0	866	82.9	32	3.1	1	0.1	0	0.0	0.96	0.81
Vomiting	490	45.8	43	4.0	2	0.2	0	0.0	431	41.2	34	3.3	2	0.2	0	0.0	0.060	0.37
Alopecia[b]	1037	96.9	0	0.0	0	0.0	0	0.0	1014	97.0	0	0.0	0	0.0	0	0.0	0.98	-
Neuropathy	273	25.5	4	0.4	1	0.1	0	0.0	241	23.1	3	0.3	0	0.0	0	0.0	0.095	0.73
Mucositis/Stomatitis	831	77.7	36	3.4	2	0.2	0	0.0	799	76.5	40	3.8	3	0.3	0	0.0	0.38	0.57
Hand-foot syndrome	370	34.6	73	6.8	0	0.0	0	0.0	438	41.9	66	6.3	0	0.0	0	0.0	0.022	0.66
Skin	446	41.7	5	0.5	0	0.0	0	0.0	455	43.5	6	0.6	0	0.0	0	0.0	0.16	0.77
Thrombosis/Embolism	21	2.0	12	1.1	7	0.7	0	0.0	17	1.6	13	1.2	4	0.4	0	0.0	0.58	0.87
Superficial thrombophlebitis[b]	281	26.3	0	0.0	0	0.0	0	0.0	285	27.3	0	0.0	0	0.0	0	0.0	0.60	-
Diarrhoea	580	54.2	61	5.7	1	0.1	0	0.0	558	53.4	69	6.6	5	0.5	0	0.0	0.99	0.25
Constipation	747	69.8	10	0.9	0	0.0	0	0.0	739	70.7	7	0.7	0	0.0	0	0.0	0.89	0.63
Infection	434	40.6	84	7.9	6	0.6	0	0.0	390	37.3	95	9.1	5	0.5	1	0.1	0.58	0.33
Anaemia	399	37.3	12	1.1	1	0.1	0	0.0	502	48.0	22	2.1	2	0.2	0	0.0	<0.0001	0.067
Leucopenia	363	33.9	83	7.8	33	3.1	0	0.0	264	25.3	55	5.3	54	5.2	0	0.0	0.001	0.78
Neutropenia	337	31.5	165	15.4	124	11.6	0	0.0	222	21.2	88	8.4	100	9.6	0	0.0	<0.0001	<0.0001
Thrombocytopenia	79	7.4	7	0.7	4	0.4	0	0.0	93	8.9	8	0.8	1	0.1	0	0.0	0.51	0.82
Febrile Neutropenia	0	0.0	72	6.7	11	1.0	0	0.0	0	0.0	76	7.3	14	1.3	0	0.0	0.47	0.48
	**E (N = 1070)**								**aE (N = 1045)**								**Any grade: Fisher's exact p-value**	**Grade ≥3: Fisher's exact p-value**
	**Grade 1 + 2**		**Grade 3**		**Grade 4**		**Grade 5**		**Grade 1 + 2**		**Grade 3**		**Grade 4**		**Grade 5**			
	**n**	**%**	**n**	**%**	**n**	**%**	**n**	**%**	**n**	**%**	**n**	**%**	**n**	**%**	**n**	**%**		
**Other MedDRA coded adverse events[c]**																		
Abdominal discomfort	10	0.9	0	0.0	0	0.0	0	0.0	6	0.6	1	0.1	0	0.0	0	0.0	0.63	0.49
Abdominal distension	19	1.8	1	0.1	0	0.0	0	0.0	16	1.5	0	0.0	0	0.0	0	0.0	0.62	>0.99
Abdominal pain	39	3.6	2	0.2	0	0.0	0	0.0	44	4.2	4	0.4	0	0.0	0	0.0	0.39	0.45
Abnormal weight gain	9	0.8	1	0.1	0	0.0	0	0.0	8	0.8	0	0.0	0	0.0	0	0.0	0.81	>0.99
Acute myocardial infarction	0	0.0	0	0.0	2	0.2	0	0.0	0	0.0	0	0.0	0	0.0	0	0.0	0.50	0.50
Acute respiratory distress syndrome	0	0.0	0	0.0	0	0.0	0	0.0	0	0.0	0	0.0	0	0.0	1	0.1	0.49	0.49
Alanine aminotransferase	11	1.0	0	0.0	0	0.0	0	0.0	10	1.0	1	0.1	0	0.0	0	0.0	>0.99	0.49
Alanine aminotransferase increased	53	5.0	5	0.5	1	0.1	0	0.0	65	6.2	3	0.3	0	0.0	0	0.0	0.36	0.51
Amenorrhoea	0	0.0	1	0.1	0	0.0	0	0.0	0	0.0	1	0.1	1	0.1	0	0.0	0.62	0.62
Angina pectoris	1	0.1	1	0.1	0	0.0	0	0.0	1	0.1	0	0.0	0	0.0	0	0.0	>0.99	>0.99
Anorexia	34	3.2	1	0.1	0	0.0	0	0.0	40	3.8	2	0.2	0	0.0	0	0.0	0.42	0.62
Anxiety	30	2.8	0	0.0	0	0.0	0	0.0	21	2.0	2	0.2	0	0.0	0	0.0	0.41	0.24
Arthralgia	65	6.1	1	0.1	0	0.0	0	0.0	98	9.4	2	0.2	0	0.0	0	0.0	0.0045	0.62
Asthenia	1	0.1	0	0.0	0	0.0	0	0.0	4	0.4	2	0.2	0	0.0	0	0.0	0.067	0.24
Back pain	36	3.4	0	0.0	0	0.0	0	0.0	66	6.3	2	0.2	0	0.0	0	0.0	0.00086	0.24
Blood bilirubin increased	11	1.0	2	0.2	0	0.0	0	0.0	8	0.8	0	0.0	0	0.0	0	0.0	0.38	0.50
Blood glucose increased	1	0.1	0	0.0	0	0.0	0	0.0	1	0.1	1	0.1	0	0.0	0	0.0	0.62	0.49
Blood test	0	0.0	0	0.0	0	0.0	0	0.0	0	0.0	1	0.1	0	0.0	0	0.0	0.49	0.49
C-reactive protein abnormal	0	0.0	0	0.0	0	0.0	0	0.0	1	0.1	1	0.1	0	0.0	0	0.0	0.24	0.49
C-reactive protein increased	1	0.1	1	0.1	0	0.0	0	0.0	0	0.0	0	0.0	0	0.0	0	0.0	0.50	>0.99
Cardiotoxicity	2	0.2	1	0.1	0	0.0	0	0.0	0	0.0	0	0.0	0	0.0	0	0.0	0.25	>0.99
Catheter related infection	1	0.1	1	0.1	0	0.0	0	0.0	0	0.0	0	0.0	0	0.0	0	0.0	0.50	>0.99

Cellulitis	1	0.1	1	0.1	0	0.0	0	0.0	5	0.5	0	0.0	0	0.0	0	0.0	0.28	>0.99
Cerebrovascular accident	0	0.0	0	0.0	0	0.0	0	0.0	0	0.0	0	0.0	0	0.0	1	0.1	0.49	0.49
Chest pain	11	1.0	1	0.1	0	0.0	0	0.0	13	1.2	2	0.2	0	0.0	0	0.0	0.57	0.62
Cholelithiasis	0	0.0	0	0.0	0	0.0	0	0.0	0	0.0	1	0.1	0	0.0	0	0.0	0.49	0.49
Colitis	0	0.0	1	0.1	0	0.0	0	0.0	2	0.2	0	0.0	0	0.0	0	0.0	0.62	>0.99
Confusional state	0	0.0	1	0.1	0	0.0	0	0.0	2	0.2	0	0.0	0	0.0	0	0.0	0.62	>0.99
Conjunctival disorder	0	0.0	1	0.1	0	0.0	0	0.0	0	0.0	0	0.0	0	0.0	0	0.0	>0.99	>0.99
Cough	84	7.9	0	0.0	0	0.0	0	0.0	100	9.6	1	0.1	0	0.0	0	0.0	0.14	0.49
Deep vein thrombosis	0	0.0	1	0.1	0	0.0	0	0.0	2	0.2	0	0.0	0	0.0	0	0.0	0.62	>0.99
Dehydration	0	0.0	0	0.0	1	0.1	0	0.0	0	0.0	1	0.1	0	0.0	0	0.0	>0.99	>0.99
Depressed mood	61	5.7	1	0.1	0	0.0	0	0.0	52	5.0	1	0.1	0	0.0	0	0.0	0.50	>0.99
Depression	29	2.7	2	0.2	0	0.0	0	0.0	22	2.1	0	0.0	0	0.0	0	0.0	0.27	0.50
Dermatitis allergic	0	0.0	1	0.1	0	0.0	0	0.0	1	0.1	0	0.0	0	0.0	0	0.0	>0.99	>0.99
Diabetes mellitus	0	0.0	0	0.0	0	0.0	0	0.0	1	0.1	1	0.1	0	0.0	0	0.0	0.24	0.49
Dihydropyrimidine dehydrogenase deficiency	0	0.0	0	0.0	0	0.0	0	0.0	0	0.0	0	0.0	1	0.1	0	0.0	0.49	0.49
Dizziness	50	4.7	3	0.3	0	0.0	0	0.0	41	3.9	1	0.1	0	0.0	0	0.0	0.34	0.62
Drug hypersensitivity	0	0.0	0	0.0	0	0.0	0	0.0	1	0.1	1	0.1	0	0.0	0	0.0	0.24	0.49
Dry mouth	100	9.3	2	0.2	0	0.0	0	0.0	99	9.5	1	0.1	0	0.0	0	0.0	>0.99	>0.99
Dysgeusia	197	18.4	2	0.2	0	0.0	0	0.0	214	20.5	2	0.2	0	0.0	0	0.0	0.25	>0.99
Dyspepsia	296	27.7	1	0.1	0	0.0	0	0.0	298	28.5	3	0.3	0	0.0	0	0.0	0.60	0.37
Dysphagia	6	0.6	0	0.0	0	0.0	0	0.0	5	0.5	2	0.2	0	0.0	0	0.0	0.79	0.24
Dyspnoea	68	6.4	3	0.3	2	0.2	0	0.0	69	6.6	7	0.7	4	0.4	0	0.0	0.50	0.14
Gamma-glutamyltransferase	2	0.2	0	0.0	0	0.0	0	0.0	4	0.4	1	0.1	0	0.0	0	0.0	0.28	0.49
Gastroenteritis	2	0.2	1	0.1	0	0.0	0	0.0	2	0.2	0	0.0	0	0.0	0	0.0	>0.99	>0.99
Guillain-Barre syndrome	0	0.0	0	0.0	1	0.1	0	0.0	0	0.0	0	0.0	0	0.0	0	0.0	>0.99	>0.99
Headache	128	12.0	6	0.6	0	0.0	0	0.0	154	14.7	3	0.3	0	0.0	0	0.0	0.10	0.51
Hot flush	95	8.9	3	0.3	1	0.1	0	0.0	105	10.0	0	0.0	0	0.0	0	0.0	0.56	0.12
Hyperglycaemia	0	0.0	2	0.2	0	0.0	0	0.0	0	0.0	3	0.3	0	0.0	0	0.0	0.68	0.68
Hypokalaemia	2	0.2	1	0.1	0	0.0	0	0.0	4	0.4	1	0.1	1	0.1	0	0.0	0.34	0.62
Hypomagnesaemia	0	0.0	0	0.0	0	0.0	0	0.0	1	0.1	0	0.0	1	0.1	0	0.0	0.24	0.49
Hyponatraemia	0	0.0	0	0.0	1	0.1	0	0.0	0	0.0	1	0.1	0	0.0	0	0.0	>0.99	>0.99
Hypophosphataemia	0	0.0	0	0.0	0	0.0	0	0.0	0	0.0	1	0.1	0	0.0	0	0.0	0.49	0.49
Hypotension	2	0.2	0	0.0	1	0.1	0	0.0	3	0.3	0	0.0	0	0.0	0	0.0	>0.99	>0.99
Hypoxia	0	0.0	1	0.1	0	0.0	0	0.0	0	0.0	0	0.0	2	0.2	0	0.0	0.62	0.62
Insomnia	75	7.0	3	0.3	0	0.0	0	0.0	71	6.8	2	0.2	0	0.0	0	0.0	0.80	>0.99
International normalised ratio increased	0	0.0	1	0.1	0	0.0	0	0.0	0	0.0	0	0.0	0	0.0	0	0.0	>0.99	>0.99
Intestinal ischaemia	0	0.0	0	0.0	1	0.1	0	0.0	0	0.0	0	0.0	1	0.1	0	0.0	>0.99	>0.99
Intestinal perforation	0	0.0	1	0.1	0	0.0	0	0.0	0	0.0	0	0.0	0	0.0	0	0.0	>0.99	>0.99
Ischaemic stroke	0	0.0	0	0.0	0	0.0	0	0.0	0	0.0	0	0.0	0	0.0	1	0.1	0.49	0.49
Labyrinthitis	0	0.0	0	0.0	0	0.0	0	0.0	0	0.0	1	0.1	0	0.0	0	0.0	0.49	0.49
Lacrimation increased	112	10.5	1	0.1	0	0.0	0	0.0	130	12.4	0	0.0	0	0.0	0	0.0	0.20	>0.99
Lethargy	0	0.0	0	0.0	0	0.0	0	0.0	0	0.0	1	0.1	0	0.0	0	0.0	0.49	0.49
Leukopenia	1	0.1	1	0.1	0	0.0	0	0.0	0	0.0	0	0.0	0	0.0	0	0.0	0.50	>0.99
Liver function test abnormal	46	4.3	5	0.5	0	0.0	0	0.0	61	5.8	3	0.3	0	0.0	0	0.0	0.18	0.73
Lymphoedema	42	3.9	2	0.2	0	0.0	0	0.0	31	3.0	0	0.0	0	0.0	0	0.0	0.16	0.50
Malaise	4	0.4	0	0.0	0	0.0	0	0.0	5	0.5	1	0.1	0	0.0	0	0.0	0.54	0.49
Menstrual disorder	5	0.5	0	0.0	0	0.0	0	0.0	3	0.3	1	0.1	0	0.0	0	0.0	>0.99	0.49
Menstruation irregular	7	0.7	2	0.2	0	0.0	0	0.0	1	0.1	2	0.2	0	0.0	0	0.0	0.15	>0.99
Migraine	8	0.7	2	0.2	0	0.0	0	0.0	14	1.3	1	0.1	0	0.0	0	0.0	0.32	>0.99
Mouth ulceration	10	0.9	1	0.1	0	0.0	0	0.0	4	0.4	0	0.0	0	0.0	0	0.0	0.12	>0.99
Multi-organ failure	0	0.0	0	0.0	1	0.1	0	0.0	0	0.0	0	0.0	0	0.0	0	0.0	>0.99	>0.99
Musculoskeletal pain	9	0.8	1	0.1	0	0.0	0	0.0	10	1.0	3	0.3	1	0.1	0	0.0	0.42	0.21
Myalgia	58	5.4	2	0.2	0	0.0	0	0.0	65	6.2	1	0.1	0	0.0	0	0.0	0.52	>0.99
Non-cardiac chest pain	7	0.7	3	0.3	1	0.1	0	0.0	15	1.4	7	0.7	1	0.1	0	0.0	0.038	0.26
Oral candidiasis	10	0.9	2	0.2	0	0.0	0	0.0	17	1.6	0	0.0	0	0.0	0	0.0	0.35	0.50
Oral pain	8	0.7	0	0.0	0	0.0	0	0.0	2	0.2	0	0.0	1	0.1	0	0.0	0.23	0.49
Oropharyngeal pain	61	5.7	1	0.1	1	0.1	0	0.0	72	6.9	1	0.1	0	0.0	0	0.0	0.33	>0.99
Orthostatic hypotension	2	0.2	0	0.0	0	0.0	0	0.0	1	0.1	1	0.1	0	0.0	0	0.0	>0.99	0.49
Pain	9	0.8	1	0.1	0	0.0	0	0.0	7	0.7	0	0.0	0	0.0	0	0.0	0.63	>0.99

Pain in extremity	67	6.3	0	0.0	0	0.0	0	0.0	67	6.4	1	0.1	0	0.0	0	0.0	0.86	0.49
Pancreatitis acute	0	0.0	0	0.0	0	0.0	0	0.0	0	0.0	1	0.1	0	0.0	0	0.0	0.49	0.49
Pericardial effusion	0	0.0	0	0.0	0	0.0	0	0.0	0	0.0	1	0.1	0	0.0	0	0.0	0.49	0.49
Peripheral ischaemia	0	0.0	0	0.0	1	0.1	0	0.0	0	0.0	0	0.0	0	0.0	0	0.0	>0.99	>0.99
Photophobia	1	0.1	1	0.1	0	0.0	0	0.0	0	0.0	0	0.0	0	0.0	0	0.0	0.50	>0.99
Presyncope	1	0.1	0	0.0	0	0.0	0	0.0	1	0.1	1	0.1	0	0.0	0	0.0	0.62	0.49
Pulmonary embolism	0	0.0	0	0.0	1	0.1	0	0.0	0	0.0	0	0.0	1	0.1	0	0.0	>0.99	>0.99
Pyrexia	43	4.0	2	0.2	0	0.0	0	0.0	49	4.7	1	0.1	0	0.0	0	0.0	0.53	>0.99
Quality of life decreased	0	0.0	0	0.0	0	0.0	0	0.0	0	0.0	1	0.1	0	0.0	0	0.0	0.49	0.49
Renal failure	0	0.0	1	0.1	0	0.0	0	0.0	0	0.0	0	0.0	0	0.0	0	0.0	>0.99	>0.99
Sepsis	1	0.1	0	0.0	0	0.0	0	0.0	0	0.0	3	0.3	0	0.0	0	0.0	0.37	0.12
Sinus tachycardia	1	0.1	0	0.0	0	0.0	0	0.0	0	0.0	0	0.0	1	0.1	0	0.0	>0.99	0.49
Skin ulcer	3	0.3	1	0.1	0	0.0	0	0.0	1	0.1	0	0.0	0	0.0	0	0.0	0.37	>0.99
Stomatitis	17	1.6	0	0.0	0	0.0	0	0.0	8	0.8	1	0.1	0	0.0	0	0.0	0.17	0.49
Syncope	3	0.3	2	0.2	0	0.0	0	0.0	0	0.0	2	0.2	0	0.0	0	0.0	0.45	>0.99
Tachycardia	3	0.3	0	0.0	0	0.0	0	0.0	1	0.1	1	0.1	0	0.0	0	0.0	>0.99	0.49
Tooth abscess	1	0.1	1	0.1	0	0.0	0	0.0	4	0.4	0	0.0	0	0.0	0	0.0	0.45	>0.99
Toothache	12	1.1	0	0.0	0	0.0	0	0.0	9	0.9	1	0.1	0	0.0	0	0.0	0.83	0.49
Transaminases increased	8	0.7	0	0.0	0	0.0	0	0.0	9	0.9	2	0.2	0	0.0	0	0.0	0.50	0.24
Tremor	3	0.3	1	0.1	0	0.0	0	0.0	4	0.4	0	0.0	0	0.0	0	0.0	>0.99	>0.99
Vein pain	20	1.9	1	0.1	0	0.0	0	0.0	13	1.2	0	0.0	0	0.0	0	0.0	0.23	>0.99
Vertigo	3	0.3	1	0.1	0	0.0	0	0.0	1	0.1	0	0.0	0	0.0	0	0.0	0.37	>0.99
Vulvovaginal candidiasis	15	1.4	2	0.2	0	0.0	0	0.0	17	1.6	2	0.2	0	0.0	0	0.0	0.74	>0.99

a - Trend tests combine grade 3, 4 and 5 adverse events

b - CTC grades 3 and 4 are not applicable for alopecia or superficial thrombophlebitis

c - Free text other adverse events have been MedDRA coded. Preferred terms are included here

Table A9 - Hospital admissions during cycles 1-4 of chemotherapy by E/aE

Coded reason for stay	Total number of inpatient stays[a]	E			aE			Total		
		Patients receiving cycle	Patients with at least one inpatient stay	%	Patients receiving cycle	Patients with at least one inpatient stay	%	Patients receiving cycle	Patients with at least one inpatient stay	%
**Cycle 1**										
Neutropenic sepsis	24	2197	21	1.0	2161	3	0.1	4358	24	0.6
Febrile neutropenia	25		18	0.8		7	0.3		25	0.6
Neutropenic infection	1		1	0.0		0	0.0		1	0.0
**Any of the above**	**50**		**39**	**1.8**		**10**	**0.5**		**49**	**1.1**
**Any reason**	**321**		**133**	**6.1**		**101**	**4.7**		**234**	**5.4**
**Cycle 2**										
Neutropenic sepsis	6	2179	4	0.2	2143	2	0.1	4322	6	0.1
Febrile neutropenia	10		8	0.4		1	0.0		9	0.2
Neutropenic infection	0		0	0.0		0	0.0		0	0.0
**Any of the above**	**16**		**12**	**0.6**		**3**	**0.1**		**15**	**0.3**
**Any reason**	**174**		**65**	**3.0**		**59**	**2.8**		**124**	**2.9**
**Cycle 3**										
Neutropenic sepsis	5	2160	4	0.2	2131	1	0.0	4291	5	0.1
Febrile neutropenia	6		5	0.2		0	0.0		5	0.1
Neutropenic infection	1		1	0.0		0	0.0		1	0.0
**Any of the above**	**12**		**10**	**0.5**		**1**	**0.0**		**11**	**0.3**
**Any reason**	**176**		**62**	**2.9**		**54**	**2.5**		**116**	**2.7**
**Cycle 4**										
Neutropenic sepsis	9	2143	5	0.2	2119	4	0.2	4262	9	0.2
Febrile neutropenia	11		10	0.5		1	0.0		11	0.3
Neutropenic infection	1		1	0.0		0	0.0		1	0.0
**Any of the above**	**21**		**16**	**0.7**		**5**	**0.2**		**21**	**0.5**
**Any reason**	**176**		**63**	**2.9**		**70**	**3.3**		**133**	**3.1**
**Cycles 1-4**										
Neutropenic sepsis	44	2197	34	1.5	2161	10	0.5	4358	44	1.0
Febrile neutropenia	52		40	1.8		9	0.4		49	1.1
Neutropenic infection	3		3	0.1		0	0.0		3	0.1
**Any of the above**	**99**		**76**	**3.5**		**19**	**0.9**		**95**	**2.2**
**Any reason**	**857**		**283**	**12.9**		**246**	**11.4**		**529**	**12.1**

a - Includes multiple hospitalisations per patient

Reasons for inpatient stays during chemotherapy have been MedDRA coded. Proportions of patients where the coded reason for hospitalisation are "Neutropenic sepsis", "Febrile neutropenia" and "Neutropenic infection" are reported here, along with hospitalisations for any reason. For analyses of individual cycles, one admission per patient per cycle included. For overall analysis of cycles 1-4 combined one admission per patient across all cycles included

Table A10 - Frequency of adverse events during cycles 1-4 of chemotherapy by CMF/X (QOL substudy patients only)

Adverse events are included if occurring either in >10% of patients at grade ≤2, or at grade ≥3 by any patient during cycles 1-4

	CMF (N = 1053)								X (N = 1062)								Trend test: p-value[a]	Grade ≥3: Fisher's exact p-value
	Grade 1 + 2		Grade 3		Grade 4		Grade 5		Grade 1 + 2		Grade 3		Grade 4		Grade 5			
	n	%	n	%	n	%	n	%	n	%	n	%	n	%	n	%		
**Pre-specified CRF adverse events**																		
Fatigue	892	84.7	58	5.5	2	0.2	0	0.0	916	86.3	56	5.3	3	0.3	0	0.0	0.75	0.92
Nausea	800	76.0	24	2.3	0	0.0	0	0.0	807	76.0	18	1.7	1	0.1	0	0.0	0.29	0.45
Vomiting	336	31.9	22	2.1	3	0.3	0	0.0	363	34.2	21	2.0	0	0.0	0	0.0	0.83	0.55
Alopecia[b]	1009	95.8	0	0.0	0	0.0	0	0.0	1009	95.0	0	0.0	0	0.0	0	0.0	0.12	-
Neuropathy	125	11.9	2	0.2	0	0.0	0	0.0	120	11.3	1	0.1	0	0.0	0	0.0	0.37	0.62
Mucositis/Stomatitis	705	67.0	14	1.3	0	0.0	0	0.0	736	69.3	9	0.8	0	0.0	0	0.0	0.82	0.30
Hand-foot syndrome	60	5.7	4	0.4	0	0.0	0	0.0	56	5.3	5	0.5	0	0.0	0	0.0	0.88	>0.99
Skin	303	28.8	3	0.3	0	0.0	0	0.0	297	28.0	4	0.4	0	0.0	0	0.0	0.63	>0.99
Thrombosis/Embolism	8	0.8	2	0.2	0	0.0	0	0.0	8	0.8	5	0.5	2	0.2	0	0.0	0.22	0.18
Superficial thrombophlebitis[b]	229	21.7	0	0.0	0	0.0	0	0.0	250	23.5	0	0.0	0	0.0	0	0.0	0.32	-
Diarrhoea	329	31.2	10	0.9	0	0.0	0	0.0	354	33.3	15	1.4	0	0.0	0	0.0	0.095	0.42
Constipation	706	67.0	4	0.4	0	0.0	0	0.0	685	64.5	7	0.7	0	0.0	0	0.0	0.79	0.55
Infection	319	30.3	33	3.1	4	0.4	0	0.0	307	28.9	34	3.2	1	0.1	0	0.0	0.44	0.81
Anaemia	354	33.6	1	0.1	0	0.0	0	0.0	331	31.2	0	0.0	0	0.0	0	0.0	0.14	0.50
Leucopenia	165	15.7	28	2.7	4	0.4	0	0.0	146	13.7	17	1.6	2	0.2	0	0.0	0.088	0.066
Neutropenia	194	18.4	64	6.1	33	3.1	0	0.0	170	16.0	72	6.8	24	2.3	0	0.0	0.39	0.94
Thrombocytopenia	19	1.8	1	0.1	1	0.1	0	0.0	22	2.1	0	0.0	0	0.0	0	0.0	0.88	0.25
Febrile Neutropenia	0	0.0	22	2.1	2	0.2	0	0.0	0	0.0	26	2.4	3	0.3	0	0.0	0.51	0.58

	CMF (N = 1053)								X (N = 1062)								Any grade: Fisher's exact p-value	Grade ≥3: Fisher's exact p-value
	Grade 1 + 2		Grade 3		Grade 4		Grade 5		Grade 1 + 2		Grade 3		Grade 4		Grade 5			
	n	%	n	%	n	%	n	%	n	%	n	%	n	%	n	%		
**Other MedDRA coded adverse events[c]**																		
Abdominal pain	16	1.5	1	0.1	0	0.0	0	0.0	15	1.4	2	0.2	0	0.0	0	0.0	>0.99	>0.99
Acute myocardial infarction	0	0.0	0	0.0	1	0.1	0	0.0	0	0.0	0	0.0	0	0.0	0	0.0	0.50	0.50
Alanine aminotransferase	4	0.4	0	0.0	0	0.0	0	0.0	4	0.4	1	0.1	0	0.0	0	0.0	>0.99	>0.99
Alanine aminotransferase increased	31	2.9	0	0.0	0	0.0	0	0.0	39	3.7	3	0.3	0	0.0	0	0.0	0.23	0.25
Angina pectoris	0	0.0	0	0.0	0	0.0	0	0.0	0	0.0	1	0.1	0	0.0	0	0.0	>0.99	>0.99
Arthralgia	42	4.0	1	0.1	0	0.0	0	0.0	49	4.6	2	0.2	0	0.0	0	0.0	0.46	>0.99
Back pain	33	3.1	0	0.0	0	0.0	0	0.0	34	3.2	1	0.1	0	0.0	0	0.0	0.90	>0.99
Blood bilirubin increased	0	0.0	0	0.0	0	0.0	0	0.0	1	0.1	1	0.1	0	0.0	0	0.0	0.50	>0.99
Blood test	0	0.0	1	0.1	0	0.0	0	0.0	0	0.0	0	0.0	0	0.0	0	0.0	0.50	0.50
C-reactive protein increased	0	0.0	1	0.1	0	0.0	0	0.0	1	0.1	0	0.0	0	0.0	0	0.0	>0.99	0.50
Colitis	1	0.1	0	0.0	0	0.0	0	0.0	0	0.0	1	0.1	0	0.0	0	0.0	>0.99	>0.99
Conjunctival disorder	0	0.0	0	0.0	0	0.0	0	0.0	0	0.0	1	0.1	0	0.0	0	0.0	>0.99	>0.99
Cough	66	6.3	0	0.0	0	0.0	0	0.0	59	5.6	1	0.1	0	0.0	0	0.0	0.58	>0.99
Dehydration	0	0.0	0	0.0	0	0.0	0	0.0	0	0.0	1	0.1	0	0.0	0	0.0	>0.99	>0.99
Dermatitis allergic	0	0.0	0	0.0	0	0.0	0	0.0	0	0.0	1	0.1	0	0.0	0	0.0	>0.99	>0.99
Diabetes mellitus	0	0.0	1	0.1	0	0.0	0	0.0	0	0.0	0	0.0	0	0.0	0	0.0	0.50	0.50
Dizziness	15	1.4	1	0.1	0	0.0	0	0.0	28	2.6	0	0.0	0	0.0	0	0.0	0.093	0.50
Drug hypersensitivity	0	0.0	1	0.1	0	0.0	0	0.0	1	0.1	0	0.0	0	0.0	0	0.0	>0.99	0.50
Dry mouth	51	4.8	0	0.0	0	0.0	0	0.0	51	4.8	1	0.1	0	0.0	0	0.0	>0.99	>0.99
Dysgeusia	146	13.9	1	0.1	0	0.0	0	0.0	166	15.6	1	0.1	0	0.0	0	0.0	0.27	>0.99
Dyspepsia	279	26.5	1	0.1	0	0.0	0	0.0	240	22.6	3	0.3	0	0.0	0	0.0	0.049	0.62
Dysphagia	6	0.6	0	0.0	0	0.0	0	0.0	3	0.3	2	0.2	0	0.0	0	0.0	0.77	0.50
Dyspnoea	34	3.2	1	0.1	2	0.2	0	0.0	30	2.8	2	0.2	0	0.0	0	0.0	0.54	0.69
Gamma-glutamyltransferase	5	0.5	0	0.0	0	0.0	0	0.0	0	0.0	1	0.1	0	0.0	0	0.0	0.12	>0.99
Headache	107	10.2	5	0.5	0	0.0	0	0.0	115	10.8	2	0.2	0	0.0	0	0.0	0.78	0.29

Hot flush	39	3.7	0	0.0	0	0.0	0	0.0	45	4.2	2	0.2	0	0.0	0	0.0	0.44	0.50
Hyperglycaemia	0	0.0	0	0.0	0	0.0	0	0.0	0	0.0	1	0.1	0	0.0	0	0.0	>0.99	>0.99
Hypoxia	0	0.0	0	0.0	1	0.1	0	0.0	0	0.0	0	0.0	0	0.0	0	0.0	0.50	0.50
Insomnia	52	4.9	1	0.1	0	0.0	0	0.0	50	4.7	2	0.2	0	0.0	0	0.0	0.92	>0.99
Intestinal perforation	0	0.0	1	0.1	0	0.0	0	0.0	0	0.0	0	0.0	0	0.0	0	0.0	0.50	0.50
Leukopenia	0	0.0	0	0.0	0	0.0	0	0.0	0	0.0	1	0.1	0	0.0	0	0.0	>0.99	>0.99
Liver function test abnormal	38	3.6	3	0.3	0	0.0	0	0.0	30	2.8	3	0.3	0	0.0	0	0.0	0.35	>0.99
Lymphoedema	13	1.2	2	0.2	0	0.0	0	0.0	21	2.0	0	0.0	0	0.0	0	0.0	0.40	0.25
Malaise	2	0.2	1	0.1	0	0.0	0	0.0	5	0.5	0	0.0	0	0.0	0	0.0	0.73	0.50
Menstruation irregular	2	0.2	0	0.0	0	0.0	0	0.0	2	0.2	1	0.1	0	0.0	0	0.0	>0.99	>0.99
Musculoskeletal pain	4	0.4	1	0.1	0	0.0	0	0.0	3	0.3	2	0.2	1	0.1	0	0.0	>0.99	0.62
Myalgia	32	3.0	1	0.1	0	0.0	0	0.0	47	4.4	2	0.2	0	0.0	0	0.0	0.09	>0.99
Non-cardiac chest pain	4	0.4	1	0.1	0	0.0	0	0.0	2	0.2	0	0.0	1	0.1	0	0.0	0.51	>0.99
Oral candidiasis	11	1.0	1	0.1	0	0.0	0	0.0	5	0.5	0	0.0	0	0.0	0	0.0	0.094	0.50
Oropharyngeal pain	48	4.6	1	0.1	0	0.0	0	0.0	52	4.9	1	0.1	1	0.1	0	0.0	0.69	>0.99
Orthostatic hypotension	0	0.0	0	0.0	0	0.0	0	0.0	1	0.1	1	0.1	0	0.0	0	0.0	0.50	>0.99
Pain	5	0.5	1	0.1	0	0.0	0	0.0	4	0.4	0	0.0	0	0.0	0	0.0	0.55	0.50
Pericardial effusion	0	0.0	0	0.0	0	0.0	0	0.0	0	0.0	1	0.1	0	0.0	0	0.0	>0.99	>0.99
Presyncope	1	0.1	0	0.0	0	0.0	0	0.0	1	0.1	1	0.1	0	0.0	0	0.0	>0.99	>0.99
Pulmonary embolism	0	0.0	0	0.0	0	0.0	0	0.0	0	0.0	0	0.0	1	0.1	0	0.0	>0.99	>0.99
Pyrexia	27	2.6	2	0.2	0	0.0	0	0.0	20	1.9	1	0.1	0	0.0	0	0.0	0.25	0.62
Sepsis	1	0.1	2	0.2	0	0.0	0	0.0	0	0.0	1	0.1	0	0.0	0	0.0	0.37	0.62
Syncope	0	0.0	0	0.0	0	0.0	0	0.0	2	0.2	2	0.2	0	0.0	0	0.0	0.12	0.50
Tooth abscess	2	0.2	1	0.1	0	0.0	0	0.0	2	0.2	0	0.0	0	0.0	0	0.0	0.69	0.50
Toothache	6	0.6	0	0.0	0	0.0	0	0.0	3	0.3	1	0.1	0	0.0	0	0.0	0.55	>0.99
Transaminases increased	6	0.6	2	0.2	0	0.0	0	0.0	5	0.5	0	0.0	0	0.0	0	0.0	0.42	0.25
Tremor	3	0.3	0	0.0	0	0.0	0	0.0	2	0.2	1	0.1	0	0.0	0	0.0	>0.99	>0.99
Vulvovaginal candidiasis	12	1.1	1	0.1	0	0.0	0	0.0	8	0.8	2	0.2	0	0.0	0	0.0	0.54	>0.99

a - Trend tests combine grade 3, 4 and 5 adverse events

b - CTC grades 3 and 4 are not applicable for alopecia or superficial thrombophlebitis

c - Free text other adverse events have been MedDRA coded. Preferred terms are included here

Table A11 - Frequency of adverse events during cycles 5-8 of chemotherapy by CMF/X (QOL substudy patients only)

Adverse events are included if occurring either in >10% of patients at grade ≤2, or at grade ≥3 by any patient during cycles 5-8

	CMF (N = 1030)								X (N = 1044)								Trend test: p-value[a]	Grade ≥3: Fisher's exact p-value
	Grade 1 + 2		Grade 3		Grade 4		Grade 5		Grade 1 + 2		Grade 3		Grade 4		Grade 5			
	n	%	n	%	n	%	n	%	n	%	n	%	n	%	n	%		
**Pre-specified CRF adverse events**																		
Fatigue	825	80.1	107	10.4	2	0.2	0	0.0	773	74.0	52	5.0	0	0.0	0	0.0	<0.0001	<0.0001
Nausea	675	65.5	21	2.0	2	0.2	0	0.0	548	52.5	7	0.7	0	0.0	0	0.0	<0.0001	0.0029
Vomiting	303	29.4	23	2.2	0	0.0	0	0.0	203	19.4	14	1.3	1	0.1	0	0.0	<0.0001	0.19
Alopecia[b]	963	93.5	0	0.0	0	0.0	0	0.0	902	86.4	0	0.0	0	0.0	0	0.0	<0.0001	-
Neuropathy	162	15.7	2	0.2	0	0.0	0	0.0	225	21.6	2	0.2	1	0.1	0	0.0	0.00027	>0.99
Mucositis/Stomatitis	617	59.9	48	4.7	4	0.4	0	0.0	426	40.8	9	0.9	1	0.1	0	0.0	<0.0001	<0.0001
Hand-foot syndrome	118	11.5	3	0.3	0	0.0	0	0.0	649	62.2	129	12.4	0	0.0	0	0.0	<0.0001	<0.0001
Skin	209	20.3	0	0.0	0	0.0	0	0.0	289	27.7	4	0.4	0	0.0	0	0.0	0.89	0.12
Thrombosis/Embolism	16	1.6	14	1.4	7	0.7	0	0.0	14	1.3	5	0.5	2	0.2	0	0.0	0.014	0.0074
Superficial thrombophlebitis[b]	162	15.7	0	0.0	0	0.0	0	0.0	144	13.8	0	0.0	0	0.0	0	0.0	0.21	-
Diarrhoea	434	42.1	44	4.3	2	0.2	0	0.0	490	46.9	63	6.0	4	0.4	0	0.0	0.0069	0.053
Constipation	476	46.2	4	0.4	0	0.0	0	0.0	189	18.1	2	0.2	0	0.0	0	0.0	<0.0001	0.45
Infection	260	25.2	96	9.3	6	0.6	1	0.1	200	19.2	18	1.7	0	0.0	0	0.0	<0.0001	<0.0001
Anaemia	411	39.9	29	2.8	1	0.1	0	0.0	250	23.9	4	0.4	2	0.2	0	0.0	<0.0001	<0.0001
Leucopenia	376	36.5	95	9.2	80	7.8	0	0.0	136	13.0	7	0.7	1	0.1	0	0.0	<0.0001	<0.0001
Neutropenia	263	25.5	155	15.0	166	16.1	0	0.0	165	15.8	17	1.6	6	0.6	0	0.0	<0.0001	<0.0001
Thrombocytopenia	109	10.6	13	1.3	3	0.3	0	0.0	32	3.1	1	0.1	1	0.1	0	0.0	<0.0001	0.0065
Febrile Neutropenia	0	0.0	96	9.3	19	1.8	0	0.0	0	0.0	7	0.7	1	0.1	0	0.0	<0.0001	<0.0001
	CMF (N = 1030)								X (N = 1044)								Any grade: Fisher's exact p-value	Grade ≥3: Fisher's exact p-value
	Grade 1 + 2		Grade 3		Grade 4		Grade 5		Grade 1 + 2		Grade 3		Grade 4		Grade 5			
	n	%	n	%	n	%	n	%	n	%	n	%	n	%	n	%		
**Other MedDRA coded adverse events[c]**																		
Abdominal discomfort	5	0.5	0	0.0	0	0.0	0	0.0	7	0.7	1	0.1	0	0.0	0	0.0	0.58	>0.99
Abdominal distension	12	1.2	1	0.1	0	0.0	0	0.0	4	0.4	0	0.0	0	0.0	0	0.0	0.029	0.50
Abdominal pain	24	2.3	0	0.0	0	0.0	0	0.0	33	3.2	3	0.3	0	0.0	0	0.0	0.15	0.25
Abnormal weight gain	8	0.8	1	0.1	0	0.0	0	0.0	4	0.4	0	0.0	0	0.0	0	0.0	0.18	0.50
Acute myocardial infarction	0	0.0	0	0.0	0	0.0	0	0.0	0	0.0	0	0.0	1	0.1	0	0.0	>0.99	>0.99
Acute respiratory distress syndrome	0	0.0	0	0.0	0	0.0	0	0.0	0	0.0	0	0.0	0	0.0	1	0.1	>0.99	>0.99
Alanine aminotransferase increased	54	5.2	2	0.2	1	0.1	0	0.0	33	3.2	3	0.3	0	0.0	0	0.0	0.026	>0.99
Amenorrhoea	0	0.0	2	0.2	1	0.1	0	0.0	0	0.0	0	0.0	0	0.0	0	0.0	0.12	0.12
Anorexia	21	2.0	3	0.3	0	0.0	0	0.0	26	2.5	0	0.0	0	0.0	0	0.0	0.89	0.12
Anxiety	17	1.7	1	0.1	0	0.0	0	0.0	18	1.7	1	0.1	0	0.0	0	0.0	>0.99	>0.99
Asthenia	2	0.2	2	0.2	0	0.0	0	0.0	0	0.0	0	0.0	0	0.0	0	0.0	0.061	0.25
Back pain	26	2.5	1	0.1	0	0.0	0	0.0	22	2.1	0	0.0	0	0.0	0	0.0	0.47	0.50
Blood bilirubin increased	5	0.5	0	0.0	0	0.0	0	0.0	13	1.2	1	0.1	0	0.0	0	0.0	0.062	>0.99
Blood glucose increased	1	0.1	1	0.1	0	0.0	0	0.0	1	0.1	0	0.0	0	0.0	0	0.0	0.62	0.50
C-reactive protein abnormal	0	0.0	1	0.1	0	0.0	0	0.0	0	0.0	0	0.0	0	0.0	0	0.0	0.50	0.50
Cardiotoxicity	0	0.0	0	0.0	0	0.0	0	0.0	2	0.2	1	0.1	0	0.0	0	0.0	0.25	>0.99
Catheter related infection	1	0.1	1	0.1	0	0.0	0	0.0	0	0.0	0	0.0	0	0.0	0	0.0	0.25	0.50
Cellulitis	2	0.2	1	0.1	0	0.0	0	0.0	1	0.1	0	0.0	0	0.0	0	0.0	0.37	0.50
Cerebrovascular accident	0	0.0	0	0.0	0	0.0	0	0.0	0	0.0	0	0.0	0	0.0	1	0.1	>0.99	>0.99
Chest pain	5	0.5	0	0.0	0	0.0	0	0.0	15	1.4	3	0.3	0	0.0	0	0.0	0.010	0.25
Cholelithiasis	0	0.0	1	0.1	0	0.0	0	0.0	0	0.0	0	0.0	0	0.0	0	0.0	0.50	0.50
Confusional state	0	0.0	1	0.1	0	0.0	0	0.0	0	0.0	0	0.0	0	0.0	0	0.0	0.50	0.50
Deep vein thrombosis	2	0.2	1	0.1	0	0.0	0	0.0	0	0.0	0	0.0	0	0.0	0	0.0	0.12	0.50

Dehydration	0	0.0	0	0.0	0	0.0	0	0.0	0	0.0	0	0.0	1	0.1	0	0.0	>0.99	>0.99
Depressed mood	28	2.7	1	0.1	0	0.0	0	0.0	33	3.2	1	0.1	0	0.0	0	0.0	0.61	>0.99
Depression	14	1.4	0	0.0	0	0.0	0	0.0	16	1.5	2	0.2	0	0.0	0	0.0	0.59	0.50
Dihydropyrimidine dehydrogenase deficiency	0	0.0	0	0.0	0	0.0	0	0.0	0	0.0	0	0.0	1	0.1	0	0.0	>0.99	>0.99
Dizziness	34	3.3	1	0.1	0	0.0	0	0.0	25	2.4	2	0.2	0	0.0	0	0.0	0.30	>0.99
Dry mouth	60	5.8	1	0.1	0	0.0	0	0.0	80	7.7	1	0.1	0	0.0	0	0.0	0.10	>0.99
Dysgeusia	112	10.9	2	0.2	0	0.0	0	0.0	116	11.1	0	0.0	0	0.0	0	0.0	>0.99	0.25
Dyspnoea	66	6.4	7	0.7	2	0.2	0	0.0	31	3.0	1	0.1	2	0.2	0	0.0	<0.0001	0.089
Gastroenteritis	2	0.2	1	0.1	0	0.0	0	0.0	1	0.1	0	0.0	0	0.0	0	0.0	0.37	0.50
Guillain-Barre syndrome	0	0.0	0	0.0	1	0.1	0	0.0	0	0.0	0	0.0	0	0.0	0	0.0	0.50	0.50
Headache	44	4.3	1	0.1	0	0.0	0	0.0	44	4.2	1	0.1	0	0.0	0	0.0	>0.99	>0.99
Hot flush	94	9.1	1	0.1	1	0.1	0	0.0	55	5.3	0	0.0	0	0.0	0	0.0	0.00038	0.25
Hyperglycaemia	0	0.0	4	0.4	0	0.0	0	0.0	0	0.0	0	0.0	0	0.0	0	0.0	0.061	0.061
Hypokalaemia	4	0.4	0	0.0	1	0.1	0	0.0	1	0.1	2	0.2	0	0.0	0	0.0	0.50	>0.99
Hypomagnesaemia	0	0.0	0	0.0	1	0.1	0	0.0	1	0.1	0	0.0	0	0.0	0	0.0	>0.99	0.50
Hyponatraemia	0	0.0	1	0.1	0	0.0	0	0.0	0	0.0	0	0.0	1	0.1	0	0.0	>0.99	>0.99
Hypophosphataemia	0	0.0	1	0.1	0	0.0	0	0.0	0	0.0	0	0.0	0	0.0	0	0.0	0.50	0.50
Hypotension	2	0.2	0	0.0	1	0.1	0	0.0	0	0.0	0	0.0	0	0.0	0	0.0	0.12	0.50
Hypoxia	0	0.0	1	0.1	0	0.0	0	0.0	0	0.0	0	0.0	1	0.1	0	0.0	>0.99	>0.99
Insomnia	42	4.1	0	0.0	0	0.0	0	0.0	24	2.3	2	0.2	0	0.0	0	0.0	0.048	0.50
International normalised ratio increased	0	0.0	0	0.0	0	0.0	0	0.0	0	0.0	1	0.1	0	0.0	0	0.0	>0.99	>0.99
Intestinal ischaemia	0	0.0	0	0.0	1	0.1	0	0.0	0	0.0	0	0.0	1	0.1	0	0.0	>0.99	>0.99
Ischaemic stroke	0	0.0	0	0.0	0	0.0	0	0.0	0	0.0	0	0.0	0	0.0	1	0.1	>0.99	>0.99
Labyrinthitis	0	0.0	1	0.1	0	0.0	0	0.0	0	0.0	0	0.0	0	0.0	0	0.0	0.50	0.50
Lacrimation increased	81	7.9	0	0.0	0	0.0	0	0.0	52	5.0	1	0.1	0	0.0	0	0.0	0.012	>0.99
Lethargy	0	0.0	1	0.1	0	0.0	0	0.0	0	0.0	0	0.0	0	0.0	0	0.0	0.50	0.50
Liver function test abnormal	36	3.5	2	0.2	0	0.0	0	0.0	29	2.8	1	0.1	0	0.0	0	0.0	0.32	0.62
Menstrual disorder	2	0.2	1	0.1	0	0.0	0	0.0	4	0.4	0	0.0	0	0.0	0	0.0	>0.99	0.50
Menstruation irregular	3	0.3	3	0.3	0	0.0	0	0.0	2	0.2	0	0.0	0	0.0	0	0.0	0.18	0.12
Migraine	2	0.2	1	0.1	0	0.0	0	0.0	6	0.6	2	0.2	0	0.0	0	0.0	0.23	>0.99
Mouth ulceration	4	0.4	1	0.1	0	0.0	0	0.0	4	0.4	0	0.0	0	0.0	0	0.0	0.75	0.50
Multi-organ failure	0	0.0	0	0.0	1	0.1	0	0.0	0	0.0	0	0.0	0	0.0	0	0.0	0.50	0.50
Musculoskeletal pain	6	0.6	1	0.1	0	0.0	0	0.0	6	0.6	0	0.0	0	0.0	0	0.0	0.79	0.50
Non-cardiac chest pain	6	0.6	2	0.2	0	0.0	0	0.0	11	1.1	7	0.7	1	0.1	0	0.0	0.051	0.11
Oral candidiasis	13	1.3	1	0.1	0	0.0	0	0.0	1	0.1	0	0.0	0	0.0	0	0.0	0.00046	0.50
Oral pain	3	0.3	0	0.0	1	0.1	0	0.0	1	0.1	0	0.0	0	0.0	0	0.0	0.22	0.50
Pain in extremity	28	2.7	1	0.1	0	0.0	0	0.0	35	3.4	0	0.0	0	0.0	0	0.0	0.53	0.50
Pancreatitis acute	0	0.0	1	0.1	0	0.0	0	0.0	0	0.0	0	0.0	0	0.0	0	0.0	0.50	0.50
Peripheral ischaemia	0	0.0	0	0.0	1	0.1	0	0.0	0	0.0	0	0.0	0	0.0	0	0.0	0.50	0.50
Photophobia	1	0.1	1	0.1	0	0.0	0	0.0	0	0.0	0	0.0	0	0.0	0	0.0	0.25	0.50
Pulmonary embolism	0	0.0	0	0.0	0	0.0	0	0.0	0	0.0	0	0.0	1	0.1	0	0.0	>0.99	>0.99
Quality of life decreased	0	0.0	1	0.1	0	0.0	0	0.0	0	0.0	0	0.0	0	0.0	0	0.0	0.50	0.50
Renal failure	0	0.0	0	0.0	0	0.0	0	0.0	0	0.0	1	0.1	0	0.0	0	0.0	>0.99	>0.99
Sinus tachycardia	1	0.1	0	0.0	0	0.0	0	0.0	0	0.0	0	0.0	1	0.1	0	0.0	>0.99	>0.99
Skin ulcer	2	0.2	0	0.0	0	0.0	0	0.0	0	0.0	1	0.1	0	0.0	0	0.0	0.62	>0.99
Stomatitis	7	0.7	1	0.1	0	0.0	0	0.0	3	0.3	0	0.0	0	0.0	0	0.0	0.14	0.50
Syncope	0	0.0	1	0.1	0	0.0	0	0.0	1	0.1	1	0.1	0	0.0	0	0.0	>0.99	>0.99
Tachycardia	3	0.3	1	0.1	0	0.0	0	0.0	0	0.0	0	0.0	0	0.0	0	0.0	0.061	0.50
Vein pain	5	0.5	1	0.1	0	0.0	0	0.0	3	0.3	0	0.0	0	0.0	0	0.0	0.34	0.50
Vertigo	1	0.1	0	0.0	0	0.0	0	0.0	0	0.0	1	0.1	0	0.0	0	0.0	>0.99	>0.99
Vulvovaginal candidiasis	5	0.5	1	0.1	0	0.0	0	0.0	10	1.0	0	0.0	0	0.0	0	0.0	0.45	0.50

a - Trend tests combine grade 3, 4 and 5 adverse events
b - CTC grades 3 and 4 are not applicable for alopecia or superficial thrombophlebitis
c - Free text other adverse events have been MedDRA coded. Preferred terms are included here

Table A12 - Frequency of adverse events during cycles 1-8 of chemotherapy by CMF/X (QOL substudy patients only)

Adverse events are included if occurring either in >10% of patients at grade ≤2, or at grade ≥3 by any patient during cycles 1-8

	CMF (N = 1053)								X (N = 1062)								Trend test: p-value[a]	Grade ≥3: Fisher's exact p-value
	Grade 1 + 2		Grade 3		Grade 4		Grade 5		Grade 1 + 2		Grade 3		Grade 4		Grade 5			
	n	%	n	%	n	%	n	%	n	%	n	%	n	%	n	%		
**Pre-specified CRF adverse events**																		
Fatigue	870	82.6	137	13.0	4	0.4	0	0.0	917	86.3	90	8.5	3	0.3	0	0.0	<0.0001	0.00085
Nausea	869	82.5	41	3.9	2	0.2	0	0.0	873	82.2	25	2.4	1	0.1	0	0.0	0.0036	0.037
Vomiting	484	46.0	42	4.0	3	0.3	0	0.0	437	41.1	35	3.3	1	0.1	0	0.0	0.0024	0.31
Alopecia[b]	1029	97.7	0	0.0	0	0.0	0	0.0	1022	96.2	0	0.0	0	0.0	0	0.0	0.0072	-
Neuropathy	237	22.5	4	0.4	0	0.0	0	0.0	277	26.1	3	0.3	1	0.1	0	0.0	0.044	>0.99
Mucositis/Stomatitis	813	77.2	59	5.6	4	0.4	0	0.0	817	76.9	17	1.6	1	0.1	0	0.0	<0.0001	<0.0001
Hand-foot syndrome	157	14.9	7	0.7	0	0.0	0	0.0	651	61.3	132	12.4	0	0.0	0	0.0	<0.0001	<0.0001
Skin	450	42.7	3	0.3	0	0.0	0	0.0	451	42.5	8	0.8	0	0.0	0	0.0	0.82	0.23
Thrombosis/Embolism	22	2.1	16	1.5	7	0.7	0	0.0	16	1.5	9	0.8	4	0.4	0	0.0	0.046	0.095
Superficial thrombophlebitis[b]	280	26.6	0	0.0	0	0.0	0	0.0	286	26.9	0	0.0	0	0.0	0	0.0	0.86	-
Diarrhoea	542	51.5	52	4.9	2	0.2	0	0.0	596	56.1	78	7.3	4	0.4	0	0.0	0.0011	0.017
Constipation	771	73.2	8	0.8	0	0.0	0	0.0	715	67.3	9	0.8	0	0.0	0	0.0	0.0099	>0.99
Infection	416	39.5	128	12.2	10	0.9	1	0.1	408	38.4	51	4.8	1	0.1	0	0.0	<0.0001	<0.0001
Anaemia	512	48.6	30	2.8	1	0.1	0	0.0	389	36.6	4	0.4	2	0.2	0	0.0	<0.0001	<0.0001
Leucopenia	397	37.7	115	10.9	84	8.0	0	0.0	230	21.7	23	2.2	3	0.3	0	0.0	<0.0001	<0.0001
Neutropenia	300	28.5	170	16.1	194	18.4	0	0.0	259	24.4	83	7.8	30	2.8	0	0.0	<0.0001	<0.0001
Thrombocytopenia	121	11.5	14	1.3	4	0.4	0	0.0	51	4.8	1	0.1	1	0.1	0	0.0	<0.0001	0.0002
Febrile Neutropenia	0	0.0	116	11.0	21	2.0	0	0.0	0	0.0	32	3.0	4	0.4	0	0.0	<0.0001	<0.0001

	CMF (N = 1053)								X (N = 1062)								Any grade: Fisher's exact p-value	Grade ≥3: Fisher's exact p-value
	Grade 1 + 2		Grade 3		Grade 4		Grade 5		Grade 1 + 2		Grade 3		Grade 4		Grade 5			
	n	%	n	%	n	%	n	%	n	%	n	%	n	%	n	%		
**Other MedDRA coded adverse events[c]**																		
Abdominal discomfort	7	0.7	0	0.0	0	0.0	0	0.0	9	0.9	1	0.1	0	0.0	0	0.0	0.63	>0.99
Abdominal distension	23	2.1	1	0.1	0	0.0	0	0.0	12	1.1	0	0.0	0	0.0	0	0.0	0.044	0.50
Abdominal pain	39	3.6	1	0.1	0	0.0	0	0.0	44	4.2	5	0.5	0	0.0	0	0.0	0.39	0.22
Abnormal weight gain	12	1.1	1	0.1	0	0.0	0	0.0	5	0.5	0	0.0	0	0.0	0	0.0	0.061	0.50
Acute myocardial infarction	0	0.0	0	0.0	1	0.1	0	0.0	0	0.0	0	0.0	1	0.1	0	0.0	>0.99	>0.99
Acute respiratory distress syndrome	0	0.0	0	0.0	0	0.0	0	0.0	0	0.0	0	0.0	0	0.0	1	0.1	>0.99	>0.99
Alanine aminotransferase	13	1.2	0	0.0	0	0.0	0	0.0	8	0.8	1	0.1	0	0.0	0	0.0	0.40	>0.99
Alanine aminotransferase increased	65	6.1	2	0.2	1	0.1	0	0.0	53	5.1	6	0.6	0	0.0	0	0.0	0.41	0.51
Amenorrhoea	0	0.0	2	0.2	1	0.1	0	0.0	0	0.0	0	0.0	0	0.0	0	0.0	0.12	0.12
Angina pectoris	0	0.0	0	0.0	0	0.0	0	0.0	2	0.2	1	0.1	0	0.0	0	0.0	0.25	>0.99
Anorexia	35	3.3	3	0.3	0	0.0	0	0.0	39	3.7	0	0.0	0	0.0	0	0.0	>0.99	0.12
Anxiety	29	2.7	1	0.1	0	0.0	0	0.0	22	2.1	1	0.1	0	0.0	0	0.0	0.33	>0.99
Arthralgia	86	8.0	1	0.1	0	0.0	0	0.0	77	7.4	2	0.2	0	0.0	0	0.0	0.52	>0.99
Asthenia	4	0.4	2	0.2	0	0.0	0	0.0	1	0.1	0	0.0	0	0.0	0	0.0	0.069	0.25
Back pain	53	5.0	1	0.1	0	0.0	0	0.0	49	4.7	1	0.1	0	0.0	0	0.0	0.69	>0.99
Blood bilirubin increased	5	0.5	0	0.0	0	0.0	0	0.0	14	1.3	2	0.2	0	0.0	0	0.0	0.026	0.50
Blood glucose increased	1	0.1	1	0.1	0	0.0	0	0.0	1	0.1	0	0.0	0	0.0	0	0.0	0.62	0.50
Blood test	0	0.0	1	0.1	0	0.0	0	0.0	0	0.0	0	0.0	0	0.0	0	0.0	0.50	0.50
C-reactive protein abnormal	0	0.0	1	0.1	0	0.0	0	0.0	1	0.1	0	0.0	0	0.0	0	0.0	>0.99	0.50
C-reactive protein increased	0	0.0	1	0.1	0	0.0	0	0.0	1	0.1	0	0.0	0	0.0	0	0.0	>0.99	0.50
Cardiotoxicity	0	0.0	0	0.0	0	0.0	0	0.0	2	0.2	1	0.1	0	0.0	0	0.0	0.25	>0.99

Catheter related infection	1	0.1	1	0.1	0	0.0	0	0.0	0	0.0	0	0.0	0	0.0	0	0.0	0.25	0.50
Cellulitis	4	0.4	1	0.1	0	0.0	0	0.0	2	0.2	0	0.0	0	0.0	0	0.0	0.29	0.50
Cerebrovascular accident	0	0.0	0	0.0	0	0.0	0	0.0	0	0.0	0	0.0	0	0.0	1	0.1	>0.99	>0.99
Chest pain	7	0.7	0	0.0	0	0.0	0	0.0	17	1.6	3	0.3	0	0.0	0	0.0	0.018	0.25
Cholelithiasis	0	0.0	1	0.1	0	0.0	0	0.0	0	0.0	0	0.0	0	0.0	0	0.0	0.50	0.50
Colitis	2	0.2	0	0.0	0	0.0	0	0.0	0	0.0	1	0.1	0	0.0	0	0.0	0.62	>0.99
Confusional state	2	0.2	1	0.1	0	0.0	0	0.0	0	0.0	0	0.0	0	0.0	0	0.0	0.12	0.50
Conjunctival disorder	0	0.0	0	0.0	0	0.0	0	0.0	0	0.0	1	0.1	0	0.0	0	0.0	>0.99	>0.99
Cough	101	9.4	0	0.0	0	0.0	0	0.0	83	7.9	1	0.1	0	0.0	0	0.0	0.19	>0.99
Deep vein thrombosis	2	0.2	1	0.1	0	0.0	0	0.0	0	0.0	0	0.0	0	0.0	0	0.0	0.12	0.50
Dehydration	0	0.0	0	0.0	0	0.0	0	0.0	0	0.0	1	0.1	1	0.1	0	0.0	0.50	0.50
Depressed mood	52	4.9	1	0.1	0	0.0	0	0.0	61	5.8	1	0.1	0	0.0	0	0.0	0.44	>0.99
Depression	25	2.3	0	0.0	0	0.0	0	0.0	26	2.5	2	0.2	0	0.0	0	0.0	0.78	0.50
Dermatitis allergic	0	0.0	0	0.0	0	0.0	0	0.0	1	0.1	1	0.1	0	0.0	0	0.0	0.50	>0.99
Diabetes mellitus	1	0.1	1	0.1	0	0.0	0	0.0	0	0.0	0	0.0	0	0.0	0	0.0	0.25	0.50
Dihydropyrimidine dehydrogenase deficiency	0	0.0	0	0.0	0	0.0	0	0.0	0	0.0	0	0.0	1	0.1	0	0.0	>0.99	>0.99
Dizziness	44	4.1	2	0.2	0	0.0	0	0.0	47	4.5	2	0.2	0	0.0	0	0.0	0.83	1.00
Drug hypersensitivity	0	0.0	1	0.1	0	0.0	0	0.0	1	0.1	0	0.0	0	0.0	0	0.0	>0.99	0.50
Dry mouth	93	8.7	1	0.1	0	0.0	0	0.0	106	10.1	2	0.2	0	0.0	0	0.0	0.34	>0.99
Dysgeusia	199	18.6	3	0.3	0	0.0	0	0.0	212	20.3	1	0.1	0	0.0	0	0.0	0.62	0.37
Dyspepsia	328	30.7	1	0.1	0	0.0	0	0.0	266	25.5	3	0.3	0	0.0	0	0.0	0.0027	0.62
Dysphagia	8	0.7	0	0.0	0	0.0	0	0.0	3	0.3	2	0.2	0	0.0	0	0.0	0.42	0.50
Dyspnoea	83	7.8	7	0.7	4	0.4	0	0.0	54	5.2	3	0.3	2	0.2	0	0.0	0.0032	0.14
Gamma-glutamyltransferase	6	0.6	0	0.0	0	0.0	0	0.0	0	0.0	1	0.1	0	0.0	0	0.0	0.069	>0.99
Gastroenteritis	3	0.3	1	0.1	0	0.0	0	0.0	1	0.1	0	0.0	0	0.0	0	0.0	0.22	0.50
Guillain-Barre syndrome	0	0.0	0	0.0	1	0.1	0	0.0	0	0.0	0	0.0	0	0.0	0	0.0	0.50	0.50
Headache	134	12.5	6	0.6	0	0.0	0	0.0	148	14.2	3	0.3	0	0.0	0	0.0	0.57	0.34
Hot flush	113	10.6	1	0.1	1	0.1	0	0.0	87	8.3	2	0.2	0	0.0	0	0.0	0.055	>0.99
Hyperglycaemia	0	0.0	4	0.4	0	0.0	0	0.0	0	0.0	1	0.1	0	0.0	0	0.0	0.22	0.22
Hypokalaemia	4	0.4	0	0.0	1	0.1	0	0.0	2	0.2	2	0.2	0	0.0	0	0.0	0.75	>0.99
Hypomagnesaemia	0	0.0	0	0.0	1	0.1	0	0.0	1	0.1	0	0.0	0	0.0	0	0.0	>0.99	0.50
Hyponatraemia	0	0.0	1	0.1	0	0.0	0	0.0	0	0.0	0	0.0	1	0.1	0	0.0	>0.99	>0.99
Hypophosphataemia	0	0.0	1	0.1	0	0.0	0	0.0	0	0.0	0	0.0	0	0.0	0	0.0	0.50	0.50
Hypotension	3	0.3	0	0.0	1	0.1	0	0.0	2	0.2	0	0.0	0	0.0	0	0.0	0.45	0.50
Hypoxia	0	0.0	1	0.1	1	0.1	0	0.0	0	0.0	0	0.0	1	0.1	0	0.0	0.62	0.62
Insomnia	80	7.5	1	0.1	0	0.0	0	0.0	66	6.3	4	0.4	0	0.0	0	0.0	0.35	0.37
International normalised ratio increased	0	0.0	0	0.0	0	0.0	0	0.0	0	0.0	1	0.1	0	0.0	0	0.0	>0.99	>0.99
Intestinal ischaemia	0	0.0	0	0.0	1	0.1	0	0.0	0	0.0	0	0.0	1	0.1	0	0.0	>0.99	>0.99
Intestinal perforation	0	0.0	1	0.1	0	0.0	0	0.0	0	0.0	0	0.0	0	0.0	0	0.0	0.50	0.50
Ischaemic stroke	0	0.0	0	0.0	0	0.0	0	0.0	0	0.0	0	0.0	0	0.0	1	0.1	>0.99	>0.99
Labyrinthitis	0	0.0	1	0.1	0	0.0	0	0.0	0	0.0	0	0.0	0	0.0	0	0.0	0.50	0.50
Lacrimation increased	125	11.7	0	0.0	0	0.0	0	0.0	117	11.2	1	0.1	0	0.0	0	0.0	0.59	>0.99
Lethargy	0	0.0	1	0.1	0	0.0	0	0.0	0	0.0	0	0.0	0	0.0	0	0.0	0.50	0.50
Leukopenia	1	0.1	0	0.0	0	0.0	0	0.0	0	0.0	1	0.1	0	0.0	0	0.0	>0.99	>0.99
Liver function test abnormal	60	5.6	4	0.4	0	0.0	0	0.0	47	4.5	4	0.4	0	0.0	0	0.0	0.21	>0.99
Lymphoedema	39	3.6	2	0.2	0	0.0	0	0.0	34	3.3	0	0.0	0	0.0	0	0.0	0.41	0.25
Malaise	4	0.4	1	0.1	0	0.0	0	0.0	5	0.5	0	0.0	0	0.0	0	0.0	>0.99	0.50
Menstrual disorder	4	0.4	1	0.1	0	0.0	0	0.0	4	0.4	0	0.0	0	0.0	0	0.0	0.75	0.50
Menstruation irregular	4	0.4	3	0.3	0	0.0	0	0.0	4	0.4	1	0.1	0	0.0	0	0.0	0.58	0.37
Migraine	5	0.5	1	0.1	0	0.0	0	0.0	17	1.6	2	0.2	0	0.0	0	0.0	0.014	>0.99
Mouth ulceration	8	0.7	1	0.1	0	0.0	0	0.0	6	0.6	0	0.0	0	0.0	0	0.0	0.45	0.50
Multi-organ failure	0	0.0	0	0.0	1	0.1	0	0.0	0	0.0	0	0.0	0	0.0	0	0.0	0.50	0.50
Musculoskeletal pain	10	0.9	2	0.2	0	0.0	0	0.0	9	0.9	2	0.2	1	0.1	0	0.0	>0.99	>0.99
Myalgia	60	5.6	1	0.1	0	0.0	0	0.0	63	6.0	2	0.2	0	0.0	0	0.0	0.78	>0.99
Non-cardiac chest pain	9	0.8	3	0.3	0	0.0	0	0.0	13	1.2	7	0.7	2	0.2	0	0.0	0.12	0.14
Oral candidiasis	21	2.0	2	0.2	0	0.0	0	0.0	6	0.6	0	0.0	0	0.0	0	0.0	0.0013	0.25
Oral pain	6	0.6	0	0.0	1	0.1	0	0.0	4	0.4	0	0.0	0	0.0	0	0.0	0.38	0.50
Oropharyngeal pain	66	6.2	1	0.1	0	0.0	0	0.0	67	6.4	1	0.1	1	0.1	0	0.0	0.93	>0.99
Orthostatic hypotension	2	0.2	0	0.0	0	0.0	0	0.0	1	0.1	1	0.1	0	0.0	0	0.0	>0.99	>0.99

Pain	9	0.8	1	0.1	0	0.0	0	0.0	7	0.7	0	0.0	0	0.0	0	0.0	0.48	0.50
Pain in extremity	60	5.6	1	0.1	0	0.0	0	0.0	74	7.1	0	0.0	0	0.0	0	0.0	0.29	0.50
Pancreatitis acute	0	0.0	1	0.1	0	0.0	0	0.0	0	0.0	0	0.0	0	0.0	0	0.0	0.50	0.50
Pericardial effusion	0	0.0	0	0.0	0	0.0	0	0.0	0	0.0	1	0.1	0	0.0	0	0.0	>0.99	>0.99
Peripheral ischaemia	0	0.0	0	0.0	1	0.1	0	0.0	0	0.0	0	0.0	0	0.0	0	0.0	0.50	0.50
Photophobia	1	0.1	1	0.1	0	0.0	0	0.0	0	0.0	0	0.0	0	0.0	0	0.0	0.25	0.50
Presyncope	1	0.1	0	0.0	0	0.0	0	0.0	1	0.1	1	0.1	0	0.0	0	0.0	>0.99	>0.99
Pulmonary embolism	0	0.0	0	0.0	0	0.0	0	0.0	0	0.0	0	0.0	2	0.2	0	0.0	0.50	0.50
Pyrexia	59	5.5	2	0.2	0	0.0	0	0.0	33	3.2	1	0.1	0	0.0	0	0.0	0.0044	0.62
Quality of life decreased	0	0.0	1	0.1	0	0.0	0	0.0	0	0.0	0	0.0	0	0.0	0	0.0	0.50	0.50
Renal failure	0	0.0	0	0.0	0	0.0	0	0.0	0	0.0	1	0.1	0	0.0	0	0.0	>0.99	>0.99
Sepsis	1	0.1	2	0.2	0	0.0	0	0.0	0	0.0	1	0.1	0	0.0	0	0.0	0.37	0.62
Sinus tachycardia	1	0.1	0	0.0	0	0.0	0	0.0	0	0.0	0	0.0	1	0.1	0	0.0	>0.99	>0.99
Skin ulcer	3	0.3	0	0.0	0	0.0	0	0.0	1	0.1	1	0.1	0	0.0	0	0.0	0.69	>0.99
Stomatitis	14	1.3	1	0.1	0	0.0	0	0.0	11	1.1	0	0.0	0	0.0	0	0.0	0.44	0.50
Syncope	0	0.0	1	0.1	0	0.0	0	0.0	3	0.3	3	0.3	0	0.0	0	0.0	0.12	0.62
Tachycardia	3	0.3	1	0.1	0	0.0	0	0.0	1	0.1	0	0.0	0	0.0	0	0.0	0.22	0.50
Tooth abscess	2	0.2	1	0.1	0	0.0	0	0.0	3	0.3	0	0.0	0	0.0	0	0.0	>0.99	0.50
Toothache	13	1.2	0	0.0	0	0.0	0	0.0	8	0.8	1	0.1	0	0.0	0	0.0	0.40	>0.99
Transaminases increased	11	1.0	2	0.2	0	0.0	0	0.0	6	0.6	0	0.0	0	0.0	0	0.0	0.11	0.25
Tremor	4	0.4	0	0.0	0	0.0	0	0.0	3	0.3	1	0.1	0	0.0	0	0.0	>0.99	>0.99
Vein pain	16	1.5	1	0.1	0	0.0	0	0.0	17	1.6	0	0.0	0	0.0	0	0.0	>0.99	0.50
Vertigo	4	0.4	0	0.0	0	0.0	0	0.0	0	0.0	1	0.1	0	0.0	0	0.0	0.22	>0.99
Vulvovaginal candidiasis	15	1.4	2	0.2	0	0.0	0	0.0	17	1.6	2	0.2	0	0.0	0	0.0	0.87	>0.99

a - Trend tests combine grade 3, 4 and 5 adverse events

b - CTC grades 3 and 4 are not applicable for alopecia or superficial thrombophlebitis

c - Free text other adverse events have been MedDRA coded. Preferred terms are included here

Table A13 - Frequency of late adverse events by E/aE

Analysis of late safety compared all signs and symptoms reported at or after 12 months from randomisation and include all patients randomised who were followed-up for at least 9 months post-randomisation and who received at least one cycle of allocated chemotherapy. Late safety analysis was censored at the point of disease recurrence or second primary cancer.
Adverse events are included if occurring either in >10% of patients at grade ≤2, or at grade ≥3 by any patient

	E (N = 2193)								aE (N = 2155)								Trend test: p-value[a]	Grade ≥3: Fisher's exact p-value
	Grade 1 + 2		Grade 3		Grade 4		Grade 5		Grade 1 + 2		Grade 3		Grade 4		Grade 5			
	n	%	n	%	n	%	n	%	n	%	n	%	n	%	n	%		
**Pre-specified CRF adverse events**																		
Fatigue	773	35.2	26	1.2	3	0.1	0	0.0	764	35.5	15	0.7	2	0.1	0	0.0	0.63	0.10
Hand-foot syndrome	149	6.8	2	0.1	0	0.0	0	0.0	148	6.9	2	0.1	0	0.0	0	0.0	0.62	>0.99

	E (N = 2193)								aE (N = 2155)								Any grade: Fisher's exact p-value	Grade ≥3: Fisher's exact p-value
	Grade 1 + 2		Grade 3		Grade 4		Grade 5		Grade 1 + 2		Grade 3		Grade 4		Grade 5			
	n	%	n	%	n	%	n	%	n	%	n	%	n	%	n	%		
**Other MedDRA coded adverse events[b]**																		
Abnormal weight gain	19	0.9	1	0.0	0	0.0	0	0.0	24	1.1	2	0.1	0	0.0	0	0.0	0.38	0.62
Ageusia	2	0.1	1	0.0	0	0.0	0	0.0	1	0.0	0	0.0	0	0.0	0	0.0	0.62	>0.99
Anxiety	25	1.1	1	0.0	0	0.0	0	0.0	7	0.3	1	0.0	0	0.0	0	0.0	0.0029	>0.99
Arthralgia	229	10.4	11	0.5	1	0.0	0	0.0	234	10.9	8	0.4	0	0.0	0	0.0	0.81	0.50
Arthritis	11	0.5	1	0.0	0	0.0	0	0.0	5	0.2	0	0.0	0	0.0	0	0.0	0.14	1.00
Arthropathy	2	0.1	0	0.0	0	0.0	0	0.0	0	0.0	1	0.0	0	0.0	0	0.0	>0.99	0.50
Asthenia	3	0.1	1	0.0	0	0.0	0	0.0	4	0.2	0	0.0	0	0.0	0	0.0	>0.99	>0.99
Atrial fibrillation	1	0.0	0	0.0	0	0.0	0	0.0	0	0.0	1	0.0	0	0.0	0	0.0	>0.99	0.50
Atrophic vulvovaginitis	1	0.0	0	0.0	0	0.0	0	0.0	0	0.0	1	0.0	0	0.0	0	0.0	>0.99	0.50
Back pain	40	1.8	1	0.0	0	0.0	0	0.0	33	1.5	1	0.0	0	0.0	0	0.0	0.49	>0.99
Balance disorder	1	0.0	1	0.0	0	0.0	0	0.0	0	0.0	0	0.0	0	0.0	0	0.0	0.50	>0.99
Bone pain	9	0.4	1	0.0	0	0.0	0	0.0	10	0.5	1	0.0	0	0.0	0	0.0	0.83	>0.99
Cardiac disorder	1	0.0	0	0.0	0	0.0	0	0.0	0	0.0	1	0.0	0	0.0	0	0.0	>0.99	0.50
Cardiac failure	0	0.0	0	0.0	0	0.0	0	0.0	1	0.0	2	0.1	0	0.0	0	0.0	0.12	0.25
Cardio-respiratory arrest	0	0.0	0	0.0	0	0.0	0	0.0	0	0.0	0	0.0	1	0.0	0	0.0	0.50	0.50
Cardiomyopathy	1	0.0	0	0.0	1	0.0	0	0.0	1	0.0	1	0.0	0	0.0	0	0.0	>0.99	>0.99
Carpal tunnel syndrome	7	0.3	1	0.0	0	0.0	0	0.0	7	0.3	0	0.0	0	0.0	0	0.0	>0.99	>0.99
Cataract	2	0.1	2	0.1	0	0.0	0	0.0	0	0.0	1	0.0	0	0.0	0	0.0	0.37	>0.99
Catheter related infection	0	0.0	1	0.0	0	0.0	0	0.0	0	0.0	0	0.0	0	0.0	0	0.0	>0.99	>0.99
Cerebrovascular accident	0	0.0	0	0.0	0	0.0	0	0.0	1	0.0	0	0.0	1	0.0	0	0.0	0.25	0.50
Deep vein thrombosis	1	0.0	0	0.0	0	0.0	0	0.0	1	0.0	1	0.0	0	0.0	0	0.0	0.62	0.50
Dementia	0	0.0	1	0.0	0	0.0	0	0.0	1	0.0	0	0.0	0	0.0	0	0.0	>0.99	>0.99
Depression	23	1.0	2	0.1	1	0.0	0	0.0	23	1.1	3	0.1	1	0.0	0	0.0	0.89	0.72
Disturbance in attention	7	0.3	0	0.0	0	0.0	0	0.0	5	0.2	1	0.0	0	0.0	0	0.0	>0.99	0.50
Dry mouth	22	1.0	2	0.1	0	0.0	0	0.0	33	1.5	0	0.0	0	0.0	0	0.0	0.23	0.50
Dysaesthesia	1	0.0	0	0.0	0	0.0	0	0.0	0	0.0	1	0.0	0	0.0	0	0.0	>0.99	0.50
Dyspepsia	21	1.0	1	0.0	0	0.0	0	0.0	21	1.0	0	0.0	0	0.0	0	0.0	>0.99	>0.99
Dyspnoea	30	1.4	0	0.0	0	0.0	0	0.0	27	1.3	2	0.1	0	0.0	0	0.0	>0.99	0.25
Ejection fraction abnormal	0	0.0	1	0.0	0	0.0	0	0.0	0	0.0	0	0.0	0	0.0	0	0.0	>0.99	>0.99
Ejection fraction decreased	2	0.1	0	0.0	0	0.0	0	0.0	2	0.1	1	0.0	0	0.0	0	0.0	0.68	0.50
Embolism	0	0.0	0	0.0	1	0.0	0	0.0	0	0.0	0	0.0	0	0.0	0	0.0	>0.99	>0.99
Embolism venous	0	0.0	1	0.0	0	0.0	0	0.0	0	0.0	0	0.0	0	0.0	0	0.0	>0.99	>0.99
Extravasation	0	0.0	0	0.0	0	0.0	0	0.0	2	0.1	1	0.0	1	0.0	0	0.0	0.060	0.25
Femoral neck fracture	0	0.0	0	0.0	0	0.0	0	0.0	0	0.0	0	0.0	1	0.0	0	0.0	0.50	0.50
Fibromyalgia	3	0.1	1	0.0	0	0.0	0	0.0	2	0.1	2	0.1	0	0.0	0	0.0	>0.99	0.62
Gamma-glutamyltransferase	0	0.0	0	0.0	0	0.0	0	0.0	0	0.0	1	0.0	0	0.0	0	0.0	0.50	0.50

Glaucoma	0	0.0	0	0.0	0	0.0	0	0.0	0	0.0	1	0.0	0	0.0	0	0.0	0.50	0.50
Groin pain	0	0.0	0	0.0	0	0.0	0	0.0	1	0.0	1	0.0	0	0.0	0	0.0	0.25	0.50
Headache	21	1.0	0	0.0	0	0.0	0	0.0	22	1.0	1	0.0	0	0.0	0	0.0	0.76	0.50
Hot flush	250	11.4	12	0.5	0	0.0	0	0.0	212	9.8	6	0.3	1	0.0	0	0.0	0.066	0.36
Insomnia	18	0.8	0	0.0	0	0.0	0	0.0	19	0.9	2	0.1	0	0.0	0	0.0	0.63	0.25
Lacrimation increased	12	0.5	0	0.0	0	0.0	0	0.0	12	0.6	0	0.0	1	0.0	0	0.0	0.84	0.50
Liver function test abnormal	1	0.0	0	0.0	0	0.0	0	0.0	0	0.0	1	0.0	0	0.0	0	0.0	>0.99	0.50
Localised infection	1	0.0	0	0.0	0	0.0	0	0.0	1	0.0	1	0.0	0	0.0	0	0.0	0.62	0.50
Lymphoedema	77	3.5	2	0.1	0	0.0	0	0.0	93	4.3	2	0.1	0	0.0	0	0.0	0.19	>0.99
Memory impairment	20	0.9	0	0.0	0	0.0	0	0.0	13	0.6	1	0.0	0	0.0	0	0.0	0.39	0.50
Menopausal symptoms	16	0.7	1	0.0	0	0.0	0	0.0	9	0.4	0	0.0	0	0.0	0	0.0	0.17	>0.99
Menorrhagia	4	0.2	0	0.0	0	0.0	0	0.0	4	0.2	1	0.0	0	0.0	0	0.0	0.75	0.50
Menstrual disorder	0	0.0	0	0.0	0	0.0	0	0.0	1	0.0	1	0.0	0	0.0	0	0.0	0.25	0.50
Migraine	4	0.2	1	0.0	0	0.0	0	0.0	0	0.0	0	0.0	0	0.0	0	0.0	0.062	>0.99
Mood altered	4	0.2	1	0.0	0	0.0	0	0.0	2	0.1	0	0.0	0	0.0	0	0.0	0.45	>0.99
Muscular weakness	5	0.2	1	0.0	0	0.0	0	0.0	8	0.4	0	0.0	0	0.0	0	0.0	0.60	>0.99
Myalgia	27	1.2	3	0.1	0	0.0	0	0.0	28	1.3	2	0.1	0	0.0	0	0.0	>0.99	>0.99
Nail bed infection	1	0.0	1	0.0	0	0.0	0	0.0	0	0.0	0	0.0	0	0.0	0	0.0	0.50	>0.99
Neck pain	2	0.1	0	0.0	0	0.0	0	0.0	2	0.1	1	0.0	0	0.0	0	0.0	0.68	0.50
Neuralgia	8	0.4	0	0.0	0	0.0	0	0.0	9	0.4	2	0.1	0	0.0	0	0.0	0.50	0.25
Neuropathy peripheral	29	1.3	2	0.1	0	0.0	0	0.0	32	1.5	0	0.0	0	0.0	0	0.0	0.90	0.50
Non-cardiac chest pain	2	0.1	1	0.0	0	0.0	0	0.0	1	0.0	0	0.0	0	0.0	0	0.0	0.62	>0.99
Osteoarthritis	5	0.2	0	0.0	0	0.0	0	0.0	1	0.0	1	0.0	0	0.0	0	0.0	0.45	0.50
Osteoporotic fracture	0	0.0	1	0.0	0	0.0	0	0.0	0	0.0	0	0.0	0	0.0	0	0.0	>0.99	>0.99
Pain	21	1.0	2	0.1	0	0.0	0	0.0	21	1.0	0	0.0	1	0.0	0	0.0	>0.99	>0.99
Pain in extremity	43	2.0	2	0.1	0	0.0	0	0.0	57	2.6	2	0.1	0	0.0	0	0.0	0.16	>0.99
Periarthritis	3	0.1	0	0.0	1	0.0	0	0.0	3	0.1	0	0.0	0	0.0	0	0.0	>0.99	>0.99
Peripheral motor neuropathy	0	0.0	0	0.0	0	0.0	0	0.0	1	0.0	1	0.0	0	0.0	0	0.0	0.25	0.50
Peripheral sensory neuropathy	39	1.8	3	0.1	0	0.0	0	0.0	40	1.9	1	0.0	0	0.0	0	0.0	>0.99	0.62
Post procedural complication	0	0.0	1	0.0	0	0.0	0	0.0	0	0.0	0	0.0	0	0.0	0	0.0	>0.99	>0.99
Psychiatric symptom	0	0.0	1	0.0	0	0.0	0	0.0	0	0.0	0	0.0	0	0.0	0	0.0	>0.99	>0.99
Pulmonary embolism	0	0.0	1	0.0	0	0.0	0	0.0	0	0.0	1	0.0	0	0.0	0	0.0	>0.99	>0.99
Rehabilitation therapy	0	0.0	1	0.0	0	0.0	0	0.0	0	0.0	0	0.0	0	0.0	0	0.0	>0.99	>0.99
Suicide attempt	0	0.0	0	0.0	1	0.0	0	0.0	0	0.0	0	0.0	0	0.0	0	0.0	>0.99	>0.99
Toe amputation	0	0.0	0	0.0	0	0.0	0	0.0	0	0.0	1	0.0	0	0.0	0	0.0	0.50	0.50
Vein disorder	14	0.6	2	0.1	0	0.0	0	0.0	9	0.4	0	0.0	0	0.0	0	0.0	0.23	0.50
Ventricular tachycardia	0	0.0	0	0.0	0	0.0	0	0.0	0	0.0	1	0.0	0	0.0	0	0.0	0.50	0.50
Vision blurred	1	0.0	1	0.0	0	0.0	0	0.0	3	0.1	0	0.0	0	0.0	0	0.0	0.68	>0.99
Vulvovaginal dryness	18	0.8	0	0.0	0	0.0	0	0.0	13	0.6	1	0.0	0	0.0	0	0.0	0.60	0.50

a - Trend tests combine grade 3, 4 and 5 adverse events

b - Free text other adverse events have been MedDRA coded. Preferred terms are included here

Table A14 - Frequency of late adverse events by CMF/X

Analysis of late safety compared all signs and symptoms reported at or after 12 months from randomisation and include all patients randomised who were followed-up for at least 9 months post-randomisation and who received at least one cycle of allocated chemotherapy. Late safety analysis was censored at the point of disease recurrence or second primary cancer.

Adverse events are included if occurring either in >10% of patients at grade ≤2, or at grade ≥3 by any patient

	CMF (N = 2176)								X (N = 2172)								Trend test: p-value[a]	Grade ≥3: Fisher's exact p-value
	Grade 1 + 2		Grade 3		Grade 4		Grade 5		Grade 1 + 2		Grade 3		Grade 4		Grade 5			
	n	%	n	%	n	%	n	%	n	%	n	%	n	%	n	%		
**Pre-specified CRF adverse events**																		
Fatigue	781	35.9	21	1.0	2	0.1	0	0.0	758	34.9	20	0.9	3	0.1	0	0.0	0.14	>0.99
Hand-foot syndrome	89	4.1	1	0.0	0	0.0	0	0.0	208	9.6	3	0.1	0	0.0	0	0.0	<0.0001	0.37

	CMF (N = 2176)								X (N = 2172)								Any grade: Fisher's exact p-value	Grade ≥3: Fisher's exact p-value
	Grade 1 + 2		Grade 3		Grade 4		Grade 5		Grade 1 + 2		Grade 3		Grade 4		Grade 5			
	n	%	n	%	n	%	n	%	n	%	n	%	n	%	n	%		
**Other MedDRA coded adverse events[b]**																		
Abnormal weight gain	25	1.1	3	0.1	0	0.0	0	0.0	18	0.8	0	0.0	0	0.0	0	0.0	0.18	0.25
Ageusia	1	0.0	1	0.0	0	0.0	0	0.0	2	0.1	0	0.0	0	0.0	0	0.0	>0.99	>0.99
Anxiety	20	0.9	1	0.0	0	0.0	0	0.0	12	0.6	1	0.0	0	0.0	0	0.0	0.23	>0.99
Arthralgia	263	12.1	13	0.6	0	0.0	0	0.0	200	9.2	6	0.3	1	0.0	0	0.0	0.0010	0.26
Arthritis	9	0.4	1	0.0	0	0.0	0	0.0	7	0.3	0	0.0	0	0.0	0	0.0	0.63	>0.99
Arthropathy	1	0.0	0	0.0	0	0.0	0	0.0	1	0.0	1	0.0	0	0.0	0	0.0	0.62	0.50
Asthenia	4	0.2	0	0.0	0	0.0	0	0.0	3	0.1	1	0.0	0	0.0	0	0.0	>0.99	0.50
Atrial fibrillation	0	0.0	0	0.0	0	0.0	0	0.0	1	0.0	1	0.0	0	0.0	0	0.0	0.25	0.50
Atrophic vulvovaginitis	0	0.0	0	0.0	0	0.0	0	0.0	1	0.0	1	0.0	0	0.0	0	0.0	0.25	0.50
Back pain	38	1.7	1	0.0	0	0.0	0	0.0	35	1.6	1	0.0	0	0.0	0	0.0	0.82	>0.99
Balance disorder	1	0.0	0	0.0	0	0.0	0	0.0	0	0.0	1	0.0	0	0.0	0	0.0	>0.99	0.50
Bone pain	15	0.7	1	0.0	0	0.0	0	0.0	4	0.2	1	0.0	0	0.0	0	0.0	0.026	>0.99
Cardiac disorder	0	0.0	1	0.0	0	0.0	0	0.0	1	0.0	0	0.0	0	0.0	0	0.0	>0.99	>0.99
Cardiac failure	0	0.0	1	0.0	0	0.0	0	0.0	1	0.0	1	0.0	0	0.0	0	0.0	0.62	>0.99
Cardio-respiratory arrest	0	0.0	0	0.0	1	0.0	0	0.0	0	0.0	0	0.0	0	0.0	0	0.0	>0.99	>0.99
Cardiomyopathy	2	0.1	1	0.0	1	0.0	0	0.0	0	0.0	0	0.0	0	0.0	0	0.0	0.12	0.50
Carpal tunnel syndrome	7	0.3	0	0.0	0	0.0	0	0.0	7	0.3	1	0.0	0	0.0	0	0.0	0.80	0.50
Cataract	1	0.0	3	0.1	0	0.0	0	0.0	1	0.0	0	0.0	0	0.0	0	0.0	0.37	0.25
Catheter related infection	0	0.0	1	0.0	0	0.0	0	0.0	0	0.0	0	0.0	0	0.0	0	0.0	>0.99	>0.99
Cerebrovascular accident	1	0.0	0	0.0	0	0.0	0	0.0	0	0.0	0	0.0	1	0.0	0	0.0	>0.99	0.50
Deep vein thrombosis	2	0.1	1	0.0	0	0.0	0	0.0	0	0.0	0	0.0	0	0.0	0	0.0	0.25	>0.99
Dementia	1	0.0	1	0.0	0	0.0	0	0.0	0	0.0	0	0.0	0	0.0	0	0.0	0.50	>0.99
Depression	20	0.9	4	0.2	1	0.0	0	0.0	26	1.2	1	0.0	1	0.0	0	0.0	0.68	0.45
Disturbance in attention	4	0.2	0	0.0	0	0.0	0	0.0	8	0.4	1	0.0	0	0.0	0	0.0	0.18	0.50
Dry mouth	36	1.7	2	0.1	0	0.0	0	0.0	19	0.9	0	0.0	0	0.0	0	0.0	0.016	0.50
Dysaesthesia	1	0.0	0	0.0	0	0.0	0	0.0	0	0.0	1	0.0	0	0.0	0	0.0	>0.99	0.50
Dyspepsia	26	1.2	1	0.0	0	0.0	0	0.0	16	0.7	0	0.0	0	0.0	0	0.0	0.12	>0.99
Dyspnoea	28	1.3	1	0.0	0	0.0	0	0.0	29	1.3	1	0.0	0	0.0	0	0.0	0.90	>0.99
Ejection fraction abnormal	0	0.0	0	0.0	0	0.0	0	0.0	0	0.0	1	0.0	0	0.0	0	0.0	0.50	0.50
Ejection fraction decreased	1	0.0	0	0.0	0	0.0	0	0.0	3	0.1	1	0.0	0	0.0	0	0.0	0.22	0.50
Embolism	0	0.0	0	0.0	0	0.0	0	0.0	0	0.0	0	0.0	1	0.0	0	0.0	0.50	0.50
Embolism venous	0	0.0	0	0.0	0	0.0	0	0.0	0	0.0	1	0.0	0	0.0	0	0.0	0.50	0.50
Extravasation	1	0.0	0	0.0	0	0.0	0	0.0	1	0.0	1	0.0	1	0.0	0	0.0	0.37	0.25
Femoral neck fracture	0	0.0	0	0.0	0	0.0	0	0.0	0	0.0	0	0.0	1	0.0	0	0.0	0.50	0.50
Fibromyalgia	1	0.0	3	0.1	0	0.0	0	0.0	4	0.2	0	0.0	0	0.0	0	0.0	>0.99	0.25
Gamma-glutamyltransferase	0	0.0	0	0.0	0	0.0	0	0.0	0	0.0	1	0.0	0	0.0	0	0.0	0.50	0.50
Glaucoma	0	0.0	0	0.0	0	0.0	0	0.0	0	0.0	1	0.0	0	0.0	0	0.0	0.50	0.50

Groin pain	0	0.0	1	0.0	0	0.0	0	0.0	1	0.0	0	0.0	0	0.0	0	0.0	>0.99	>0.99
Headache	26	1.2	0	0.0	0	0.0	0	0.0	17	0.8	1	0.0	0	0.0	0	0.0	0.29	0.50
Hot flush	253	11.6	7	0.3	0	0.0	0	0.0	209	9.6	11	0.5	1	0.0	0	0.0	0.066	0.26
Insomnia	25	1.1	1	0.0	0	0.0	0	0.0	12	0.6	1	0.0	0	0.0	0	0.0	0.052	>0.99
Lacrimation increased	7	0.3	0	0.0	1	0.0	0	0.0	17	0.8	0	0.0	0	0.0	0	0.0	0.074	>0.99
Liver function test abnormal	0	0.0	1	0.0	0	0.0	0	0.0	1	0.0	0	0.0	0	0.0	0	0.0	>0.99	>0.99
Localised infection	1	0.0	1	0.0	0	0.0	0	0.0	1	0.0	0	0.0	0	0.0	0	0.0	>0.99	>0.99
Lymphoedema	90	4.1	1	0.0	0	0.0	0	0.0	80	3.7	3	0.1	0	0.0	0	0.0	0.59	0.37
Memory impairment	13	0.6	0	0.0	0	0.0	0	0.0	20	0.9	1	0.0	0	0.0	0	0.0	0.17	0.50
Menopausal symptoms	17	0.8	0	0.0	0	0.0	0	0.0	8	0.4	1	0.0	0	0.0	0	0.0	0.17	0.50
Menorrhagia	3	0.1	0	0.0	0	0.0	0	0.0	5	0.2	1	0.0	0	0.0	0	0.0	0.34	0.50
Menstrual disorder	0	0.0	1	0.0	0	0.0	0	0.0	1	0.0	0	0.0	0	0.0	0	0.0	>0.99	>0.99
Migraine	0	0.0	0	0.0	0	0.0	0	0.0	4	0.2	1	0.0	0	0.0	0	0.0	0.031	0.50
Mood altered	5	0.2	0	0.0	0	0.0	0	0.0	1	0.0	1	0.0	0	0.0	0	0.0	0.45	0.50
Muscular weakness	5	0.2	0	0.0	0	0.0	0	0.0	8	0.4	1	0.0	0	0.0	0	0.0	0.30	0.50
Myalgia	27	1.2	3	0.1	0	0.0	0	0.0	28	1.3	2	0.1	0	0.0	0	0.0	>0.99	>0.99
Nail bed infection	1	0.0	0	0.0	0	0.0	0	0.0	0	0.0	1	0.0	0	0.0	0	0.0	>0.99	0.50
Neck pain	1	0.0	0	0.0	0	0.0	0	0.0	3	0.1	1	0.0	0	0.0	0	0.0	0.22	0.50
Neuralgia	9	0.4	0	0.0	0	0.0	0	0.0	8	0.4	2	0.1	0	0.0	0	0.0	0.82	0.25
Neuropathy peripheral	24	1.1	1	0.0	0	0.0	0	0.0	37	1.7	1	0.0	0	0.0	0	0.0	0.10	>0.99
Non-cardiac chest pain	2	0.1	0	0.0	0	0.0	0	0.0	1	0.0	1	0.0	0	0.0	0	0.0	>0.99	0.50
Osteoarthritis	2	0.1	1	0.0	0	0.0	0	0.0	4	0.2	0	0.0	0	0.0	0	0.0	0.73	>0.99
Osteoporotic fracture	0	0.0	0	0.0	0	0.0	0	0.0	0	0.0	1	0.0	0	0.0	0	0.0	0.50	0.50
Pain	22	1.0	2	0.1	0	0.0	0	0.0	20	0.9	0	0.0	1	0.0	0	0.0	0.76	>0.99
Pain in extremity	54	2.5	2	0.1	0	0.0	0	0.0	46	2.1	2	0.1	0	0.0	0	0.0	0.49	>0.99
Periarthritis	1	0.0	0	0.0	1	0.0	0	0.0	5	0.2	0	0.0	0	0.0	0	0.0	0.29	>0.99
Peripheral motor neuropathy	0	0.0	1	0.0	0	0.0	0	0.0	1	0.0	0	0.0	0	0.0	0	0.0	>0.99	>0.99
Peripheral sensory neuropathy	35	1.6	2	0.1	0	0.0	0	0.0	44	2.0	2	0.1	0	0.0	0	0.0	0.32	>0.99
Post procedural complication	0	0.0	0	0.0	0	0.0	0	0.0	0	0.0	1	0.0	0	0.0	0	0.0	0.50	0.50
Psychiatric symptom	0	0.0	1	0.0	0	0.0	0	0.0	0	0.0	0	0.0	0	0.0	0	0.0	>0.99	>0.99
Pulmonary embolism	0	0.0	1	0.0	0	0.0	0	0.0	0	0.0	1	0.0	0	0.0	0	0.0	>0.99	>0.99
Rehabilitation therapy	0	0.0	1	0.0	0	0.0	0	0.0	0	0.0	0	0.0	0	0.0	0	0.0	>0.99	>0.99
Suicide attempt	0	0.0	0	0.0	1	0.0	0	0.0	0	0.0	0	0.0	0	0.0	0	0.0	>0.99	>0.99
Toe amputation	0	0.0	0	0.0	0	0.0	0	0.0	0	0.0	1	0.0	0	0.0	0	0.0	0.50	0.50
Vein disorder	13	0.6	1	0.0	0	0.0	0	0.0	10	0.5	1	0.0	0	0.0	0	0.0	0.69	>0.99
Ventricular tachycardia	0	0.0	0	0.0	0	0.0	0	0.0	0	0.0	1	0.0	0	0.0	0	0.0	0.50	0.50
Vision blurred	2	0.1	1	0.0	0	0.0	0	0.0	2	0.1	0	0.0	0	0.0	0	0.0	>0.99	>0.99
Vulvovaginal dryness	16	0.7	1	0.0	0	0.0	0	0.0	15	0.7	0	0.0	0	0.0	0	0.0	0.86	>0.99

a - Trend tests combine grade 3, 4 and 5 adverse events

b - Free text other adverse events have been MedDRA coded. Preferred terms are included here

TACT2 patient reported toxicities questionnaire

TACT 2 Toxicities Questionnaire

We are interested to discover how the symptoms / side effects listed below and on the next page have affected you. We would like to know this whether or not you think any symptoms / side effects were due to breast cancer or to your treatment.

On this page, rate **how distressed you were** by each of the symptoms / side effects shown below, e.g. if you experienced nausea and found it a little upsetting then circle '2'.

During the past 4 weeks:

How much did the symptom / side effects cause you distress?

	Did not suffer from	Not at All	A Little	Quite a Bit	Very Much
Nausea	0	1	2	3	4
Vomiting	0	1	2	3	4
Dry, flaky or sensitive skin	0	1	2	3	4
Tingling, numb, sore or red hands &/or feet	0	1	2	3	4
Tiredness	0	1	2	3	4
Diarrhoea	0	1	2	3	4
Constipation	0	1	2	3	4
Sore mouth	0	1	2	3	4
Mouth ulcers	0	1	2	3	4
Breathlessness	0	1	2	3	4
Lack of appetite	0	1	2	3	4
Pain in bones, muscles and/or joints	0	1	2	3	4
Painful or gritty eyes	0	1	2	3	4
Weight gain	0	1	2	3	4

On this page please rate the extent to which the symptoms / side effects **affected your day to day activities** (quality of life) by circling the appropriate number, e.g. if you experienced nausea but found that it did not interfere with your day to day activities, then circle '1' for 'not at all'.

During the past 4 weeks:

How much did the symptom / side effect interfere with your day to day activities?

	Did not suffer from	Not at All	A Little	Quite a Bit	Very Much
Nausea	0	1	2	3	4
Vomiting	0	1	2	3	4
Dry, flaky or sensitive skin	0	1	2	3	4
Tingling, numb, sore or red hands &/or feet	0	1	2	3	4
Tiredness	0	1	2	3	4
Diarrhoea	0	1	2	3	4
Constipation	0	1	2	3	4
Sore mouth	0	1	2	3	4
Mouth ulcers	0	1	2	3	4
Breathlessness	0	1	2	3	4
Lack of appetite	0	1	2	3	4
Pain in bones, muscles and/or joints	0	1	2	3	4
Painful or gritty eyes	0	1	2	3	4

We are interested in whether you gain weight during your treatment. Please circle your answer to this question.

	Did not suffer from	Not at All	A Little	Quite a Bit	Very Much
Since your chemotherapy treatment started, have you gained weight?	0	1	2	3	4

Table A15 - Cross-sectional analyses at the end of cycle 4 by E/aE - Distress caused by toxicity

	E		aE		Total		p-value - Trend test$	p-value - Fisher's exact&
	n	%	n	%	n	%		
**Nausea**								
Did not suffer	126	24.6%	111	23.1%	237	23.9%	0.79	0.94
Not at all	56	10.9%	52	10.8%	108	10.9%		
A little	183	35.7%	178	37.1%	361	36.4%		
Quite a bit	104	20.3%	100	20.8%	204	20.6%		
Very much	43	8.4%	39	8.1%	82	8.3%		
**Vomiting**								
Did not suffer	337	65.8%	339	71.2%	676	68.4%	0.056	0.28
Not at all	60	11.7%	56	11.8%	116	11.7%		
A little	80	15.6%	57	12.0%	137	13.9%		
Quite a bit	26	5.1%	17	3.6%	43	4.4%		
Very much	9	1.8%	7	1.5%	16	1.6%		
**Dry/flaky/sensitive skin**								
Did not suffer	244	47.7%	204	42.7%	448	45.3%	0.0032	0.0035
Not at all	101	19.8%	81	16.9%	182	18.4%		
A little	134	26.2%	137	28.7%	271	27.4%		
Quite a bit	21	4.1%	45	9.4%	66	6.7%		
Very much	11	2.2%	11	2.3%	22	2.2%		
**Tingling/numb/sore hands or feet**								
Did not suffer	329	64.5%	295	61.8%	624	63.2%	0.0026	0.0011
Not at all	74	14.5%	58	12.2%	132	13.4%		
A little	79	15.5%	70	14.7%	149	15.1%		
Quite a bit	23	4.5%	38	8.0%	61	6.2%		
Very much	5	1.0%	16	3.4%	21	2.1%		
**Tiredness**								
Did not suffer	17	3.3%	20	4.2%	37	3.7%	0.18	0.18
Not at all	53	10.4%	60	12.6%	113	11.4%		
A little	178	34.9%	173	36.3%	351	35.6%		
Quite a bit	181	35.5%	151	31.7%	332	33.6%		
Very much	81	15.9%	73	15.3%	154	15.6%		
**Diarrhoea**								
Did not suffer	284	55.6%	280	58.3%	564	56.9%	0.92	0.28
Not at all	79	15.5%	68	14.2%	147	14.8%		
A little	115	22.5%	92	19.2%	207	20.9%		
Quite a bit	18	3.5%	30	6.3%	48	4.8%		
Very much	15	2.9%	10	2.1%	25	2.5%		
**Constipation**								
Did not suffer	145	28.3%	128	26.8%	273	27.6%	0.76	>0.99
Not at all	76	14.8%	65	13.6%	141	14.2%		
A little	159	31.0%	161	33.8%	320	32.3%		
Quite a bit	88	17.2%	84	17.6%	172	17.4%		
Very much	45	8.8%	39	8.2%	84	8.5%		
**Sore mouth**								
Did not suffer	174	34.1%	156	32.6%	330	33.3%	0.68	0.88
Not at all	72	14.1%	62	12.9%	134	13.5%		
A little	142	27.8%	148	30.9%	290	29.3%		
Quite a bit	74	14.5%	65	13.6%	139	14.0%		
Very much	49	9.6%	48	10.0%	97	9.8%		
**Mouth ulcers**								
Did not suffer	247	48.2%	239	49.7%	486	48.9%	0.42	0.47
Not at all	86	16.8%	63	13.1%	149	15.0%		
A little	108	21.1%	104	21.6%	212	21.3%		
Quite a bit	40	7.8%	43	8.9%	83	8.4%		

	E		aE		Total		p-value - Trend test$	p-value - Fisher's exact &
	n	%	n	%	n	%		
Very much	31	6.1%	32	6.7%	63	6.3%		
**Breathlessness**								
Did not suffer	217	42.4%	200	41.8%	417	42.1%	0.29	0.78
Not at all	91	17.8%	68	14.2%	159	16.1%		
A little	140	27.3%	147	30.8%	287	29.0%		
Quite a bit	47	9.2%	44	9.2%	91	9.2%		
Very much	17	3.3%	19	4.0%	36	3.6%		
**Lack of appetite**								
Did not suffer	183	35.7%	178	37.3%	361	36.5%	0.36	0.37
Not at all	112	21.8%	95	19.9%	207	20.9%		
A little	146	28.5%	127	26.6%	273	27.6%		
Quite a bit	55	10.7%	49	10.3%	104	10.5%		
Very much	17	3.3%	28	5.9%	45	4.5%		
**Pain in bones/muscles/joints**								
Did not suffer	224	43.8%	194	40.4%	418	42.2%	0.25	0.43
Not at all	73	14.3%	68	14.2%	141	14.2%		
A little	139	27.2%	138	28.7%	277	28.0%		
Quite a bit	51	10.0%	54	11.3%	105	10.6%		
Very much	24	4.7%	26	5.4%	50	5.0%		
**Painful/gritty eyes**								
Did not suffer	151	29.8%	176	36.8%	327	33.2%	0.71	>0.99
Not at all	87	17.2%	64	13.4%	151	15.3%		
A little	170	33.5%	145	30.3%	315	32.0%		
Quite a bit	68	13.4%	58	12.1%	126	12.8%		
Very much	31	6.1%	35	7.3%	66	6.7%		
**Weight gain**								
Did not suffer	173	33.9%	219	45.8%	392	39.6%	<0.0001	0.0016
Not at all	91	17.8%	100	20.9%	191	19.3%		
A little	148	29.0%	102	21.3%	250	25.3%		
Quite a bit	61	11.9%	36	7.5%	97	9.8%		
Very much	38	7.4%	21	4.4%	59	6.0%		

$ - Trend test with "Did not suffer" and "Not at all" combined into one group
& - Fisher's exact test test comparing the proportion reporting "Quite a bit" or "Very much"

Table A16 - Cross-sectional analyses at the end of cycle 4 by E/aE - Daily interference caused by toxicity

	E		aE		Total		p-value - Trend test$	p-value - Fisher's exact &
	n	%	n	%	n	%		
**Nausea**								
Did not suffer	130	25.4%	117	24.4%	247	24.9%	0.80	0.87
Not at all	139	27.1%	134	28.0%	273	27.5%		
A little	143	27.9%	132	27.6%	275	27.7%		
Quite a bit	74	14.5%	68	14.2%	142	14.3%		
Very much	26	5.1%	28	5.8%	54	5.4%		
**Vomiting**								
Did not suffer	327	64.0%	338	70.4%	665	67.1%	0.035	0.14
Not at all	99	19.4%	89	18.5%	188	19.0%		
A little	60	11.7%	39	8.1%	99	10.0%		
Quite a bit	18	3.5%	6	1.3%	24	2.4%		
Very much	7	1.4%	8	1.7%	15	1.5%		
**Dry/flaky/sensitive skin**								
Did not suffer	253	49.5%	205	42.9%	458	46.3%	0.14	0.44
Not at all	199	38.9%	205	42.9%	404	40.8%		

	E		aE		Total		p-value - Trend test$	p-value - Fisher's exact &
	n	%	n	%	n	%		
A little	47	9.2%	52	10.9%	99	10.0%		
Quite a bit	9	1.8%	9	1.9%	18	1.8%		
Very much	3	0.6%	7	1.5%	10	1.0%		
**Tingling/numb/sore hands or feet**								
Did not suffer	321	62.9%	283	59.3%	604	61.2%	0.00083	0.031
Not at all	125	24.5%	98	20.5%	223	22.6%		
A little	42	8.2%	59	12.4%	101	10.2%		
Quite a bit	18	3.5%	24	5.0%	42	4.3%		
Very much	4	0.8%	13	2.7%	17	1.7%		
**Tiredness**								
Did not suffer	17	3.3%	17	3.6%	34	3.4%	0.23	0.41
Not at all	78	15.3%	62	13.0%	140	14.2%		
A little	189	37.0%	173	36.3%	362	36.6%		
Quite a bit	152	29.7%	143	30.0%	295	29.9%		
Very much	75	14.7%	82	17.2%	157	15.9%		
**Diarrhoea**								
Did not suffer	285	55.8%	280	58.5%	565	57.1%	0.94	0.68
Not at all	117	22.9%	98	20.5%	215	21.7%		
A little	82	16.0%	72	15.0%	154	15.6%		
Quite a bit	13	2.5%	17	3.5%	30	3.0%		
Very much	14	2.7%	12	2.5%	26	2.6%		
**Constipation**								
Did not suffer	153	29.9%	130	27.1%	283	28.6%	0.65	>0.99
Not at all	179	35.0%	167	34.9%	346	34.9%		
A little	109	21.3%	116	24.2%	225	22.7%		
Quite a bit	42	8.2%	40	8.4%	82	8.3%		
Very much	29	5.7%	26	5.4%	55	5.5%		
**Sore mouth**								
Did not suffer	192	37.6%	154	32.2%	346	35.0%	0.58	0.85
Not at all	155	30.3%	161	33.7%	316	32.0%		
A little	101	19.8%	102	21.3%	203	20.5%		
Quite a bit	32	6.3%	29	6.1%	61	6.2%		
Very much	31	6.1%	32	6.7%	63	6.4%		
**Mouth ulcers**								
Did not suffer	258	50.5%	232	48.8%	490	49.7%	0.84	0.73
Not at all	142	27.8%	132	27.8%	274	27.8%		
A little	65	12.7%	72	15.2%	137	13.9%		
Quite a bit	21	4.1%	15	3.2%	36	3.7%		
Very much	25	4.9%	24	5.1%	49	5.0%		
**Breathlessness**								
Did not suffer	231	45.2%	196	41.2%	427	43.3%	0.40	0.57
Not at all	86	16.8%	92	19.3%	178	18.0%		
A little	132	25.8%	124	26.1%	256	25.9%		
Quite a bit	48	9.4%	44	9.2%	92	9.3%		
Very much	14	2.7%	20	4.2%	34	3.4%		
**Lack of appetite**								
Did not suffer	191	37.3%	191	39.7%	382	38.5%	0.15	0.083
Not at all	173	33.8%	144	29.9%	317	31.9%		
A little	101	19.7%	85	17.7%	186	18.7%		
Quite a bit	31	6.1%	35	7.3%	66	6.6%		
Very much	16	3.1%	26	5.4%	42	4.2%		
**Pain in bones/muscles/joints**								
Did not suffer	234	45.8%	198	41.3%	432	43.6%	0.34	0.37

	E		aE		Total		p-value - Trend test$	p-value - Fisher's exact &
	n	%	n	%	n	%		
Not at all	119	23.3%	122	25.5%	241	24.3%		
A little	104	20.4%	99	20.7%	203	20.5%		
Quite a bit	35	6.8%	39	8.1%	74	7.5%		
Very much	19	3.7%	21	4.4%	40	4.0%		
**Painful/gritty eyes**								
Did not suffer	169	33.0%	182	37.9%	351	35.4%	0.71	>0.99
Not at all	152	29.7%	129	26.9%	281	28.3%		
A little	130	25.4%	111	23.1%	241	24.3%		
Quite a bit	41	8.0%	39	8.1%	80	8.1%		
Very much	20	3.9%	19	4.0%	39	3.9%		
**Weight gain**								
Did not suffer	59	11.6%	83	17.4%	142	14.4%	<0.0001	0.0037
Not at all	137	26.9%	180	37.7%	317	32.2%		
A little	210	41.3%	151	31.7%	361	36.6%		
Quite a bit	73	14.3%	46	9.6%	119	12.1%		
Very much	30	5.9%	17	3.6%	47	4.8%		

$ - Trend test with "Did not suffer" and "Not at all" combined into one group
& - Fisher's exact test comparing the proportion reporting "Quite a bit" or "Very much"

Table A17 - Cross-sectional analyses at the end of cycle 8 by CMF/X - Distress caused by toxicity

	CMF		X		Total		p-value - Trend test$	p-value - Fisher's exact&
	n	%	n	%	n	%		
**Nausea**								
Did not suffer	151	32.6%	214	43.7%	365	38.3%	0.034	0.32
Not at all	65	14.0%	66	13.5%	131	13.7%		
A little	173	37.4%	143	29.2%	316	33.2%		
Quite a bit	63	13.6%	47	9.6%	110	11.5%		
Very much	11	2.4%	20	4.1%	31	3.3%		
**Vomiting**								
Did not suffer	320	69.0%	371	75.7%	691	72.4%	0.048	0.75
Not at all	58	12.5%	63	12.9%	121	12.7%		
A little	65	14.0%	36	7.3%	101	10.6%		
Quite a bit	14	3.0%	11	2.2%	25	2.6%		
Very much	7	1.5%	9	1.8%	16	1.7%		
**Dry/flaky/sensitive skin**								
Did not suffer	197	42.8%	163	33.3%	360	37.9%	<0.0001	<0.0001
Not at all	94	20.4%	53	10.8%	147	15.5%		
A little	127	27.6%	141	28.8%	268	28.2%		
Quite a bit	37	8.0%	91	18.6%	128	13.5%		
Very much	5	1.1%	41	8.4%	46	4.8%		
**Tingling/numb/sore hands or feet**								
Did not suffer	228	49.2%	92	18.7%	320	33.5%	<0.0001	<0.0001
Not at all	85	18.4%	30	6.1%	115	12.1%		
A little	98	21.2%	121	24.6%	219	23.0%		
Quite a bit	37	8.0%	142	28.9%	179	18.8%		
Very much	15	3.2%	106	21.6%	121	12.7%		
**Tiredness**								
Did not suffer	21	4.5%	40	8.2%	61	6.4%	<0.0001	<0.0001
Not at all	33	7.1%	45	9.2%	78	8.2%		
A little	162	35.1%	214	43.9%	376	39.6%		
Quite a bit	160	34.6%	128	26.2%	288	30.3%		
Very much	86	18.6%	61	12.5%	147	15.5%		
**Diarrhoea**								
Did not suffer	190	40.9%	222	45.2%	412	43.1%	0.93	0.79
Not at all	67	14.4%	58	11.8%	125	13.1%		

	CMF		X		Total		p-value - Trend test$	p-value - Fisher's exact&
	n	%	n	%	n	%		
A little	137	29.5%	133	27.1%	270	28.3%		
Quite a bit	47	10.1%	47	9.6%	94	9.8%		
Very much	23	5.0%	31	6.3%	54	5.7%		
**Constipation**								
Did not suffer	156	33.8%	292	59.8%	448	47.2%	<0.0001	<0.0001
Not at all	73	15.8%	74	15.2%	147	15.5%		
A little	158	34.2%	94	19.3%	252	26.5%		
Quite a bit	48	10.4%	12	2.5%	60	6.3%		
Very much	27	5.8%	16	3.3%	43	4.5%		
**Sore mouth**								
Did not suffer	149	32.2%	249	50.7%	398	41.7%	<0.0001	<0.0001
Not at all	69	14.9%	69	14.1%	138	14.5%		
A little	139	30.0%	123	25.1%	262	27.5%		
Quite a bit	59	12.7%	32	6.5%	91	9.5%		
Very much	47	10.2%	18	3.7%	65	6.8%		
**Mouth ulcers**								
Did not suffer	215	46.6%	324	66.1%	539	56.7%	<0.0001	<0.0001
Not at all	77	16.7%	64	13.1%	141	14.8%		
A little	98	21.3%	72	14.7%	170	17.9%		
Quite a bit	34	7.4%	19	3.9%	53	5.6%		
Very much	37	8.0%	11	2.2%	48	5.0%		
**Breathlessness**								
Did not suffer	143	30.8%	238	48.8%	381	40.0%	<0.0001	0.0020
Not at all	62	13.3%	72	14.8%	134	14.1%		
A little	167	35.9%	117	24.0%	284	29.8%		
Quite a bit	69	14.8%	42	8.6%	111	11.6%		
Very much	24	5.2%	19	3.9%	43	4.5%		
**Lack of appetite**								
Did not suffer	184	39.7%	238	48.7%	422	44.3%	0.036	0.24
Not at all	84	18.1%	85	17.4%	169	17.8%		
A little	132	28.5%	112	22.9%	244	25.6%		
Quite a bit	41	8.9%	33	6.7%	74	7.8%		
Very much	22	4.8%	21	4.3%	43	4.5%		
**Pain in bones/muscles/joints**								
Did not suffer	173	37.4%	200	40.8%	373	39.2%	0.53	0.62
Not at all	72	15.6%	70	14.3%	142	14.9%		
A little	128	27.7%	132	26.9%	260	27.3%		
Quite a bit	54	11.7%	53	10.8%	107	11.2%		
Very much	35	7.6%	35	7.1%	70	7.4%		
**Painful/gritty eyes**								
Did not suffer	167	36.1%	227	46.2%	394	41.3%	0.046	0.48
Not at all	74	16.0%	71	14.5%	145	15.2%		
A little	143	31.0%	119	24.2%	262	27.5%		
Quite a bit	51	11.0%	50	10.2%	101	10.6%		
Very much	27	5.8%	24	4.9%	51	5.4%		
**Weight gain**								
Did not suffer	136	29.5%	181	36.8%	317	33.3%	0.018	0.028
Not at all	84	18.2%	80	16.3%	164	17.2%		
A little	125	27.1%	137	27.8%	262	27.5%		
Quite a bit	63	13.7%	56	11.4%	119	12.5%		
Very much	53	11.5%	38	7.7%	91	9.5%		

$ - Trend test with "Did not suffer" and "Not at all" combined into one group
& - Fisher's exact test comparing the proportion reporting "Quite a bit" or "Very much"

Table A18 - Cross-sectional analyses at the end of cycle 8 by CMF/X - Daily interference caused by toxicity

	CMF		X		Total		p-value - Trend test$	p-value - Fisher's exact&
	n	%	n	%	n	%		
**Nausea**								
Did not suffer	165	35.6%	225	45.6%	390	40.8%	0.0067	0.13
Not at all	130	28.1%	140	28.4%	270	28.2%		
A little	122	26.3%	93	18.9%	215	22.5%		
Quite a bit	37	8.0%	22	4.5%	59	6.2%		
Very much	9	1.9%	13	2.6%	22	2.3%		
**Vomiting**								
Did not suffer	319	68.8%	381	77.6%	700	73.3%	0.12	0.71
Not at all	84	18.1%	76	15.5%	160	16.8%		
A little	48	10.3%	18	3.7%	66	6.9%		
Quite a bit	8	1.7%	7	1.4%	15	1.6%		
Very much	5	1.1%	9	1.8%	14	1.5%		
**Dry/flaky/sensitive skin**								
Did not suffer	212	45.9%	188	38.3%	400	42.0%	<0.0001	<0.0001
Not at all	185	40.0%	140	28.5%	325	34.1%		
A little	53	11.5%	102	20.8%	155	16.3%		
Quite a bit	10	2.2%	33	6.7%	43	4.5%		
Very much	2	0.4%	28	5.7%	30	3.1%		
**Tingling/numb/sore hands or feet**								
Did not suffer	235	51.1%	100	20.3%	335	35.2%	<0.0001	<0.0001
Not at all	126	27.4%	74	15.0%	200	21.0%		
A little	71	15.4%	148	30.1%	219	23.0%		
Quite a bit	18	3.9%	96	19.5%	114	12.0%		
Very much	10	2.2%	74	15.0%	84	8.8%		
**Tiredness**								
Did not suffer	25	5.4%	51	10.5%	76	8.0%	<0.0001	<0.0001
Not at all	49	10.7%	60	12.3%	109	11.5%		
A little	169	36.7%	215	44.1%	384	40.5%		
Quite a bit	136	29.6%	105	21.5%	241	25.4%		
Very much	81	17.6%	57	11.7%	138	14.6%		
**Diarrhoea**								
Did not suffer	195	42.1%	234	47.7%	429	45.0%	0.67	0.92
Not at all	107	23.1%	105	21.4%	212	22.2%		
A little	106	22.9%	95	19.3%	201	21.1%		
Quite a bit	38	8.2%	30	6.1%	68	7.1%		
Very much	17	3.7%	27	5.5%	44	4.6%		
**Constipation**								
Did not suffer	167	36.1%	305	62.0%	472	49.4%	<0.0001	<0.0001
Not at all	147	31.7%	123	25.0%	270	28.3%		
A little	102	22.0%	49	10.0%	151	15.8%		
Quite a bit	30	6.5%	6	1.2%	36	3.8%		
Very much	17	3.7%	9	1.8%	26	2.7%		
**Sore mouth**								
Did not suffer	163	35.3%	257	52.2%	420	44.0%	<0.0001	0.00020
Not at all	136	29.4%	135	27.4%	271	28.4%		
A little	97	21.0%	66	13.4%	163	17.1%		
Quite a bit	33	7.1%	21	4.3%	54	5.7%		
Very much	33	7.1%	13	2.6%	46	4.8%		
**Mouth ulcers**								
Did not suffer	224	48.4%	321	65.2%	545	57.1%	<0.0001	<0.0001
Not at all	114	24.6%	96	19.5%	210	22.0%		
A little	70	15.1%	55	11.2%	125	13.1%		

	CMF		X		Total		p-value - Trend test$	p-value - Fisher's exact&
	n	%	n	%	n	%		
Quite a bit	25	5.4%	12	2.4%	37	3.9%		
Very much	30	6.5%	8	1.6%	38	4.0%		
**Breathlessness**								
Did not suffer	141	30.4%	247	50.5%	388	40.7%	<0.0001	<0.0001
Not at all	92	19.8%	85	17.4%	177	18.6%		
A little	140	30.2%	103	21.1%	243	25.5%		
Quite a bit	70	15.1%	29	5.9%	99	10.4%		
Very much	21	4.5%	25	5.1%	46	4.8%		
**Lack of appetite**								
Did not suffer	202	43.7%	249	50.7%	451	47.3%	0.57	0.91
Not at all	134	29.0%	124	25.3%	258	27.1%		
A little	85	18.4%	76	15.5%	161	16.9%		
Quite a bit	28	6.1%	24	4.9%	52	5.5%		
Very much	13	2.8%	18	3.7%	31	3.3%		
**Pain in bones/muscles/joints**								
Did not suffer	191	41.3%	210	42.7%	401	42.0%	0.65	0.64
Not at all	99	21.4%	107	21.7%	206	21.6%		
A little	105	22.7%	108	22.0%	213	22.3%		
Quite a bit	44	9.5%	40	8.1%	84	8.8%		
Very much	24	5.2%	27	5.5%	51	5.3%		
**Painful/gritty eyes**								
Did not suffer	192	41.5%	232	47.4%	424	44.5%	0.18	0.91
Not at all	110	23.8%	124	25.4%	234	24.6%		
A little	118	25.5%	89	18.2%	207	21.7%		
Quite a bit	28	6.0%	22	4.5%	50	5.3%		
Very much	15	3.2%	22	4.5%	37	3.9%		
**Weight gain**								
Did not suffer	52	11.3%	67	13.8%	119	12.6%	0.017	0.034
Not at all	113	24.6%	125	25.7%	238	25.1%		
A little	157	34.1%	179	36.8%	336	35.5%		
Quite a bit	82	17.8%	82	16.8%	164	17.3%		
Very much	56	12.2%	34	7.0%	90	9.5%		

$ - Trend test with "Did not suffer" and "Not at all" combined into one group
& - Fisher's exact test comparing the proportion reporting "Quite a bit" or "Very much"

Table A19 - GEE models of QOL/GHS data

	Model variable	Coefficient	99% CI		p-value
			Lower	Upper	
QLQ-C30 Global Health Status subscale (N=1098)	E/aE	-0.04	-2.27	2.19	0.96
	CMF/X	3.59	1.35	5.83	<0.0001
	Baseline score	0.46	0.39	0.53	<0.0001
	Time to questionnaire	0.19	0.17	0.21	<0.0001
	Age	0.00	-0.12	0.13	0.94
	Component of QOL study	-0.21	-2.53	2.11	0.82

In addition to randomised treatment, GEE models also included baseline score, time from baseline to follow-up questionnaire completion and age at randomisation.
No statistically significant interaction seen between E/aE and CMF/X treatment

TacT 2

TRIAL OF ACCELERATED CHEMOTHERAPY

Trial of **A**ccelerated **A**djuvant **C**hemo**T**herapy with Capecitabine in Early Breast Cancer (TACT2)

This trial is part of the NCRN portfolio, endorsed by CTAAC and co-sponsored by Lothian Health Board and The Institute of Cancer Research

Protocol Version: 6, 29th April 2015
MREC ref: 04/MRE00/88
CTA number: 17844/0204/001-0031
EudraCT number: 2004-000066-13
ISRCTN68068041

ADMINISTRATION

This protocol is a controlled document and should not be copied, distributed or reproduced without the written permission of the ICR-CTSU.

Chief Investigator:
David Cameron
Edinburgh Cancer Research Centre
Tel: 0131 777 3512
Fax:0131 777 3520
Email: D.cameron@ed.ac.uk

Clinical co-ordinators:
Peter Canney
Retired
Email: pg.canney@me.com
Peter Barrett-Lee
Velindre NHS Trust, Whitchurch
Cardiff CF14 2TL
Tel.: 029 2031 6914 Fax: 029 2031 6267
Email: Peter.Barrett-Lee@velindre-tr.wales.nhs.uk

The ICR-Clinical Trials & Statistics Unit (an NCRI cancer clinical trials unit) has overall responsibility for the conduct of the trial.

Statistician: Judith Bliss
Email: Judith.Bliss@icr.ac.uk
Trial Management: Jane Banerji
Email:Jane.banerji@icr.ac.uk

ICR-CTSU, Section of Clinical Trials
Institute of Cancer Research,
Cotswold Road, Sutton,
Surrey SM2 5NG
Tel: 020 8722 4349 Fax: 020 8722 4368

The trial is also managed on a day to day basis by the following trials offices on behalf of their regional research groups:

Cancer Clinical Trials Unit Scotland (CaCTUS) *(also running the Quality of Life Study)*
Gyle Square, 1 South Gyle Crescent, Edinburgh EH12 9EB
Tel: 0131 275 7061

Northern & Yorkshire Clinical Trials Research Unit, Leeds Institute of Clinical Trials Research, University of Leeds, Leeds, LS2 9JT
Tel: 0113 343 8088

CR-UK Clinical Trials Unit, Institute for Cancer Studies, Vincent Drive, Edgbaston, Birmingham, B15 2TT
Tel: 0121 414 3057/4371

Quality of Life Study Coordinator: Galina Velikova (Leeds)
Quality of Life Study (Protocol Design) : Penny Hopwood (Manchester)
Biological studies: John Bartlett (Toronto, Canada and Edinburgh)
Health Economics Study Coordinator: David Bloomfield (Brighton)

Protocol Working Group: John Bartlett (Edinburgh), David Bloomfield (Brighton), Murray Brunt (Stoke), Robert Coleman (Sheffield), Helena Earl (Cambridge), Paul Ellis (London), Steve Johnston (London), Robert Leonard (Swansea), Chris Poole (Birmingham), Anne Robinson (Southend), Ian Smith (London), Robert Stein (London), Chris Twelves (Bradford), Mark Verrill (Newcastle), Andrew Wardley (Manchester)

TACT2 protocol version 6 approved by:

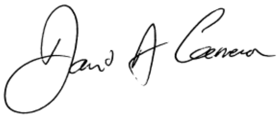

Signed……………………………………………………………… date …29th April 2015

This clinical trial protocol is intended to provide guidance and information only for the conduct of the TACT2 Trial in participating centres. It is not for use as a guide for the management of other patients outside of the trial. Protocol amendments will be circulated to participating centres as they occur.

Contents **Page**

TRIAL SCHEMA 4
INTRODUCTION 5
BACKGROUND & RATIONALE 5
ADJUVANT THERAPY FOR EARLY STAGE BREAST CANCER 5
MORE RECENT ADJUVANT STUDIES 6
ACCELERATED CHEMOTHERAPY 7
CAPECITABINE (XELODA®) 8
MALE BREAST CANCER 8
TRIAL DESIGN 8
PRIMARY ENDPOINT: 9
SECONDARY ENDPOINTS: 9
TRIAL PROCEDURES 9
STAGING INVESTIGATIONS 9
ELIGIBILITY 9
INCLUSION CRITERIA 9
EXCLUSION CRITERIA 10
RANDOMISATION PROCEDURE 11
STUDY ORGANISATION 11
TREATMENT PLAN - NON-INVESTIGATIONAL THERAPY 12
SURGERY 12
ENDOCRINE THERAPY 12
RADIOTHERAPY 12
TREATMENT PLAN - INVESTIGATIONAL THERAPY 13
CHEMOTHERAPY 13
PEGYLATED GCSF 13
PRE-CHEMOTHERAPY INVESTIGATIONS 13
SUPPORTIVE MEDICATION 14
DOSE MODIFICATIONS IN RESPONSE TO TOXICITY 14
CAPECITABINE 15
DRUG SUPPLIES & LABELLING 17
PHARMACOVIGILANCE 18
DEFINITIONS 18
RECORDING & REPORTING OF SAEs 19
FOLLOW-UP OF SARs, SAEs & SUSARs 20
OTHER TOXICITY REPORTING 20
PATHOLOGY REPORTING 21
TRIAL EVALUATIONS 21
SCHEMA OF TRIAL EVALUATIONS 22
STATISTICAL CONSIDERATIONS 23
STRATIFICATION 23
RANDOMISATION 24
SAMPLE SIZE 24
ANALYSIS PLAN 24
INTERIM ANALYSES & ROLE OF THE DATA MONITORING & ETHICS COMMITTEE (DMEC) 25
MILESTONES 26
COMPLETION OF THE STUDY & DEFINITION OF STUDY END DATE 26
PREDICTIVE MARKER STUDIES 26
COMPATIBILITY WITH OTHER STUDIES 26
RESEARCH GOVERNANCE 27
TRIAL ADMINISTRATION & LOGISTICS 27
PROTOCOL COMPLIANCE & MONITORING 28
DATA ACQUISTION & ON-SITE MONITORING/AUDITING 29

ARCHIVING 29
FINANCIAL MATTERS 30
CLINICAL RISK ASSESSMENT 30
PUBLICATION POLICY 30
CONFIDENTIALITY & LIABILITY 30
LIABILITY / INDEMNITY / INSURANCE 30
PATIENT CONFIDENTIALITY 30
ETHICAL CONSIDERATIONS 31
WITHDRAWAL OF PATIENTS FROM STUDY TREATMENT 31
REFERENCES 32
APPENDIX 1 Sub-study 1: Quality of Life & Toxicity 34
APPENDIX 2 Sub-study 2: Biology 41
APPENDIX 3 Sub-study 3: Cost Consequences of Trial Participation 42
APPENDIX 4 Sub-study 4: Evaluation of Cardiac Function 45
APPENDIX 5 Radiotherapeutic Procedures 47
APPENDIX 6 ECOG Performance Status 51
APPENDIX 7 New York Heart Association Functional Classification 52
APPENDIX 8 Common Terminology Criteria for Adverse Events 53
APPENDIX 9 Sample Patient Information Sheet, consent forms & GP letter 54
APPENDIX 10 Compatibility with Other Studies 55

TRIAL SCHEMA

Early invasive breast cancer completely resected

Adjuvant chemotherapy indicated

↓

Stratification

- Centre
- Nodal status: (Node negative, node positive)
- Age: ≤ 50, > 50yrs
- ER: negative and not for endocrine therapy, positive

↓

Randomisation

↓

① 4 cycles of epirubicin every 3 weeks followed by 4 cycles of Classical/Bonadonna CMF	② 4 cycles of epirubicin every 2 weeks with pegylated GCSF support followed by 4 cycles of Classical/Bonadonna CMF
③ 4 cycles of epirubicin every 3 weeks followed by 4 cycles of oral capecitabine	④ 4 cycles of epirubicin every 2 weeks with pegylated GCSF support followed by 4 cycles of oral capecitabine

INTRODUCTION

Breast cancer is the most common malignancy to afflict women in the Western World. There are over 41,000 new cases each year in the UK alone, with an annual mortality of approximately 13,000 [1]. One woman in nine is affected at some time in her life. Whilst the age incidence curve for breast cancer is similar to most other solid tumours, it remains the major killer of women in childbearing years. Besides the morbidity and mortality caused to the woman herself, breast cancer also has profound psychological and economic consequences for the family. More effective treatments are urgently required. Our knowledge of the natural history of this disease suggests that any significant improvement in outcome will depend upon the development of more effective adjuvant therapy for women presenting with early stage disease [2]. This may present a major challenge to NHS resources [3-5], and behoves more precise identification of individual risk as well as elucidation of those factors predicative of treatment benefit.

BACKGROUND & RATIONALE

ADJUVANT THERAPY FOR EARLY STAGE BREAST CANCER

Although early stage breast cancer is by definition grossly limited to the breast and ipsilateral axillary nodes and amenable to surgical resection, occult local and systemic micrometastatic deposits may later develop into a clinically detectable recurrence, and eventually prove fatal. Twenty-year follow-up studies of women presenting with early stage breast cancer in the UK and North America in the 1940's established the limitations of purely loco regional treatment in modifying the natural history of early stage disease [6,7]. Together with preclinical work, which defined susceptibility of micrometastases to chemotherapy [8], these studies provided the justification for the clinical development of systemic adjuvant chemotherapy in the 1970's [9].

Thirty years on, there is now an incontrovertible body of evidence to demonstrate the success of this endeavour. In an overview of 56 trials of prolonged polychemotherapy versus no chemotherapy involving 28,000 women, the Early Breast Cancer Clinical Trialists' Collaborative Group analyses have confirmed that adjuvant chemotherapy reduces the annual odds of recurrence by 23.4% (SE 1.9), and the odds of death by 15% [10]. This reduction in recurrence emerges chiefly during the first 5 years of follow-up, whereas the survival advantage grows throughout the first 10 years. Subgroup analyses of these data have provided further information about relative benefit from treatment, by axillary lymph node involvement, age, menopausal status, oestrogen receptor (ER) status.

The 1998 Oxford Overview sub-group analyses

Nodal Status

The proportional reduction in risk of recurrence afforded by chemotherapy is similar for women with node-negative and node-positive disease. The 10-year survival of those with node-negative disease is increased from 71% to 78% (an absolute benefit of 7%); the 10-year survival of those with node-positive disease increases from 42% to 53% (an absolute benefit of 11%).

Age

In women aged 50-69 years, the proportional reduction in mortality is smaller than that observed in younger women, 10-year survival of node-negative women increasing from 67% to 69% with polychemotherapy and for node positive disease, the 10-year survival increases from 46% to 49%.

Tumour ER status

Amongst women aged under 50, the overview shows substantially reduced risk of recurrence with combination chemotherapy, both for those with ER-poor disease (40% [SD 7]) and those with ER-positive tumours (33% [SD 8]). These figures were not significantly different from one another. By contrast, among women aged 50-69, the proportional reduction in recurrence appeared to be nearly twice as large in women with ER-poor disease (30% [SD 5]) as in those with ER-positive disease (18% [SD 4]), and the difference between these effects was conventionally significant (heterogeneity between proportional reductions χ21=4·5; 2p=0·03).

The effects of polychemotherapy on recurrence also appeared to be somewhat smaller for women with ER-positive disease when the two age groups were combined (heterogeneity, stratified for age, χ21=4·9; 2p=0·03). However, in both age ranges, the reduction in recurrence among women with ER-positive disease was highly significant (both 2p<0·00001), indicating that in neither age range can such hormone-receptor measurements discriminate a group of women who would fail to benefit from treatment.

Treatment comparisons: anthracyclines

The EBCTCG identified 11 randomised trials, involving a total of nearly 7000 patients, which compare anthracycline-containing regimens, such as FAC or FEC, versus CMF alone. Taken together, the addition of anthracyclines yielded a further 12% (SD 4) proportional reduction (2p=0·006) in the odds of recurrence, with no significant heterogeneity between the effects seen in the different trials. There was also a marginally significant 11% (SD 5) proportional reduction in mortality with the anthracycline-containing regimens (2p=0·02) at 5 years.

MORE RECENT ADJUVANT STUDIES

Three more recent randomised trials have been reported none of which were available for incorporation in the 1998 Oxford Overview.

The first of these studies (CAN-NCIC-MA-5) randomised 716 pre-and peri-menopausal women lymph node positive tumours to either six cycles of FEC-120 (epirubicin 60mg/m^2, d 1 and 8; oral cyclophosphamide 75 mg/m^2 /d, d 1-14; 5-fluorouracil 500 mg/m^2 i.v. d1 and 8; q 28 days) or classical CMF [11]. The median age of the study population was 45 years. Approximately 60% of patients had 1 - 3 involved nodes, and approximately 40% had ≥ 4 nodes involved with tumour. Patients in the epirubicin-treated group had a significantly increased 5-year relapse-free survival rate (62% versus 53%) and an increased 5-year overall survival (77% versus 70%) compared with those treated with classical CMF.

A second study (FRE-GFEA-05) compared a higher dose epirubicin-containing regimen (FEC-100; epirubicin 100 mg/m^2 i.v., cyclophosphamide 500 mg/m^2 i.v., 5-Fluorouracil 500 i.v. mg/m^2, q 21 d) with a lower dose epirubicin-containing regimen (FEC-50; epirubicin 50 mg/m^2 i.v.; cyclophosphamide 500 mg/m^2 i.v.; 5-Fluorouracil 500 i.v. mg/m^2; q 21 d) in 565 pre- and postmenopausal women with either ≥4 nodes involved, or 1 to 3 node-positive if tumours were ER-negative/PgR-negative, with a histological grade of 2 or 3 [12]. The median age was 51 years and approximately half of the patients were postmenopausal. Patients treated with the higher-dose epirubicin regimen had a significantly greater 5-year relapse-free survival rate (65% versus 52%, log-rank p=0.007) and 5-year overall survival (76% versus 65%, log-rank p=0.007) than patients given the lower-dose epirubicin regimen. The overall reduction in risk of relapse was 32%. The relative reduction in the risk of death was 31% [12].

Although neither trial was powered for subset analyses, improvements in RFS and OS were observed both in patients with 1-3 nodes positive and in those with ≥ 4 nodes involved when comparing the FEC-120 or FEC-100 groups with their respective controls. Furthermore, in the dose intensity study, similar improvements in RFS and OS were observed in both pre- and postmenopausal women treated with FEC-100 compared with FEC-50.

The third is the largest ever adjuvant anthracycline study, the prospectively planned meta-analysis of the two UK NEAT trials, which compared the sequential epirubicin-CMF (E-CMF) regimens with CMF. Both these trials show a statistically significant benefit for E-CMF over CMF, and have a combined HR of 0.7 (0.58 – 0.85 95% c.i.) for disease-free survival and 0.64 (0.51 – 0.81 95% c.i.) for overall survival (ASCO 2003 abstract 13).

As a result of these data from the NEAT trials, many UK breast oncologists feel that the sequential E-CMF regimen should be the standard anthracycline regimen, against which alternatives should be compared.   It is clear however that there remains significant toxicity associated with the CMF part, with for example, all treatment related deaths in the NEAT studies occurring during administration with CMF [13].

ACCELERATED CHEMOTHERAPY

After the failure of pure dose escalation to improve upon the efficacy of adjuvant chemotherapy, attention has switched to increased frequency of administration, or so called dose dense chemotherapy. The limiting toxicity is usually myelosuppression, but this can be circumvented by the use of Granulocyte Colony Stimulating Factors (GCSF). This approach has proved effective in both small cell lung cancer and lymphoma, but until recently there were no substantive data in breast cancer [14,15,16]. This has changed with the preliminary reports of the CALGB9741 trial, which has shown an improvement in disease-free survival if AC followed by paclitaxel is given at a 2-weekly rather than the conventional 3-weekly interval with GCSF support [17]. The data published in 2003 were based on a pre-planned 3-yearly analysis rather than a pre-defined number of events, and as such there have been insufficient events to detect the difference originally hypothecated. However, coupled with the reported benefits seen in small cell lung cancer and lymphoma, these data suggest further testing is needed. It should also be noted, that in keeping with the studies in small

cell lung cancer and lymphoma, accelerated therapy appears to be associated with less neutropenia than 3-weekly therapy. This was confirmed in an Italian randomised trial of 1214 women presented at the 2003 San Antonio Breast Cancer Symposium, in which there were NO cases of febrile neutropenia in the women randomised to two-weekly FE60C supported by GCSF (Venturini et al SABCS 2003 abstract 12). This same study reported a survival advantage for this approach in women under the age of 50.

The availability of pegylated GCSF has the potential to further reduce the incidence of febrile neutropenia, as it has been recently reported that as compared to conventional daily GCSF (the preparation used in the above studies of accelerated chemotherapy), it may reduce the incidence of neutropenic sepsis by up to 35%.

CAPECITABINE (XELODA®)

This oral 5-FU prodrug has recently gained UK licence and NICE approval for use in advanced breast cancer. No major phase III studies comparing it to other established regimens have been conducted, but the data that are available report little difference between the response rate to this drug and either i.v. CMF or i.v. paclitaxel [18,19]. Similarly there are few robust reports on its tolerability, but it has rapidly become one of the standard regimens to be used in advanced disease after failure of anthracyclines and taxanes.

Of greater interest is the biology of this agent. The final step in its conversion to the active moiety, 5-FU, requires the enzyme thymidine phosphorylase. This is often preferentially expressed in tumours, increasing the potential therapeutic index. A number of anti-cancer therapies appear to up regulate this agent, and recent data suggest that epirubicin is one such cytotoxic agent [20].

It is therefore an attractive drug to use: it requires no intravenous administration, rarely causes neutropenia or other toxicities requiring hospital admission. Given after epirubicin there is the potential added advantage that any remaining micro-metastatic disease is “primed” for increased sensitivity to this agent.

MALE BREAST CANCER

This study will be the first study of adjuvant chemotherapy of which we are aware for which male patients are eligible. Breast cancer is much rarer in men, occurring with about 0.5 – 1% of the frequency of women. However the available literature suggests that matched stage for stage their outcome is similar, and they are managed in much the same manner. There are recent UK studies that have permitted male patients including the currently recruiting “Will Weekly Win” taxol trial, and therefore TACT2 represents a unique opportunity to generate some data from a randomised trial in this hitherto poorly studied subgroup of breast cancer patients.

TRIAL DESIGN

A randomised, phase III clinical trial with a 2 x 2 factorial design addressing two hypotheses:
(1) that accelerating epirubicin will improve the efficacy of the sequential schedules (based originally on the NEAT epirubicin/CMF schedule).

(2) that the substitution of CMF by capecitabine will not be detrimental to patient outcome but will offer advantages in Quality of Life and/or toxicity.

PRIMARY ENDPOINT:

- Time to Recurrence

SECONDARY ENDPOINTS:

- Overall survival (OS)
- Distant disease-free survival (DDFS)
- Disease Free Survival as defined by the STEEP criteria (21)
- Tolerability (including Serious adverse events (SAE)), dose-intensity and toxicity
- Detailed Toxicity and Quality of Life in the subset of patients studied

For the survival-based endpoints (DFS, OS, DDFS) the nominal time-point of interest will be 5 years after randomisation.

TRIAL PROCEDURES

STAGING INVESTIGATIONS

Required staging investigations will be minimal and in keeping with standard UK practice in breast cancer management. All patients should have a FBC, biochemical screen, to include liver function tests, creatinine and serum calcium. A CXR is required for all patients with 4 or more positive axillary nodes, and recommended (but not mandated) for all other patients. Further staging investigations will be performed if considered to be clinically indicated, and an isotope bone scan and/or liver ultrasound are expected to be performed routinely in higher risk patients (such as those with >3-node positive), those with suspicious symptoms and/or abnormal biochemistry.

ELIGIBILITY

INCLUSION CRITERIA

- Histological diagnosis of invasive breast carcinoma
- Completely resected disease with negative surgical margins (apart from deep margin if full thickness resection or involved radial margins following wide local excision if further surgery to the breast is planned on completion of chemotherapy).
- Early stage disease (T0-3 N0-2 M0) without clinical suspicion/evidence of distant metastases on routine staging
- Definite indication for adjuvant chemotherapy
- ECOG status 0 or 1
- Aged over 18 years (no upper age limit)
- Fit to receive any of the trial chemotherapy regimens, with adequate bone marrow, hepatic, and renal function i.e.
    - Hb > 9g/dL; WBC > 3 × 10^9/L; platelets > 100 x 10^9/L
    - Bilirubin within normal range (unless known Gilbert's disease)
    - AST/ALT ≤ 1.5 x Upper limit of normal (ULN)

    - Albumen within normal range
    - Creatinine ≤ 1.5 x ULN and calculated creatinine clearance using Cockroft-Gault formula > 50 ml/min
    - No active, uncontrolled infection
- Signed TACT2 trial consent form
- Randomisation within 8 weeks of surgery, but ideally within 1 month
- No previous chemotherapy, hormonal therapy or radiotherapy for the treatment of pre-invasive or invasive cancer except:
    - Previous radiotherapy for basal cell carcinoma
    - Previous pre-operative endocrine therapy provided that there was no evidence of progression during this therapy, that it was for less than 6 weeks in duration, and was stopped at least one month prior to trial entry
- No previous malignancy except in the case of DCIS, or basal cell carcinoma or cervical carcinoma in situ, or where the patient has been disease-free for 10 years, and where treatment consisted solely of resection
- Non-pregnant and non-lactating, with no intention of pregnancy during chemotherapy, and prepared to adopt adequate contraceptive measures if pre-menopausal and sexually active
- No concomitant medical, psychiatric or geographic problems that might prevent completion of treatment or follow-up

EXCLUSION CRITERIA

- Only cytological proof of malignancy
- No evidence of invasive breast cancer
- Previous invasive breast cancer or bilateral breast cancer (DCIS or LCIS that has been surgically treated or is planned for surgical treatment post-chemotherapy is allowed provided there is no evidence of further disease in the contralateral breast)
- Locally advanced breast cancer (T4 and/or N3 disease)
- Patients who have had breast conserving surgery in whom there is a contra-indication for, or refusal of post-operative radiotherapy
- Patients with positive surgical margins unless either
    - Deep surgical margin involvement following full thickness resection
    - Non-invasive cancer at surgical margins and a decision to perform mastectomy on completion of chemotherapy has already been made
- Patients not able or willing to give informed consent
- Patients known not to be available for a minimum of 5 years' follow-up
- Patients with known serious viral infection such as active Hepatitis B, Hepatitis C or HIV
- Patients with significant cardiac disease, such as impaired left ventricular function or active angina (requiring regular anti-anginal medication and/or resulting in restricted physical activity)
- Patients with a history of significant renal impairment or disease
- Simultaneous participation in the active intervention phase of another treatment trial
- Being approached and recruited into the active intervention phase of another treatment trial two months before or after recruitment into TACT2

RANDOMISATION PROCEDURE

ALL PATIENTS MUST BE RANDOMISED BEFORE CHEMOTHERAPY BEGINS

Sufficient time (e.g. 7 days) should be allowed for the patient to decide on trial entry, but the time which elapses between randomisation and start of chemotherapy should be minimised. It is recommended that patients considered for TACT2 are booked for their first chemotherapy treatment at the time of first referral.

An eligibility checklist and randomisation checklist should be completed prior to randomisation. To randomise a patient, simply telephone or fax your affiliated office:

Trials office	Telephone	Fax
TACT2 Trials Office at ICR-CTSU	020 8643 7150	020 8722 4368
CaCTUS, Edinburgh	0131 275 7276 or 0131 316 4278	0131 275 7512
Clinical Trials & Research Unit, Leeds	0113 343 6260	0113 343 1471
CR-UK Clinical Trials Unit, Birmingham	0121 414 3366 or 0121 414 7844	0800 328 6412

The person randomising the patient will then be asked to confirm that an eligibility checklist has been completed and to verify that the patient has signed the TACT2 consent form (this will be the subject of a later audit). They will also be asked for all the information on the randomisation checklist. A trial number and treatment allocation will be given over the phone and later confirmed in writing.

STUDY ORGANISATION

It is intended that TACT2 will randomise approximately 4400 patients, primarily from an estimated 100 centres in the UK. The aim is to complete accrual within 3 years if possible by maximising the number of UK centres and the speed with which they are activated.

Patient enrolment began in December 2005, the completion of enrolment is planned for late 2008 / early 2009 and completion of disease-free and survival status at 5 years is expected in early/mid 2013.

Several trials offices will undertake randomisation and data management. Each centre should agree its affiliation to one of the trials offices prior to participating in the study and enter all patients via the same trials office for the life of the trial. Data generated will be collected by the respective trials offices, who are responsible for checking incoming CRFs for compliance with the protocol, inconsistent and missing data, and for computerising data and resolving data queries. Data from all participating trials offices will be pooled and analysed at ICR-CTSU who have overall responsibility for all trial data, and for the ICR-CTSU SOPs that describe how the trial is to be conducted within participating trials offices. All data will be handled, computerised and stored in accordance with the Data Protection Act 1998. Quality control will be maintained through adherence to ICR-CTSU SOPs and through regular meetings of data management and statistical representatives from the participating trials offices.

TREATMENT PLAN - NON-INVESTIGATIONAL THERAPY

SURGERY

Patients may undergo breast conservation surgery with axillary clearance (preferably level III), or modified radical mastectomy, as per UK BASO guidelines. Patients undergoing sentinel node biopsy or a level 1 axillary sample MUST have either subsequent full axillary clearance or axillary radiotherapy if there is any evidence of nodal involvement by routine H&E staining. The date of surgery is the final date of surgery for malignancy, including re-excision, axillary clearance or mastectomy following initial breast conservation. If a sentinel lymph node biopsy or other sampling procedure is performed at initial surgery, and further axillary surgery is indicated due to detection of lymph node involvement, this may be carried out before randomisation (and date or surgery will be date of later axillary surgery) or alternatively after completion of chemotherapy. In this case any outstanding pathology data should be provided as soon as it is available. Reconstructive surgery conducted at the same time as surgery for malignancy is acceptable: in the event of a separate re-constructive or other surgical intervention (for example for flap necrosis), the date of surgery is the date of the previous intervention for malignant disease. Ideally, patients should be randomised into TACT2 within 4 weeks of surgery, but will be accepted into the trial up to 8 weeks from date of surgery for malignant disease.

ENDOCRINE THERAPY

Any previous HRT or pre-operative endocrine therapy is to be stopped at least 4 weeks prior to chemotherapy. All patients with ER and/or PgR positive tumours should commence treatment with tamoxifen 20mg daily for 5 years, within 4 weeks cessation of chemotherapy. In patients for whom tamoxifen is not appropriate (e.g. history of deep venous thrombosis, contravenes agreed local protocol), an aromatase inhibitor may be offered as an alternative. Aromatase inhibitors should only be used in accordance with local, regional or network policy, or for premenopausal women within the context of the SOFT study (vide infra).

LHRH analogue for ovarian protection in pre-menopausal women may be offered concurrently with chemotherapy, permitting concomitant recruitment into the NCRN OPTION trial. Pre-menopausal women who are still menstruating may be enrolled in the SOFT study, which randomises such patients between tamoxifen, ovarian ablation plus tamoxifen, and ovarian ablation plus exemestane, each for 5 years.

RADIOTHERAPY

Radiotherapy should be given, if required, after chemotherapy in keeping with local practice, and/or guidelines in Appendix 4.

TREATMENT PLAN - INVESTIGATIONAL THERAPY

CHEMOTHERAPY

Patients will be randomised into one of the four treatment arms (E-CMF; accelerated E-CMF; E-X; accelerated E-X) in a 1:1:1:1 ratio:

	NON-ACCELERATED	ACCELERATED
TREATMENT A	① 4 cycles of epirubicin every 3 weeks followed by 4 cycles of Classical/Bonadonna CMF	② 4 cycles of epirubicin every 2 weeks with pegylated GCSF support followed by 4 cycles of Classical/Bonadonna CMF
TREATMENT B	③ 4 cycles of epirubicin every 3 weeks followed by 4 cycles of oral capecitabine	④ 4 cycles of epirubicin every 2 weeks with pegylated GCSF support followed by 4 cycles of oral capecitabine

Doses:

**Epirubicin**	100 mg/m^2
**Classical / Bonadonna CMF** (on a 28 day cycle)	
Cyclophosphamide	Either 100 mg/m^2 p.o. per day for 14 days or day 1 & 8 600 mg/m^2 i.v.
Methotrexate	40 mg/m^2 days 1 & 8
5-FU	600 mg/m^2 days 1 & 8
**Capecitabine**	2,500 mg/m^2 in divided doses for 14 days every 3 weeks

PEGYLATED GCSF

In the experimental accelerated arm, all patients should receive a single dose of 6mg pegylated-GCSF, (pegfilgrastim, Neulasta), on the day after the epirubicin as per the product license. In the event of safety and efficacy data becoming available that the drug is equally effective if given on the day of chemotherapy, then the protocol will be amended accordingly.

In treatment arms 1 and 3 the use of GCSF (preferably pegylated GCSF: see appendix 3) as secondary prophylaxis is encouraged following an episode of neutropenic sepsis or significant (>8 days) dose delay during cycle 1.

PRE-CHEMOTHERAPY INVESTIGATIONS

The following investigations are expected to be undertaken routinely before each cycle of chemotherapy in both treatment arms: symptom review, toxicity review, FBC, biochemical

profile (including liver function tests and serum creatinine and during capecitabine administration, calculated creatinine clearance).

Day 1 chemotherapy, and day 8 in the case of CMF, should only be administered if the neutrophil count >1.0 x 10^9/L and platelets >100 x 10^9/L.

SUPPORTIVE MEDICATION

Antiemetics

These may be given according to local practice. However, we recommend a 5HT3 antagonist (e.g. granisetron 3 mg i.v., or ondansetron 8 mg i.v.) and dexamethasone 8 mg i.v., before epirubicin, followed by domperidone 10-20 mg p.o. tds × 5 days, with dexamethasone 2 mg po tds x 3 days only.

H2-antagonists etc.

Ranitidine 150mg p.o. b.d. × 7 days, or similar, may be necessary to relieve steroid induced dyspepsia. Patients on regular cimetidine should be switched to another H2 receptor blocker or PPI as clinically appropriate prior to starting epirubicin in order to avoid the risk of increased epirubicin toxicity when used in conjunction with cimetidine.

Folinic acid rescue.

This is not routine in the use of CMF, but if it is a centre policy it is permitted provided it is given to all patients in CMF arms, and is not started until at least 24 hours after the CMF is administered.

Aperients

Aperients and/or glycerine suppositories will be occasionally required for relief or prophylaxis of granisetron-related constipation.

Prophylactic mouthwashes

Corsadyl mouthwash p.o. b.d is allowed throughout the period of anthracycline-containing chemotherapy.

Antibiotics

Prophylactic antibiotic therapy is not recommended.

DOSE MODIFICATIONS IN RESPONSE TO TOXICITY

Every effort must be made to deliver chemotherapy on schedule except where clinically a delay is indicated. In such circumstances, delays should be kept to a minimum, and clinicians should avoid the practice of automatically deferring by one week for minor haematological reasons: patients should be re-treated as soon as is clinically appropriate. The secondary prophylactic use of GCSF in the control arms (arms 1 and 3) after an episode of significant neutropenic delay is encouraged but not mandated.

Nadir full blood tests

Routine nadir blood counts are not required: dose modifications on the basis of nadir counts are specified for patients who have blood counts measured between treatments when experiencing significant toxicity.

Recommended Dose Modifications

Recommended dose modifications are specified separately for each component of the chemotherapy regimens.

Epirubicin

Patients experiencing nadir platelet counts ≤20 x 10^9/L, absolute neutrophil counts (ANC) < 0.25 x 10^9/L, neutropenic fever, or grades 3/4 non-haematological toxicity (e.g. mucositis) should have the subsequent day 1 dose of epirubicin reduced to 80% of previous dose. Day 1 chemotherapy should be delayed until platelet counts are ≥ 100 x 10^9/L, ANC ≥ 1.0 x 10^9/L, and non-haematological toxicities have recovered to ≤ CTC grade 1. If delay of a week or more is required, doses should be reduced to 80% of original.

Cardiac toxicity

This is not anticipated at the cumulative doses of epirubicin achieved in this protocol, namely 400mg/m^2 however, occasional patients with pre-existing cardiac pathology may develop problems, and clinicians should be alert to this possibility. In the event of congestive cardiac failure developing, patients should be investigated and treated as appropriate. If confirmed, epirubicin should be discontinued, and other chemotherapy may be given at the discretion of the investigator.

CMF

Patients experiencing nadir platelet counts ≤20 x 10^9/L, absolute neutrophil counts (ANC) < 0.25 x 10^9/L, neutropenic fever, or grades 3/4 non-haematological toxicity (e.g. mucositis) should have the subsequent day 1 doses of cyclophosphamide, methotrexate and 5-FU reduced to 80% of previous. Day 1 chemotherapy should be delayed until platelet counts are ≥ 100 x 10^9/L, ANC ≥ 1.0 x 10^9/L, and non-haematological toxicities (except alopecia) have recovered to ≤ CTCAE grade 1. If a delay of a week or more is required, doses should be reduced to 80% of original. In the event that on day 8 the platelet counts are < 100 x 10^9/L or ANC < 1.0 x 10^9/L, then that dose should be omitted, and the patient re-treated with day 1 of the next cycle being on the same date as originally planned.

CAPECITABINE

Patients should be carefully monitored for toxicity, particularly during the first cycle. All patients receiving capecitabine should be reviewed on day 8 of the first cycle in order to identify the very small proportion of patients with extreme sensitivity to 5-FU as a consequence of DPD deficiency. Such patients will also have problems with CMF and would be identified by the day 8 visit of their first CMF cycle. ANY patient experiencing ≥ grade 2 diarrhoea, mucositis or hand-foot syndrome (PPE, palms of the hands or soles of the feet tingle, become numb, painful, swollen, or red) by day 8 of their first cycle should stop their

capecitabine and discontinue study treatment. Toxicity due to capecitabine administration may be managed by symptomatic treatment, dose interruptions and adjustment of capecitabine dose. Once the dose has been reduced it should not be increased at a later time.

A maximum of two dose reductions are allowed per patient. capecitabine treatment interruptions are regarded as lost treatment days and the planned treatment schedule should be maintained. Patients who do not experience a recovery of the toxicity to grade < 1 after a maximum of 21 days delay (measured from the last planned treatment day) must discontinue study treatment.

The following rules will apply:

Grade 1:	Maintain dose level	
Grade 2/ 3:	1st episode	Interrupt until resolved to grade 0-1, then continue at 80% full dose
Grade 2/3:	2nd episode	Interrupt until resolved to grade 0-1, then continue at 64% full dose
Grade 2/3	3rd episode	Discontinue treatment permanently
Grade 4:	1st episode	Discontinue treatment permanently

Administration of capecitabine should be interrupted if treatment-related elevations in bilirubin of >3.0 x ULN or treatment-related elevations in hepatic aminotransferases (ALT, AST) of > 2.5 x ULN occur. Treatment should be resumed when bilirubin decreases to < 3.0 x ULN or hepatic aminotransferases decrease to < 2.5 ULN.

Neutropenia or thrombocytopenia

In case of neutropenia (neutrophils <1 $x10^9$/L) or thrombocytopenia (platelets < $100x10^9$/L) on the planned treatment day, the treatment should be delayed until recovery of neutrophils (neutrophils >1 x 10 9/L) and/or platelets (platelets >100 $x10^9$/L) for a maximum of 21 days.

- In case of a first episode of hematological non-recovery (neutrophils <1 $x10^9$/L or platelets <100 $x10^9$/L) on the planned start day of a new cycle, the treatment should be delayed until recovery of neutrophils (neutrophils ≥1 $x10^9$/L) and/or platelets (platelets ≥100 $x10^9$/L) up to a maximum of 21 days. Subsequent doses should be given at 80%.

- In case of a second episode of hematological non-recovery on the planned start day of a new cycle, the treatment should be delayed until recovery of neutrophils and/or platelets up to a maximum of 21 days. All subsequent doses should be reduced again by 20% to 64% of the initial dose.

- Patients whose counts do not recover after a maximum treatment delay of 21 days (from the planned day of drug delivery) and patients who present a third episode of haematological non-recovery must discontinue study treatment .

It is not anticipated that capecitabine at the dose of 2,500 mg/m^2 in divided doses for 14 days every 3 weeks will cause significant myelosuppression. However in the case of febrile neutropenia or life threatening infection, any further drug delivery should be discontinued for the remainder of that cycle and until full recovery. If on the planned start day of a new cycle, a patient has febrile neutropenia, the next cycle should be delayed until complete resolution. A maximum of 15 days delay is allowed. Doses in all subsequent cycles should be reduced by 20% after a first episode, and by a further 20% (36% of the starting dose) after a second episode. Patients who have febrile neutropenia after a 36% dose reduction should discontinue.

Renal function

It is well recognised that in patients with impaired renal function capecitabine can cause increased toxicity. Therefore, not withstanding the above rules for dosing in the face of toxicity, any patient whose calculated creatinine clearance drops below 50 ml/min should have the dose of capecitabine reduced to 75% of the dose of the previous cycle. If the calculated creatinine clearance falls below 40 ml/min, then they cannot be treated with capecitabine, and if there is no recovery within 21 days of a cycle being due, they must come off study treatment.

DRUG SUPPLIES & LABELLING

Capecitabine (Xeloda®) is supplied free of charge by Roche. Neulasta is supplied by Amgen at a discounted rate. Further details on costing are given in Appendix 3. Guidelines for ordering Xeloda and Neulasta, and drug labelling requirements are contained within the Trial Guidance Notes.

PHARMACOVIGILANCE

DEFINITIONS

Serious Adverse Events (SAEs)

Serious adverse events are those that occur during or within 30 days of administering randomised treatment, whether or not it is related to the randomised treatment. ICH GCP defines an SAE as any untoward medical occurrence shown in Box 1:

BOX 1

- Results in death
- Is life-threatening*****
- Requires in-patient hospitalisation****** or prolongation of existing hospitalisation
- Results in persistent or significant disability/incapacity
- Is a congenital anomaly/birth defect (in offspring of patient regardless of time to diagnosis).
- Is an important medical event (an event that jeopardizes the patient or may require intervention to prevent one of the other outcomes listed above.

* *The term 'life-threatening' in the definition of 'serious' refers to an event in which the patient was at risk of death at the time of the event; it does not refer to an event that hypothetically might have caused death if it were more severe.*

****** *Hospitalisation is defined as an inpatient admission, regardless of length of stay, even if the hospitalisation is a precautionary measure, for continued observation. Hospitalisation for a pre-existing condition, including elective procedures, which has not worsened, does not constitute a serious adverse event.*

For the TACT2 trial, other important medical events that may not result in death, are not life threatening, or do not require hospitalisation may be considered a serious adverse events when, based upon appropriate medical judgement, the event may jeopardise the patient and may require medical or surgical intervention to prevent one of the outcomes listed in Box 1.

Serious Adverse Reactions (SARs)

SARs are those SAEs which are considered to be possibly / probably / definitely related to the trial treatment. Most SARs can be classified as "expected".

Suspected Unexpected Serious Adverse Reactions (SUSARs)

SUSARs are SARs which are not classified as "expected". SUSARs require expedited reporting by the trial sponsor (i.e. Chief Investigator with ICR-CTSU) to MHRA, therefore every effort should be made to notify ICR-CTSU within the timeframe shown below.

RECORDING & REPORTING OF SAEs

SARs shown in Box 2 do not require immediate reporting using an SAE Report Form, but should be reported using a SAR form which provides data required for annual line listings to the MHRA. All other SAEs (except those shown in box 3) should be reported within 24 hours of the investigator becoming aware of it, by completing an SAE Report Form and faxing it to:

ICR-CTSU
Section of Clinical Trials, Institute of Cancer Research, Sutton
FAX No: 020 8722 4368

**BOX 2**<br>**SARs occurring within 30 days of receiving trial therapy that should be reported using a SAR form:**		**BOX 3**<br>**SAEs that do not require immediate reporting using a faxed SAE form:**
**Epirubicin, Neulasta, Cyclophosphamide, Methotrexate, 5FU**<br>**Hospitalisation due to:**<br>• Neutropenia<br>• Febrile neutropenia<br>• Diarrhoea<br>• Infections, including those to Hickman line, catheter or wound<br>• Pyrexia<br>• Sore throat<br>• Nausea or vomiting<br>• Cellulitis	**Capecitabine**<br>**Hospitalisation due to:**<br>• Infections, without grade 3 or 4 neutropenia, including those to Hickman line, catheter or wound<br>• Pyrexia<br>• Sore throat<br>• Nausea or vomiting<br>• Cellulitis	**Any trial drug**<br>• Hospitalisation or death due to disease progression<br>• Hospitalisation for study drug administration, palliative care, terminal care or elective surgery
**NB – All hospitalisations, relapses and deaths <u>MUST</u> be reported on the CRFs**		

Flow diagram of which SAEs require immediate reporting, and action taken following the report

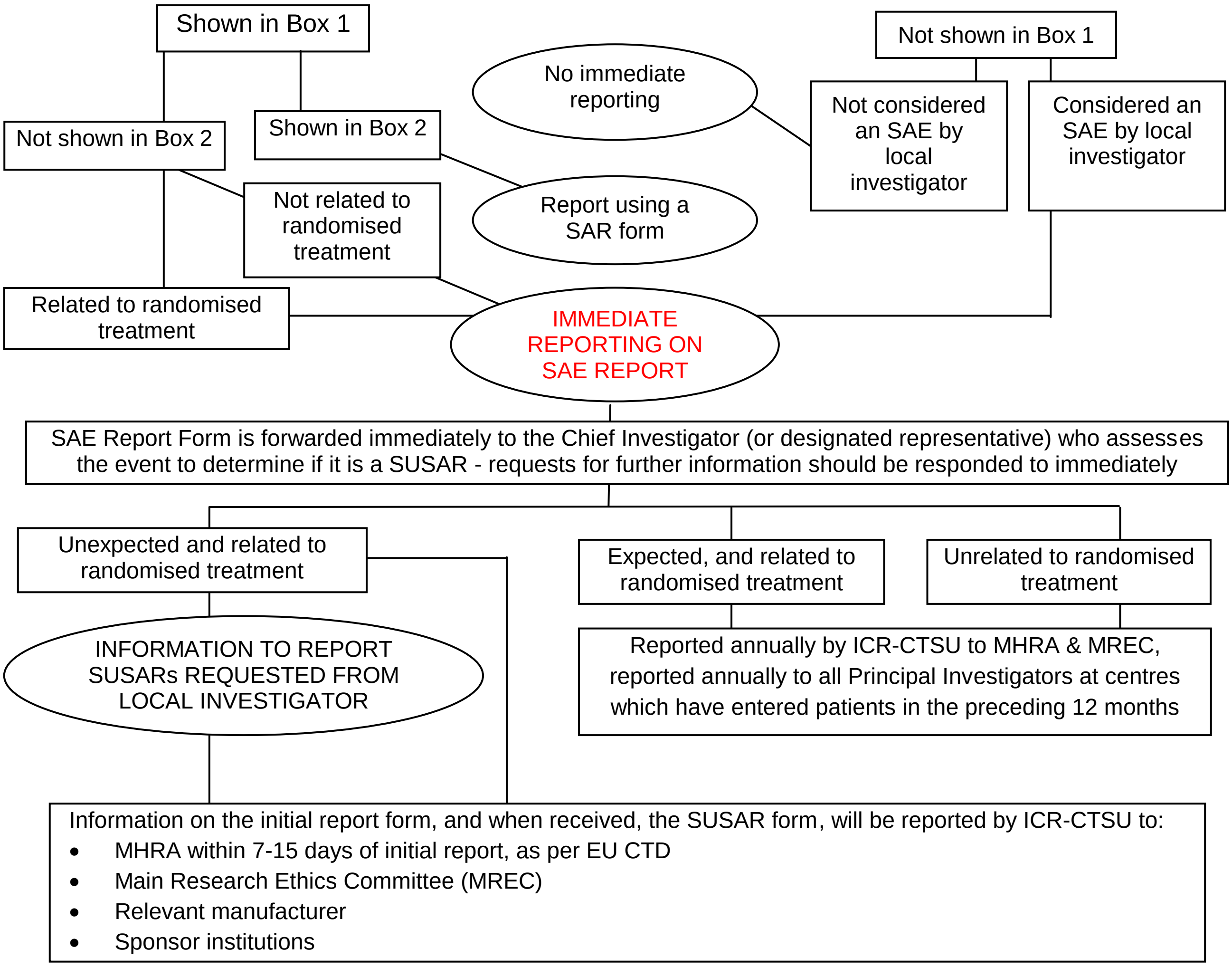

FOLLOW-UP OF SARs, SAEs & SUSARs

The subject must be followed-up until clinical recovery is complete and laboratory results have returned to normal, or until disease has stabilised. Information on final diagnosis and outcome of SARs/SAEs which may not be available at the time the SAR/SAE is initially reported should be forwarded on the SAR/SAE Report Form, as soon as this information is available. Follow-up may continue after completion of protocol treatment if necessary. Any SAE occurring whilst the patient is receiving capecitabine will be passed to Roche. If there is any information that Roche are legally required to collect which has not been captured on the SAE Report Form, the information will be requested. All information regarding an SAE should be faxed to the ICR-CTSU, and information required by drug manufacturers will be passed on by that office. Centres are free to volunteer information to drug manufacturers if they wish, but are under no obligation to do so.

OTHER TOXICITY REPORTING

Any relevant information on SAEs that are shown in Box 1 and also Box 2 will be collected for the whole trial population on the 'NHS Resource Usage form' which is to be completed for all patients at all cycles of chemotherapy.

It is not possible to compare the relative de-merits of chronic low-level toxicities (e.g. grade 1 or 2 hand-foot syndrome) with high-grade acute toxicities. Therefore detailed toxicity reporting on all patients as a discriminator between the chemotherapy treatment arms is of little value in the absence of Quality Of Life measures to assess the global impact upon each patient. Thus detailed clinically assessed toxicity will only be collected via CRFs for those patients entered from centres participating in the Quality Of Life (QL) sub-study (appendix 1). A similar approach to limited recording of detailed toxicity was taken in the CALGB 9741 trial of accelerated chemotherapy.

PATHOLOGY REPORTING

Standard information will be collected on all patients from the local histopathology report. This will include data regarding pathological size, tumour grade, ER status (and actual Allred score/ percentage of +ve cells if available), and the total number of axillary lymph nodes removed, and the number that contain metastatic deposits. In addition, we will record PGR status, and HER2 status where available.

TRIAL EVALUATIONS

Case record forms (CRFs) are listed below. Further details on how and when to complete CRFs and to whom they should be returned are in the Standard Operating Procedures (SOPs). The Trial Management Group reserve the right to amend or add to the CRFs as appropriate. Such changes do not constitute a protocol amendment, and revised or additional forms should be used by centres with immediate effect.

At baseline:

- Eligibility checklist
- Randomisation form
- Baseline form
- Normal Activity form

At the end of each cycle of chemotherapy:

- Chemotherapy Treatment form
- NHS Resource Usage form
- Toxicity form (for centres participating in the QL and Toxicity sub-study)

At the end of radiotherapy (or end of chemotherapy if none is given):

- Adjuvant Treatment form

At 12, 18 and 24 months from randomisation, and annually thereafter:

- Follow-up forms
- Ovarian Function form (pre-menopausal patients only, at 12 months after chemotherapy)

As appropriate:

- Serious Adverse Event (SAE)/Serious Adverse Reaction (SAR) Forms

- Deviation form
- Transfer form
- Recurrence and second primary breast cancer forms
- Death form

SCHEMA OF TRIAL EVALUATIONS

Form	At baseline	chemotherapy cycles 1-8			After RT	12 months	18 months	24 months	annually thereafter
		D1	during	end					
Eligibility checklist	X								
Randomisation checklist	X								
Baseline form	X								
Normal Activity form**	X								
Chemotherapy form		X	*						
NHS resource usage form **				X		X	X	X	X
Toxicity form (for centres participating in the Toxicity sub-study below)				X					
Adjuvant treatment					X				
Follow-up forms						X	X	X	X
Ovarian Function (if required)							X		
Recurrence, second primary & death forms (if required)		X	X	X	X	X	X	X	X
SAE/SAR forms (if required)		X	X	X					
Deviation forms (if required)		X	X	X	X	X	X	X	X
Transfer forms (if required)		X	X	X	X	X	X	X	X

* Patients receiving capecitabine should additionally be seen on day 8 of their first cycle of capecitabine
**these forms to not contain medical information, and source data in patient notes is not required

Patients should be followed-up as per local practice for patients entered into trials in early breast cancer, but the minimum must include 6-monthly clinic visits for toxicity assessments and clinical examination until the end of year 2 and then annual thereafter (to correspond with the follow-up forms). Annual follow-up data will be collected for as long as the Trial Management Group consider it is contributing to the research question. The information required will include sites of recurrence, date of recurrence, vital status, and date and cause of death. Should a patient be discharged to GP care or another hospital, all reasonable attempts should be made by the randomising hospital to collect follow up information from these sources and send it to their affiliated trials office at the prescribed time points laid down in the Trial Guidance Notes. When electronic methods of routine data collection are considered to be reliable and complete, data will continue to be collected via these methods, subject to the approvals required by the "custodian" of that routine data.

Should the GP or other hospital be unable to provide information, the Trials Office should be informed. The Trials Office will then apply to a national records office to either trace the patients' new GP *or* give notification in the event of their death.

Information on second primary breast cancers and other second primary tumours will also be recorded. Regular (annual or bi-annual) imaging of the breasts (e.g. mammography or MRI) should be part of the follow-up protocol for a minimum of 10 years according to local practice.

Long term follow up

Changes of Principal Investigator after the end of the intervention phase of the trial (i.e. when all patients have completed study drug) do not need to be notified to the regulatory authority or the responsible Research Ethics Committee. However, the affiliated Trials Office should be notified of any changes of PI and the local Research and Development office at the Institute of the PI should be informed.

Relapse

Recording of relapses will be done as for TACT. The date of relapse is taken as the date of first confirmed recurrence by an appropriate investigation such as cytology, histology, or imaging wherever possible. In the absence of such confirmation, the date of first clinical suspicion will be taken provided that suspicion leads to a change or re-introduction of anti-cancer therapy. The management of recurrence will be at the discretion of the clinician. Follow up information should continue to be provided until the patient dies. Relapses do not require immediate reporting, and should be recorded on the next due follow up form.

Quality of Life (Appendix 1)

A sub-study addressing quality of life (QL) will be assessed in a cohort of 1000 patients, who will also be assessed for detailed toxicity. The frequency of adverse events and toxicity will be assessed after 800 patients have been entered into the QL study and the planned total of 1000 patients will be adjusted accordingly to allow statistical discrimination between the 4 arms.

Cost and Resource Use *(Appendix 3)*

This assessment will allow for calculations of cost and resource use for adjuvant chemotherapy, and for a comparison between the four different chemotherapy treatments.

STATISTICAL CONSIDERATIONS

STRATIFICATION

Randomisation will be stratified by:

- Centre
- Nodal status: [Node negative, Node positive],
- Indication for endocrine therapy (Yes / No)
- Age: ≤50; >50 years.

Baseline prognostic information on number of nodes involved, grade, ER status, PgR status, HER2 status (where available) and tumour size and radiotherapy usage will be recorded and analyses adjusted for these factors will be conducted as appropriate.

RANDOMISATION

Randomisation will be conducted according to variable sized permuted blocks.

SAMPLE SIZE

The trial will have a 2 x 2 factorial design. The type of hypothesis under investigation is different for the two treatment questions hence the justification of patient numbers and resulting power of the trial is specific to each question. It is assumed that the 5 year DFS in the standard E-CMF arm will be 80%.

i) comparison of standard vs accelerated treatment – the trial aims to detect an improvement in DFS associated with the accelerated schedule

3876 patients will be required to detect a 4% difference (HR=0.78) between the schedules with 90% power and alpha=0.05 (2 sided).

ii) **comparison of E-CMF vs E-X – the trial aims to exclude inferiority of E-X compared with E-CMF**

Evidence from advanced disease suggests that substituting X (Xeloda) for CMF should result in equivalence of E-X and E-CMF, however E-X will be considered a viable alternative if it can be demonstrated that it is not more than 3% worse than E-CMF. A total of 4400 patients will provide 80% chance that the lower 90% confidence limit for the difference between the E-X and E-CMF schedules will exclude 3% if the arms are truly equivalent.

The target accrual is therefore 4400 patients. It is intended that patients will be randomised into both components of the trial, however if centres are unable to accommodate accelerated treatment schedules then they may be permitted to enter only the E-X versus E-CMF comparison.

ANALYSIS PLAN

The analysis of overall survival and disease-free survival will be from the time of randomisation to the date of death or relapse, respectively, or the censor date (date last seen alive/death from other causes). Disease-free survival is taken as the time from trial entry to the date of first confirmed recurrence of this breast cancer. New primary breast cancer within either breast is not considered a relapse, but will still require reporting on follow-up forms. Treatment comparisons will be tested with and without adjustment for the stratification and baseline prognostic factors as above.

Analyses will be based on the intention to treat principle. For the comparison between standard and accelerated schedules the principal analysis will be a logrank comparison of schedules ① and ③ versus schedules ② and ④. For the comparison of E-X with E-CMF a 90% confidence interval for the difference between schedules ① and ② compared with schedules ③ and ④. In both cases Cox regression methods will be used for multivariate

analyses (to further adjust for clinical factors likely to influence prognosis) and to estimate the hazard ratio and its associated confidence intervals and to test for interactions between the schedules. Probabilities of DFS and OS will be presented as Kaplan-Meier survival curves with fixed term survival estimates. Baseline characteristics will be described by randomised treatment group. Comparisons will be performed using simple parametric, non-parametric or chi-squared tests as appropriate. Tests will be two-sided and 95% confidence intervals will be used. Heterogeneity of effects by centre will be investigated.

Toxicity and the frequency and nature of adverse events will be compared between the randomised groups. Summary measures, non-parametric tests as well as analyses incorporating respected measures will be used as necessary. In particular, the proportion of patients experiencing toxicity of CTCAE grade 3 or 4 and the maximum CTCAE toxicity grade will be compared. An investigation of treatment compliance with randomised treatment will be based on frequency of dose reductions and delays. Association will be investigated between observed toxicity and patients' co-morbidity.

INTERIM ANALYSES & ROLE OF THE DATA MONITORING & ETHICS COMMITTEE (DMEC)

A DMEC will be set up to monitor the progress of the trial. The committee members will meet in confidence at regular intervals as they see fit but at least annually. Following each meeting they will produce a report of their findings and recommendations. This report will be submitted to the Trial Management Group and Trial Steering Committee, the main REC and the MHRA, as required.

Interim analysis of side effects, tolerability, disease-free and overall survival for all randomised patients will be performed at approximately yearly intervals. These analyses will be supplied in strict confidence by the trial statistician to the DMEC together with any other analyses that the DMEC may request. In particular, the DMEC will be asked to review emerging data from both randomised comparisons. If evidence emerges from this or other trials that it is no longer ethical to continue randomising patients into one or other randomisation, a change in the design of the trial to reflect this will be considered. No results on survival or recurrence will be made available to investigators or any other party until at least two years after the last patient is entered unless the DMEC determines that it would be unethical to withhold the interim results. Summary data on side effects and compliance will be circulated to investigators every six months. Specific consideration will be given to early toxicity and compliance and also later to any evidence of an interaction between schedules that would negate the appropriateness of the 2 x 2 design and require a consequential increase in patient numbers.

Detailed analysis and publication of the QL and toxicity sub-study may be considered before the primary end-point is reached, but only with the agreement of the Trial Management and Steering Groups.

The main criterion for early stopping of the trial by the Trial Steering Committee upon suggestion from the DMEC and request from the Trial Management Group will be that

evidence from the trial and from other sources suggests a) proof beyond reasonable doubt that for all, or for some types of patient, one treatment regimen is clearly indicated or contra-indicated in terms of a net difference in DFS or OS, and b) evidence that might reasonably be expected to influence routine clinical practice. Criteria for the above will usually be a difference in DFS or OS at any stage significant at $p < 0.001$ by overall log-rank analysis. Use of the Haybittle- Peto interim stopping criteria will not materially affect the overall alpha in the final analysis.

The DMEC will however reserve the right to release any data on outcome or side-effects through the Trial Steering Committee to the Trial Management Group (and if appropriate to participants) if it determines at any stage that the combined evidence from this and other studies justifies it.

MILESTONES

The recruitment rate will be between 1000 - 1350 patients per annum. Assuming an optimistic recruitment of 1350 per annum, then 4500 patients will be recruited at the end of the first 3 years. Patient enrolment began in December 2005, the completion of enrolment is planned for late 2008 /early 2009, and completion of relapse-free and survival status at 5 years is expected in early/mid 2013. The milestones assume projected event rates that may alter depending on the patient population recruited.

COMPLETION OF THE STUDY & DEFINITION OF STUDY END DATE

For the purposes of Clinical Trial Authorisation (CTA) and for Research Ethics Committee approval, the study end date is deemed to be the date of last data capture

Annual follow-up data will be collected for as long as the Trial Management Group consider it is contributing to the research question.

.

PREDICTIVE MARKER STUDIES

Detailed histopathological studies on tumour tissue will allow for potential analyses addressing the importance of conventional pathological factors (especially ER and PGR status), as well as a number of newer candidate predictive markers, using both conventional multivariate techniques, as well as neural network analysis. Similarly there is a pharmacogenomic study, with peripheral blood samples to be collected from all (consenting) patients. This will look at DNA polymorphisms in the genes responsible for metabolising the drugs administered, and link these to pharmacokinetic data in the subgroup of patients enrolled in that study, and to the toxicity and outcome in all patients.

COMPATIBILITY WITH OTHER STUDIES

The TACT2 Trial Management Group consider that patients may also be enrolled in the SOFT, SUPREMO, OPTION, REACT, IMPORT High, ALLTO, POETIC, MAPLE and Lapatinib Presurgery studies, providing of course they meet the inclusion criteria of these other studies. Patients who have had short duration (up to 28 days) pre-operative endocrine

treatment may also be enrolled, provided it is either given out with a study, or if within a study, without therapeutic intent. Compatibility with other studies that may open during the time that this trial enrols patients will be considered on a case by case basis by the Trial Steering Group, and in the light of the prevailing view of the MREC on compatibility (vide supra for relevant exclusion criteria).

RESEARCH GOVERNANCE

TRIAL ADMINISTRATION & LOGISTICS

Lothian Health Board and The Institute of Cancer Research are co-sponsors of the TACT2 Trial. Sponsorship activities and delegated responsibilities are shared between Lothian Health Board, the employer of the Chief Investigator; and The Institute of Cancer Research, in accordance with The Medicines for Human Use (Clinical Trials) Regulations 2004 and in line with the Research Governance Framework for Health and Social Care and ICH GCP. Both parties agree to allow inspection of sponsors' premises by the competent authorities.

Lothian Health Board has sponsorship responsibility for obtaining authorisation and appropriate ethics committee opinion (Part 3 of the Regulations) and for Pharmacovigilance (Part 5 of the Regulations). The following responsibilities have been delegated:

A to The Institute of Cancer Research:

1. Request clinical trial authorisation, amend the request;
2. Ensure an appropriate ethics opinion has been sought, and any amendments have been approved;
3. Give notice of amendments to CTA or protocol, make representations about amendments to the licensing authority;
4. Give notice a trial has ended;
5. Keep records of all serious adverse events reported by investigators;
6. Ensure recording and prompt reporting of serious adverse reactions to the Chief Investigator;
7. Report to the MHRA any serious adverse events which the chief investigator considers to be SUSARs;
8. Ensure investigators are informed of SUSARs;
9. Ensure all SUSARs including those in third countries entered into European database;
10. Provide annual list of SUSARs and a safety report.

The following responsibilities are retained by the Chief Investigator, or delegated in his absence, a named deputy:

11. Prompt decision as to which serious adverse events are SUSARs, and prompt reporting of that decision to the Section of Clinical Trials, ICR-CTSU, The Institute of Cancer Research for onward reporting to the licensing authority and Sponsoring Institutions.

B delegated to participating centres:

1. Ensure recording and prompt reporting of suspected unexpected serious adverse reactions (SUSARs) – delegated to participating centres;

The Institute of Cancer Research has responsibility for ensuring the research is conducted in accordance with Good Clinical Practice (Part 4 of the Regulations). The following responsibilities have been delegated:

A to Lothian Health Board:

2. Take appropriate urgent safety measures – delegated to the Chief Investigator, Lothian Health Board.

B delegated to participating centres:

1. Put and keep in place arrangements to adhere to the principles of GCP;
2. Keep a copy of all 'essential documents' (as defined under ICH GCP) and ensure appropriate archiving and destruction of documentation once the study has ended;
3. Ensure IMPs (investigational medicinal products) are made available to subjects free of charge;
4. Take appropriate urgent safety measures

Responsibilities are defined in an agreement between an individual participating centre and The Institute of Cancer Research.

The Institute of Cancer Research is responsible for administering funding and co-ordinating any required legal agreements and investigator statements.

The delegation of sponsorship responsibilities does not impact on or alter standard NHS indemnity cover. The agreement of delegated responsibilities is viewed as a partnership and as such it is necessary to share pertinent information between The Institute of Cancer Research and Lothian Health Board/Chief Investigator, including proposed inspections by the MHRA and/or other regulatory bodies.

PROTOCOL COMPLIANCE & MONITORING

TACT2 is being conducted in accordance with the professional and regulatory standards required for non-commercial research in the NHS under the EU Directive. Before activating the trial, participating centres are required to sign an agreement accepting sponsorship responsibility for all trial activity which takes place within their centre as stated in the Trial Administration and Logistics section above.

Staff from centres that have attended the Investigator Launch meeting will not require start-up visits unless they are requested by the Trials Unit or Principle Investigator.

DATA ACQUISTION & ON-SITE MONITORING/AUDITING

Trials Unit staff may visit centres to confirm that agreements are being adhered to, specifically to carry out source data verification and confirm compliance with the protocol and the protection of patients' rights as detailed in the Declaration of Helsinki. Copies of the Declaration may be obtained from the designated Trials Unit. By participating in the TACT2 trial the Principal Investigators at each centre are confirming agreement with his / her local NHS Trust to ensure that:

- sufficient data is recorded for all participating patients to enable accurate linkage between hospital records and CRFs
- source data and all trial related documentation are accurate, complete, maintained and accessible for monitoring and audit visits
- all staff at their centre who are involved with the trial will meet the requirements of the EU Directive
- original consent forms are dated and signed by both patient and investigator and are kept together in a central log together with a copy of the specific patient information sheet(s) given at the time of consent
- copies of CRF's are retained for 15 years to comply with international regulations
- staff will comply with the Standard Operating Procedures for TACT2

The affiliated Trials Unit will monitor receipt of CRFs and evaluate incoming CRFs for compliance with the protocol, inconsistencies and missing data.

Participating centres may be monitored by the Trials Unit and possibly by Health Authorities. Monitoring by Trials Units will confirm compliance with the protocol and source data verification (SDV).

Site auditing/monitoring will be conducted at a proportion of participating centres at least once during the course of the trial. If a monitoring visit is required the Trials Unit will contact the centre to discuss dates of proposed visit. Once a date has been confirmed a list of names of patients whose notes will be monitored / audited during the visit will be sent to the centre. This list will be sent out in advance to give sufficient time for the notes to be made available. (The Trial Statistician will decide the percentage of patients to be monitored / audited).

If any problems are detected in the course of the monitoring / auditing visits then the Principal Investigator and the Trials Unit will work together to resolve queries to determine the centre's future participation in the study.

ARCHIVING

All source and study documentation must be securely retained by the Principal Investigator for at least two years after the last approval of a marketing application in an ICH region and until there are no pending or contemplated marketing applications. Source data (including data on any patients who die) must be retained for the duration of the recruitment, treatment and follow up phases of the trial for inspection by representatives of ICR-CTSU or affiliated trials office, where these are different.

FINANCIAL MATTERS

The trial is investigator designed and led, and has been approved by CTAAC. It is endorsed by Cancer Research UK and meets the criteria for R&D support as outlined in the Statement of Partnership on Non-Commercial R&D in the NHS in England.

Research costs (to the clinical trials offices) are being funded by Cancer Research UK with additional funding in the form of educational grants provided by Roche, Amgen and Pfizer. If additional financial support is received from any other source, this will be made apparent to the approving MREC and CTAAC, but will not require a protocol amendment.

No individual per patient payment will be made to trusts or investigators, but NCRN (or regional equivalent) network resources should be made available as the trial is part of the NCRI portfolio by virtue of its approval by CTAAC.

CLINICAL RISK ASSESSMENT

Generic Risk Assessment Hazards to patients, study and organisation have been performed for TACT2 and have been considered low risk.

PUBLICATION POLICY

The main trial results will be published in the name of the trial in a peer-reviewed journal, on behalf of all collaborators. The manuscript will be prepared by a writing group, appointed from amongst the Trial Management Group, representatives of the regional trials groups, and high accruing clinicians. The trials offices and all participating centres and clinicians will be acknowledged in this publication. All presentations and publications relating to the trial must be authorised by the Steering Group. There will hopefully be secondary publications relating to the detailed toxicity and Quality of Life study, and the various biological studies. The authorship on these secondary publications will reflect the intellectual and time input into these studies, and will not be the same as on the primary publication. No investigator may present or attempt to publish data relating to TACT 2 without prior permission from the Trial Management Group.

CONFIDENTIALITY & LIABILITY

LIABILITY / INDEMNITY / INSURANCE

This study is an investigator-led trial endorsed by the Clinical Trials Awards and Advisory Committee (CTAAC) of Cancer Research UK and the UK Medical Research Council. Indemnity for participating hospitals is provided by the usual NHS indemnity arrangements.

PATIENT CONFIDENTIALITY

The patient's full name, date of birth, hospital number and NHS number (CHI number in Scotland) will be collected at randomisation to allow tracing through national records and to assist with long term follow-up. The personal data recorded on all documents will be

regarded as confidential, and to preserve each subject's anonymity, only their initials and date of birth will be recorded on subsequent Case Report Forms.

The investigator must keep a separate log of patients' trial numbers, names, addresses and hospital numbers. The investigator must maintain in strict confidence trial documents, which are to be held in the local hospital (e.g. patients' written consent forms). The investigator must ensure the patient's confidentiality is maintained.

ICR-CTSU and all other participating trials offices will maintain the confidentiality of all subject data and will not reproduce or disclose any information by which subjects could be identified, other than reporting of serious adverse events. Representatives of the trials offices will be required to have access to patient notes for quality assurance purposes but patients should be reassured that their confidentiality will be respected at all times. (In the case of special problems and / or government queries, it is also necessary to have access to the complete study records, provided that patient confidentiality is protected).

ETHICAL CONSIDERATIONS

The study has been approved by MREC for Scotland. Before entering patients, the Principal Investigator at each site is responsible for gaining Site Specific Assessment and advising the main REC. Patients should be asked to sign the main consent form and the consent form for the biological studies after having both verbal and written information. Patients who do not wish to take part in one or either of the biological studies may take part in the main trial. Patients participating in the Quality of Life Study must also sign the Quality of Life consent form. All consent forms must be countersigned by the Principal Investigator or a designated individual, and a record of who designated individuals are and the circumstances under which they countersign consent forms must be clearly documented at the research site and be available for inspection together with original copies of all signed patient consent forms.

The TACT2 patient information sheet should be provided in addition to the standard chemotherapy patient information sheets that are provided by the centre and used in routine practice.

WITHDRAWAL OF PATIENTS FROM STUDY TREATMENT

Patients who do not receive their allocated treatment for any reason should be treated at the discretion of their clinician. However, analyses of all outcome data will be on the basis of intention to treat. Unless the patient requests otherwise, all CRFs, including long term follow up, should be completed, regardless of treatment actually received. A trial deviation form should be completed to record details of deviation from treatment allocation, and also for any patient who withdraws consent for further follow up. Patients are asked prior to randomisation to consent to follow up should they withdraw from the treatment allocation (see patient information sheet and consent form), and any patient unwilling to give that assurance prior to trial entry should not be randomised. Patients are, however, free to reverse that decision at any time without giving a reason.

REFERENCES

1. CRUK, Cancer Statistics. www.cancerresearchuk.org, 2003.
2. Hudis, C., New approaches to adjuvant chemotherapy for breast cancer. Pharmacotherapy, 1996. 16(3 Pt 2): p. 88S-93S.
3. Irvin, R.J. and J.G. Kuhn, Financial considerations in the use of adjuvant chemotherapy. Cancer Treat Res, 1992. 60: p. 207-22.
4. Bishop, J.F. and K. Macarounas-Kirchman, The pharmacoeconomics of cancer therapies. Semin Oncol, 1997. 24(6 Suppl 19): p. S19-106-S19-111.
5. Annemans, L., et al., Cost-Effectiveness of the Sequential Addition of Taxol (T) to Doxorubicin Plus Cyclophosphamide (AC) in the Adjuvant Chemotherapy of Patients with Node-Positive Breast Cancer. ASCO Proc, 2000. 19: p. 1752A.
6. Brinkley, D. & J.L. Haybrittle, The curability of breast cancer. Lancet, 1975. 2(7925): p. 95-7.
7. Adair, F., et al., Long-term follow-up of breast cancer patients: the 30-year report. Cancer, 1974. 33(4): p. 1145-50.
8. Schabel, F., Concepts for the successful treatment of micrometastases. Cancer, 1975. 35: p. 15-24.
9. Fisher, B., Surgical adjuvant therapy for breast cancer. Cancer, 1972. 30(6): p. 1556-64.
10. EBCTCG, Polychemotherapy for early breast cancer: an overview of the randomised trials. Early Breast Cancer Trialists' Collaborative Group. Lancet, 1998. 352(9132): p. 930-42.
11. Levine, M.N., Bramwell VH, Pritchard KI, et al., Randomized trial of intensive cyclophosphamide, epirubicin, and fluorouracil chemotherapy compared with cyclophosphamide, methotrexate, and fluorouracil in premenopausal women with node-positive breast cancer. National Cancer Institute of Canada Clinical Trials Group. J Clin Oncol, 1998. 16(8): p. 2651-8.
12. Bonneterre, J. Roché H, Bremand P et al., Results of a randomized trial of adjuvant chemotherapy with FEC 100 in high risk node-positive breast cancer patients. French Adjuvant Study group (FASG) ASCO Proc, 1998. 17: p 473A
13. Poole CJ., Dunn JA., et al., NEAT (National Epirubicin Adjuvant Trial) and SCTBG BR9601 (Scottish Cancer Triaols Breast Group) phase III adjuvant breast trial shows a significant relapse-free and overall survival advantage for sequential ECMF (abstract). ASCO Proc, 2003 22:4
14. Wunderlich, A K and Schmalenberg, H S. Practicability and acute haematological toxicity of 2- and 3-weekly CHOP and CHOEP chemotherapy for aggressive non-Hodgkin's lymphoma: results from the NHL-B trial of the German High-Grade Non-Hodgkin's Lymphoma Study Group (DSHNHL) Ann Oncol 2003;14:881-893
15. Woll, P J, Thatcher, N, Lomax, L, Hodgetts, J, Lee, S M, Burt, P A, Stout, R, Simms, T, Davies, R, Pettengell, R, Thatcher, N, Girling, D J, Hopwood, P, Sambrook, R J, Qian, W, Stephens, and RJ. Use of hematopoietic progenitors in whole blood to support dose-dense chemotherapy: a randomized phase II trial in small-cell lung cancer patients Improving survival without reducing quality of life in small-cell lung cancer patients by increasing the dose-intensity of chemotherapy with granulocyte colony-stimulating factor support: results of a British Medical Research Council Multicenter Randomized Trial. Medical Research Council Lung Cancer Working Party J Clin Oncol 2001;19:712-719
16. Huisman, C, Biesma, B, Postmus, P E, Giaccone, G, Schramel, F M, Smit, E F, Woll, P J, Thatcher, N, Lomax, L, Hodgetts, J, Lee, S M, Burt, P A, Stout, R, Simms, T, Davies, R, Pettengell, R, Thatcher, N, Girling, D J, Hopwood, P, Sambrook, R J, Qian, W, Stephens, and RJ. Accelerated cisplatin and high-dose epirubicin with G-CSF support in patients with relapsed non-small-cell lung cancer: feasibility and efficacy Use of hematopoietic progenitors in whole blood to support dose-dense chemotherapy: a randomized phase II trial in small-cell lung cancer patients Improving survival without reducing quality of life in small-cell lung cancer patients by increasing the dose-intensity

of chemotherapy with granulocyte colony-stimulating factor support: results of a British Medical Research Council Multicenter Randomized Trial. Medical Research Council Lung Cancer Working Party Br J Cancer 2001;85:1456-1461

17. Citron ML., Berry DA., Cirrincione C., et al., Randomized trial of dose-dense versus conventionally scheduled and sequential versus concurrent combination chemotherapy as postoperative adjuvant treatment of node-positive primary breast cancer: first report of Intergroup Trial C9741/Cancer and Leukemia Group B Trial 9741. J Clin Oncol 2003 21:1431-9.
18. Oshaughnessy, J A, Blum, J, Moiseyenko, V, Jones, S E, Miles, D, Bell, D, Rosso, R, Mauriac, L, Osterwalder, B, Burger, H U, and Laws, S. Randomized, open-label, phase II trial of oral capecitabine (Xeloda) vs. a reference arm of intravenous CMF (cyclophosphamide, methotrexate and 5-fluorouracil) as first-line therapy for advanced/metastatic breast cancer Ann Oncol 2001;12:1247-1254
19. Blum, J J, Buzdar, A U, LoRusso, P M, Kuter, I, Vogel, C, Burger, H U, Brown, C, and Griffin, T. A multicenter phase II trial of Xeloda (capecitabine) in paclitaxel-refractory metastatic breast cancer (MBC) Proc ASCO 1998;17:125-(abstr 476)
20. Toi, M and Ohno, S. The role of thymidine phosphorylase (TP) in choosing FU/capecitabine chemotherapy Proc ASCO 2003;22:34-abstract No 137

21. Hudis, C, Barlow, W, Costantino, J, Gray, R, Pritchard, K, Chapman, JA, Sparano, J, Hunsberger, S, Enos, R, Gelber, D, Zujewski, JA. Proposal for Standardized Definitions for Efficacy End Points in Adjuvant Breast Cancer Trials: The STEEP System. J Clin Oncol 2007 25:2127-2132.

APPENDIX 1 Sub-study 1: Quality of Life & Toxicity

Reasons for stopping recruitment and planning to open a new QL study

The QL Study as previously planned was challenging to manage because of the critical time points of the follow up postal questionnaire booklets sent out by the QL coordinator, in particular the on-treatment 1st phase (6 weeks) and the end of treatment 1st phase (8-12 weeks in accelerated and standard arm respectively). The fast recruitment into the main study and QL study added additional challenges, increasing the intensity and volume of work. As a result we found significant deviations from the planned assessment times due to the short time frame available to the patients for completion. At each time point, only about 35-45% of those who returned the questionnaire, completed it within the planned time frame.

In order not to compromise the scientific validity of the study, as incorrect timing of QL assessments in chemotherapy oncological trials can jeopardise both the reliability of the QL findings within treatment and the validity of QL outcome comparisons between treatments, the design of the timing of QL assessments in the study have been revised. The main difference is simplifying the schedule of data collection. Patients will complete the QL questionnaires at baseline, end of 1st treatment phase, end of 2nd treatment phase, at 12 and 24 months after randomisation. The second phase of the QL sub-study will aim to recruit further new 1,000 patients.

Patients already recruited in the first phase of QL sub-study between December 2005 and March 2007 (n=778) will continue to receive booklets at the appropriate follow-up time points. Their data, cannot be used for the primary QL analysis, as assessment times of the two phases of the study will be different, but all the data collected will be used for other analyses of equal scientific interest, and for this reason we would want to complete the data collection from patients who have already enrolled on the initial QL schedule. For example as QL assessments coincide with the occurrence of relevant symptoms, the availability of data reflecting functioning, symptoms and global health status of patients at different time point will permit:

- an estimation of the nature and magnitude of the error produced by the ”incorrect” timing the QL assessments in patients receiving accelerated and standard chemotherapy schedule, E-CMF and E-X.
- comparison of changes in QL outcomes overtime in the two phases of the QL study. This will also confirm the validity of the timing used.

The revised second phase of the QL study is described below.

Background

To inform patients of the options available in the adjuvant treatment of early breast cancer, not only is it important to know the survival benefits of systemic therapy, but also the impact such therapy will have on their quality of life (QL). For example, in the Canadian NCIC study of CEF versus CMF, the more active treatment was associated with more toxicity but the additional impact of this upon the patients’ QL disappears within a few months of completing adjuvant treatment [1].

Fatigue has been recognised as a significant and debilitating side-effect of chemotherapy, which in many patients can persist for a considerable time after completion of treatment and can have a major impact on functioning and psychological recovery [2,3]. The possibility that there may be differences in fatigue across treatment modalities has been suggested, comparing chemotherapy and radiotherapy with radiotherapy alone [4]. Women who received chemotherapy and radiotherapy had greater fatigue severity and disruptiveness than women receiving radiotherapy alone. It can be hypothesised that different chemotherapy drugs, different intensity and duration of adjuvant chemotherapy may have different impact on fatigue. For example, dose-intense chemotherapy (CEF14) induced a higher, though transient psychological distress when compared with CEF21 [5]. Therefore, more detailed evaluation of fatigue proposed in TACT2 will help us to examine these effects.

Rationale

To compare QL in each of the 4 treatment groups. With the exception of a trial specific evaluation of the impact of toxicities, similar to that used for the TACT trial, all other instruments to be used are validated questionnaires.

Assessments during treatment will compare the impact on QL of:

- Accelerated treatment versus standard treatment. Accelerated epirubicin is expected to be more effective treatment, but it is not known what is the impact on QL.
- Capecitabine versus CMF after completing 4 courses of epirubicin. The hypothesis is that capecitabine will be equally effective as CMF regimen but less toxic with less impact on QL.

Follow up assessments completed after treatment aim to determine if and when QL returns to baseline levels.

Design

The Quality of Life questionnaires to be used are:

- EORTC QLQ-C30 (version 3) [6]
- EORTC-Breast Cancer Questionnaire (EORTC QLQ-BR23) [7]
- Hospital Anxiety & Depression [8]
- Trial specific evaluation of impact of toxicities on QL
- EuroQoL [9]
- Fatigue questionnaire - Fatigue Symptom Inventory (FSI) and Wu Cancer Fatigue Scale (WCFS) [10-12]

Procedure

Baseline assessments

All patients in the QL study should complete a baseline questionnaire booklet, which incorporates a demographic form, in clinic after giving informed consent, but before randomised treatment allocation is known. The completed questionnaire booklet should be posted to the QL coordinator as soon as the patient is randomised and the patient’s trial number is known.

In order to get the precise date of administration of the first cycle of chemotherapy in all patients in the QL study, nurses will send a postcard to the QL centre for each patient in the QL sub-study containing the trial ID number, treatment arm (as a check) and date of administration of their first cycle. No other patient identification data will be contained to permit anonymity.

All subsequent questionnaire booklets (i.e. those due for completion during and after chemotherapy treatment) will be sent out by post to patients' home addresses (as supplied on the demographic forms) by the QL coordinator based at CaCTUS in Edinburgh.

Assessments during the treatment phase

Timing

Each patient should complete 2 questionnaire booklets during chemotherapy treatment. These should be at the end of epirubicin treatment, immediately before switching to the second phase and at the end of the second phase (either CMF or capecitabine). The timing of chemotherapy cycles varies depending on treatment allocation, and the weeks during which patients receive active chemotherapy treatment will vary accordingly, as shown below:

Week during which active chemotherapy treatment is given:

	1	2	3	4	5	6	7	**8**	9	10	11	**12**	13	14	15	16	17	18	19	**20**	21	22	**23**	**24**	25	26	**27**
Standard E-CMF	E			E			E			E			CMF	CMF			CMF	CMF			CMF	CMF			CMF	CMF	
Accelerated E-CMF	E		E		E		E		CMF	CMF			CMF	CMF			CMF	CMF			CMF	CMF					
Standard E-X	E			E			E			E			X	X		X	X		X	X		X	X				
Accelerated E-X	E		E		E		E		X	X		X	X		X	X		X	X								

Shaded boxes are those weeks in which QL assessments are to be made.
E = epirubicin
CMF = cyclophosphamide, methotrexate and 5-FU
X = Xeloda (capecitabine)

Purpose and precision of timing for each assessment

Assessment time point	What the assessment aims to compare	Precise timing for patients to complete assessments
End of 1st phase<br>Week 8 (if accelerated)<br>Week 12 (if standard)	Impact of schedule intensity after treatment.<br><br>Also a baseline for comparison with the assessment during second phase of chemotherapy.	The week before day 1 of cycle 5 of chemotherapy (ie last week of cycle 4). Ideally completion should be on last day of this week, but a window of 1 week is acceptable.
End of 2nd phase*<br>Week 20 (if accelerated & capecitabine)<br>Week 23 (if accelerated & CMF)<br>Week 24 (if standard & capecitabine)<br>Week 27 (if standard & CMF)	Impact on QL of capecitabine compared with CMF*. Within-patient comparisons of QL during 1st and 2nd phases of chemotherapy.	The week after chemotherapy was completed (i.e. week 3 of last chemotherapy cycle)

* The difference in time of QL administration in the four arms will be taken into account in the statistical analysis. Detailed methods will be specified in the Statistical Analysis Plan.
For patients who fail to complete eight cycles of chemotherapy, the QL booklets will still be administered at the same expected time-point as they would have been had chemotherapy been delivered as per protocol, in order to avoid any bias due to data collection at different time-points.

After the end of chemotherapy treatment QL will be measured at 12 months after randomisation (see below), which allows for all radiotherapy to have also been given, and this time point will act as a check as to the proportion of patients whose QL has reverted to baseline levels after the various treatments. This will also accommodate delays in chemotherapy administration due to toxicity.

Final QL assessment will be 24 months after randomisation.

Patients who complete QL assessments outside the specified timescale will be included in the QL analysis on an “intention to complete” basis. A second analysis will be performed that excludes QL data that has been completed at least one chemotherapy administration outside the intended timeframe, or in the case of the post-cycle 8 of chemotherapy assessment, after any radiotherapy has been started.

Assessments during the follow up phase

These are at 12 months and 24 months after randomisation. The QL coordinator will contact patients' GPs and / or hospital clinic staff before booklets are sent to patients to confirm that they are alive and well enough to receive them.

Any patient scoring 19 or more at any time point on the combined HADS anxiety/depression scale is at risk of significant psychological morbidity. The patient's oncologist and/or GP will be informed should this occur.

Responsibilities of participating centres

Individual centres may opt to participate in the QL Study. Within those centres, all patients invited to take part in the main trial and able to complete the QL questionnaires (i.e. able to read and understand English) should be invited to take part in the QL study. However, patients may decline entry into the QL Study but still participate in the main study.

Baseline:	Patients must complete the baseline booklet before the treatment allocation is known. The demographic form should be completed by the patient, and the clinic nurse should ensure that treatment details are recorded on the questionnaire booklet, and the trial number is recorded on both the baseline booklet and the demographic form. Both the booklet and demographic form should be sent to the QL coordinator immediately after randomisation.
Treatment phase:	Pre-printed freepost postcards provided by the QL coordinator should be completed and returned to the QL coordinator on day 1 of cycle 1, and on day one of each cycle immediately preceding the next QL assessment. These cards are to confirm the exact date of day 1 of the chemotherapy cycle immediately preceding the next QL assessment. Receipt of a pre-printed card will also be taken as confirmation that a patient is fit and well enough to receive the next QL assessment. Principal investigators are responsible for informing the QL coordinator of any patient unable to complete further questionnaires because they are unfit to receive them or because of treatment related deaths.
Follow up phase:	the QL coordinator will contact participating centres just before follow up assessments are due to confirm that patients are fit and well enough to receive them.

The Principal Investigator at centres opting to participate in the QL study should ensure that staffing allows for the above responsibilities to be met.

Sample size

The detailed sub-study will aim to include 1000 patients and has been powered according to the requirements for the QL component of the analysis. It is believed however that this number is sufficient to provide reliable estimates of toxicity and health services resource use. 1000 patients entered should provide complete–case data on over 800-850 patients i.e. 80%-85% of patients will complete the 12 month assessment (based on the TACT trial compliance figures). It is possible that there will be some carry-over effect between the treatments therefore power has been calculated to look at 4 separate groups of 200-213 patients completing the 12 month assessment. This will provide 92%-94% power to detect a difference of 20% or more (e.g. from 40% to 60% or 45% to 65%) in any proportion at the 1% significance level. Differences of 18% could be detected with 82% power. If there were

no carry over effect and it was possible to look at treatments combined there will be 99% power to detect a difference of 20% or more (e.g. from 40% to 60% or 45% to 65%) in any proportion at the 1% significance level. Differences of 13% could be detected with 82% power. Differences of 5 points or greater in QL scores between the E-CMF and accelerated treatment are considered clinically relevant. A mean difference of 5 points with a standard deviation of approximately 19 (consistent with preliminary data from the TACT QL study) would equate to a standardised difference of 0.27. The 800 - 850 patients in this comparison (400 or 425 in each arm) could detect a difference of 0.27 or more with at least 90% power (alpha = 0.01). If a smaller standard deviation were observed the detectable standardised difference would be larger and hence the power of the study will be greater than 90%.

The intention of the second phase of the QL sub-study is to recruit 1000 patients, which will give 90% power to detect a difference of 12% or more in any proportion at the 1% significance level. The type 1 error chosen, allows, to some degree, for multiple testing involved in analysing individual sub-scales of the QL questionnaires. If it appears that it will be possible to recruit more than 1000 patients in the second phase of the QL sub-study, MREC will be contacted to seek permission to continue recruitment beyond that figure if it appears feasible.

The primary analysis in the QL Study will compare the overall QL and HADS scores. The time dependency of the data will be acknowledged by using a generalised linear modelling approach. Missing data will be handled according to recommended standard EORTC procedures.

References

1. Levine MN, Bramwell VH, Pritchard KI, Norris BD, Shepherd LE, Abu-Zahra H, Findlay B, Warr D, Bowman D, Myles J, Arnold A, Vandenberg T, MacKenzie R, Robert J, Ottaway J, Burnell M, Williams CK, Tu D. Randomized trial of intensive cyclophosphamide, epirubicin, and fluorouracil chemotherapy compared with cyclophosphamide, methotrexate, and fluorouracil in premenopausal women with node-positive breast cancer. National Cancer Institute of Canada Clinical Trials Group.J Clin Oncol. 1998; 16(8):2651-8.
2. Broeckel JA, Jacobsen PB, Horton J, Balducci L, Lyman GH. Characteristics and correlates of fatigue after adjuvant chemotherapy for breast cancer. J Clin Oncol. 1998; 16(5):1689-96.
3. Fan HG, Houede-Tchen N, Yi QL, Chemerynsky I, Downie FP, Sabate K, Tannock IF. Fatigue, menopausal symptoms, and cognitive function in women after adjuvant chemotherapy for breast cancer: 1- and 2-year follow-up of a prospective controlled study. J Clin Oncol. 2005; 23(31):8025-32.
4. Donovan KA, Jacobsen PB, Andrykowski MA, Winters EM, Balducci L, Malik U, Kenady D, McGrath P. Course of fatigue in women receiving chemotherapy and/or radiotherapy for early stage breast cancer. J Pain Symptom Manage. 2004; 28(4):373-80.
5. Del Mastro L, Costantini M, Morasso G, Bonci F, Bergaglio M, Banducci S, Viterbori P, Conte P, Rosso R, Venturini M. Impact of two different dose-intensity chemotherapy regimens on psychological distress in early breast cancer patients. Eur J Cancer. 2002; 38(3):359-66.
6. Aaronson NK et al. The European Organisation for the Research and Treatment of Cancer QLQ-C30: A quality of life instrument for use with international clinical trials in oncology. J Nat Cancer Inst. 1993; 85:365-376

7. Sprangers MAG Groenvold M Arraras JI Franklin J Velde A Muller M Franzini L Williams A de Haes HCJM Hopwood P Cull A Aaronson NK. The European Organisation for Research and Treatment of Cancer Breast Cancer-Specific Quality-of–Life Questionnaire Module: First results from a three-country field study. J Clin Oncol. 2001; 5:917-929
8. Zigmond AS & Snaith RP . The Hospital Anxiety and Depression Scale. Acta Psychiatr Scand 1983; 67:361-370.
9. EuroQoL Group. EuroQoL - a new facility for the measurement of health-related quality of life. Health Policy 1990; 16: 199-208
10. Hann DM, Denniston MM, Baker F. Measurement of fatigue in cancer patients: further validation of the Fatigue Symptom Inventory. Qual Life Res. 2000; 9(7):847-54.
11. Wu HS, McSweeney M. Assessing fatigue in persons with cancer: an instrument development and testing study.Cancer. 2004; 101(7):1685-95.
12. Wu HS, Wyrwich KW, McSweeney M. Assessing fatigue in persons with cancer: further validation of the Wu Cancer Fatigue Scale. J Pain Symptom Manage. 2006; 32(3):255-65.

APPENDIX 2 Sub-study 2: Biology

Paraffin blocks

Standard pathological information will be collected at randomisation on all patients entered into the trial. This information will also include the histology number, location of paraffin tumour blocks and reporting consultant pathologist (if known). This will enable the prospective construction of tissue microarrays (TMAs) for storage and analysis at a later date. This method of collection has already proved successful in the Taxotere as Adjuvant Chemotherapy (TACT) Trial. Tissue sections will be tested using immunohistochemical techniques for the presence of standard biological predictive markers of treatment benefit such as EGFR, HER-2 and p53.

There are a number of other hypotheses which can be tested in the bank of TMAs obtained from patients in this trial, relating to markers predicting for potential sensitivity to the agents used in this trial, such as Thymidine Phosphorylase and Topoisomerase IIα. However other studies are currently in progress that will refine such hypotheses, and therefore it is felt that it would not be appropriate to define them in precise terms at this stage.

Blood samples

A single blood sample will be collected and stored at -80°C at a central laboratory for future screening of DNA polymorphisms in all (consenting) patients. DNA will be prepared from the samples and can be used to look for polymorphisms in the genes encoding the enzymes that metabolise and/or activate the chemotherapeutic agents administered. For patients in the pharmacokinetic sub-study, validation will be carried out by comparing the observed Pharmacokinetics with the DNA polymorphisms. For all patients, the polymorphisms will be compared with the observed toxicity, hypothesising that variation in DNA sequence that might be predictive of those that suffer more extreme toxicity, perhaps even identifying those for whom certain drugs should be avoided. Once outcome data are available, the data will then be analysed in conjunction with the observed benefits from adjuvant capecitabine and/or accelerated treatment, controlling for standard recognised risk factors. This has the potential to define a subset of patients with the most or the least to gain from the trial treatments.

All patients will be invited to take part in both aspects of the biological studies, but may still enter the main trial if they do not want to participate in the biological sub studies.

APPENDIX 3 Sub-study 3: Cost Consequences of Trial Participation

The introduction of capecitabine and / or GCSF in the TACT2 trial of adjuvant treatment for breast carcinoma involves additional cost of some drugs, but trial drug costs have the potential to be offset by the reduced cost of treating treatment related toxicity and relapsed disease.

It is anticipated that oral administration of capecitabine therapy will provide advantages over IV administration of CMF with respect to medical resource utilization, due to avoidance of clinic cost for staff time, IV supplies, administration time, etc. In particular, oral therapy may provide a significant advantage with respect to resource utilization for patients whilst on oral cytotoxic therapy once they have completed their IV chemotherapy regimen, since the oral capecitabine therapy will likely require less hospital visits and/or visits of shorter duration than the IV therapy.

Estimated NHS costs on which to base hypotheses

Estimate of added NHS treatment costs for patients in this study (estimates correct as of December 2003).

ARM	No. of out-patient chemo visits	Drug costs	Estimated rate of Neutropenic sepsis
E-CMF (as per NEAT)	12	Standard	13%
E(a)-CMF	12	+£840	3% + 6% = 9%
E-X	8 + 1 toxicity check	- £350	6% + 2% = 8%
E(a)-X	8 + 1 toxicity check	+£ 840-£350	3% + 2% = 5%
**Average**	**10.5 visits**	**£245 per patient**	**8.75%**

Data:

Cost of one dose of Neulasta (Pegylated GCSF)	£840 (next 3 free)
Estimated cost of Classical CMF (@Beatson Oncology Centre)<br>(Not including giving sets etc.)	£350
Estimated cost of admission for neutropenic sepsis<br>(Heather Dalrymple, WGH Pharmacy, Edinburgh)	£1440
Drug costs of neutropenic sepsis per patient admitted	£425

Neutropenic sepsis rate for 8 cycles of E-CMF was 13% in Neat trial, and 10% for 6 cycles of CMF. These data suggest that the rate is around 1.5% per cycle. Accelerated chemotherapy appears to reduce this by 50% in CALGB 9741, which would give a rate of 0.75% per cycle of accelerated epirubicin. Capecitabine is associated with an extremely low rate even in advanced disease, but allowing for possible admissions for diarrhoea, we have estimated the rate at 0.5% per cycle.

Thus on average enrolling patients in this trial reduces the number of visits by 1.5, requires an extra £245 of direct drug costs, and reduces the incidence of neutropenic sepsis by perhaps 4.25%, providing a direct saving of around £18 per patient, and additional release of resources of £50 per patients in reduced bed costs, plus the reduced day case requirement. This does not include the cost of GCSF in the non-accelerated arms and pegylated GCSF will also be available at a price of £840 for the first dose with the next 3 doses free. The frequency of such use is not known, but would be a cost benefit to trusts that would need to be offset against the slight increase in total drug costs for patients in this study.

Therefore we estimate that the extra costs to institutions to be around £230 per patient in direct drug costs. For a trust recruiting perhaps 40 patients per year this equates to approximately £10, 000 per annum. The value of resource released will approximate to at least £3,000 for the same number of patients (excluding transport costs).

Health Economic Evaluation

A health economic evaluation will be carried out after the main trial has completed recruitment, and will be the subject of a separate funding application. Data collection is prospective and incorporated in the trial design but the extent of the economic analysis will be dependent on clinical outcome of the trial. It will take the form of a cost-consequences analysis and of a cost-effectiveness analysis. In the former, the differential resource use and cost of the alternative management strategies will be presented alongside the range of clinical and health-related quality of life (HRQL) effects. In the latter, the differential cost of the alternative treatments will be related to their differential benefits in terms of quality-adjusted life years (QALYs), and standard cost-effectiveness acceptability curves will be used to show the probability of one option being more cost-effective than the other.

Estimating resource use

Resource use measurement during the trial will be collected in a similar fashion to the FOCUS trial [1] and is divided into four components: hospital; NHS non-hospital; patient travel costs and patient productivity costs. These are dealt with in turn below.

Hospital resource use

The dominant costs in chemotherapy treatment are likely to be inpatient stay and high cost drugs [2]. There are potentially different rates of inpatient stay related to toxicity of treatment in this trial, and potential differences in admissions for management of recurrent disease depending on the clinical outcome of the trial. These costs are being collected on all patients in trial through the chemotherapy details, the adverse event reporting of admissions and the questions as to admissions on the annual follow up reports. Data collected will include stays in hospital related to non-study hospitals.

Because capecitabine is taken at home and requires fewer hospital attendances then data on patient travel distances, economic circumstances and whether patients are accompanied on hospital visits will be collected on those patients in the quality of life study enabling an economic evaluation with a societal perspective.

NHS non-hospital resource use

Patients' use of community-based NHS services will be collected from patients participating in the Quality of Life study in the form of a short questionnaire incorporated in the Quality of Life questionnaire booklets administered during treatment and follow up. The resources will include visits to and from a GP or district nurse.

Patient travel costs

There will be differences in hospital visits between trial arms. Patients' travel costs will be estimated using a cost per hospital visit and multiplying that cost by the number of occasions each patient visits hospital. In order to cost a given visit to hospital for each patient, a short questionnaire will be administered at baseline. This will collect information on the typical mode(s) of transport, distance and time of journeys to hospital, and whether the patient had a companion. Based on these data, patients' travel costs will be based on published unit costs for travel.

The questionnaire will also collect information to cost the time patients and any companions allocate to the visit.

Patient productivity costs

The number of days during which patients are unable to undertake their usual activities because of illness will be established at the various points of follow-up. In addition, it will be necessary to ask patients at baseline what their usual activity is.

Measuring effects

The clinical trial is estimating a range of clinical and HRQL effects in trial patients. The purpose of the economic evaluation will be to set these in context of the resource costs incurred in achieving them. Cumulative costs will be shown in the form of a timeline from randomization. A cost-effectiveness analysis will relate differential cost to an aggregated measure of effect in the form of a quality-adjusted life-year (QALY).

References:

1. FOCUS 1 and FOCUS 2 Clinical Protocol MRC Clinical Trials Unit – professor Mark Sculpher, Advisor Economic Evaluation.
2. Bloomfield DJ. Krahn MD. Neogi T. Panzarella T. Smith TJ. Warde P. Willan AR. Ernst S. Moore MJ. Neville A. Tannock IF. Economic evaluation of chemotherapy with mitoxantrone plus prednisone for symptomatic hormone-resistant prostate cancer: based on a Canadian randomized trial with palliative end points. [Clinical Trial. Phase III. Journal Article. Multicenter Study. Randomized Controlled Trial] Journal of Clinical Oncology. 16(6):2272-9, 1998 Jun.

APPENDIX 4 Sub-study 4: Evaluation of Cardiac Function

Anthracyclines are well known to be cardiotoxic, although milligram for milligram epirubicin is less so than adriamycin [1]. The risk factors for cardiotoxicity are increasing age, high BMI, pre-existing cardiac disease and cumulative dose of anthracycline. Dose intensity within the range of 25 mg/week and 35 mg/week has not been shown to be an issue in retrospective studies [2].

Capecitibine also has cardiac effects, but these relate to induction of coronary artery spasm rather than direct myocycte toxicity as with the anthracyclines. Nevertheless if pre-existing coronary artery disease is present such spasm may be sufficient to induce myocardial injury. The cardiac effect of capecitibine after anthracyclines is not known, although anthracyclines may cause increases in thymidilate synthetase levels increasing the potency of capecitibine.

The TACT2 trial gives an opportunity to prospectively study the cardiac effects of epirubicin dose intensity and also refine the relative contributions of other potential variables which may affect cardiotoxicity.

Methods

Centres which have routinely performed pre-chemotherapy LVEF estimations will repeat these at 2.5 – 3 years post-randomisation. Other data that will be collected is as follows:

- Age, smoking history, BMI will be available from the existing CRFs
- Blood pressure pre- first cycle of chemotherapy will be obtained from the nursing records
- Cardiac history and medications will be obtained from the case notes, and rechecked at the time of ordering the repeat LVEF, BMI will be re-calculated at that time

Patients who subsequently received adjuvant Herceptin will be included but analysed as a separate group.

The risk of developing any degree of cardiac problem as defined by the NY scale will be analysed by treatment arm. Single and Mulivariate anaylsis will be performed to also include known non-chemotherapy risk factors: age, pre- and post- chemotherapy BMI, smoking history, hypertension, use of adjuvant Herceptin.

Any patient known to have died with or due to cardiac illness will also be included in the analysis.

References

1. Von Hoff DD. Layard MW. Basa P. Davis HL Jr. Rozencweig M. Muggia FM. Risk factors for doxorubicin-induced congestive heart failure. Annals of Internal Medicine. 91(5):710-7, 1979 Nov.
2. Fumoleau, P. Roche, H. Kerbrat, P. Bonneterre, J. Romestaing, P. Fargeot, P. Namer, M. Monnier, A. Montcuquet, P. Goudier, M-J. Luporsi, E. French Adjuvant Study Group. Long-term cardiac toxicity after adjuvant epirubicin-based chemotherapy in early breast

cancer: French Adjuvant Study Group results. Annals of Oncology. 17(1):85-92, 2006 Jan.

APPENDIX 5 Radiotherapeutic Procedures

1 General/Timing

Irradiation should be postponed until systemic treatment is completed. Ideally, it should commence 4 weeks after the last cycle of chemotherapy commences, or after any planned post-chemotherapy surgery. However, it should start no later than 6 weeks after the last cycle of chemotherapy.

2 Radiotherapy indications

2.1 Chest wall radiotherapy

Chest wall radiotherapy following mastectomy should be considered for patients who fit any one of the following criteria [39]:

- T3 tumours
- Four or more axillary nodes involved
- Involved margins
- 1 – 3 nodes involved with the addition of any one of:
    - Lymph-vascular invasion
    - High grade tumours
    - Patients otherwise eligible for SUPREMO would be allowed to receive XRT or not as determined by that randomisation

2.2 Radiotherapy to the breast itself

This is an integral part of any breast-conserving procedure and should be performed in all cases.

2.3 Nodal radiotherapy

Radiotherapy must include the axilla if an axillary sample has been positive and a full (usually level III) surgical clearance has not been performed. In these cases, it is strongly recommended that a treatment technique is used which minimises any overlap, and that the match interface should not involve the axilla, a potential disease site.

Radiotherapy to the axilla after a full level III axillary dissection must be avoided unless there is evidence of macroscopic residual disease in the axilla.

Irradiation of internal mammary nodes should be avoided so as to minimise the radiation dose to myocardium and lung.

Extracapsular spread in patients with involved axillary nodes does not constitute an absolute indication for axillary radiotherapy after surgical clearance of the axilla, given the higher risks of lymphoedema in these circumstances, and the lack of any evidence of survival benefit. Any treatment must only be considered after careful discussion with the patient on an individual patient basis.

Another controversial area is the case for a supraclavicular field in patients with more than three axillary nodes involved, especially, perhaps, those with apical node involvement. However, we feel it would be inappropriate to proscribe these practices and it is recognised such decisions have to be made on a case-by-case basis. If fraction sizes greater than 2 Gy are used, then total dose applied to the supraclavicular field must be reduced appropriately.

A suggested treatment planning protocol for this contingency may be found below. We recognise this approach is increasingly employed for an involved sentinel node, or involved node(s) at sampling, as an alternative to formal axillary clearance.

3 Technique

3.1 Position of the patient

The patients will be treated in the supine position. This position should be reproduced during simulation, acquirement of planning CT (if used) or contour and treatment. It is advised to assess the reproducibility by orthogonal laser beams.

3.2 Chest wall / Breast field.

Tangential fields will be used. Irradiation of large volumes of lung by the tangential fields should be avoided by keeping the central lung distance to less than 3 cm.

For patients with left-sided tumours, the irradiation of large volumes of heart must be avoided by keeping the distance from the posterior edge of the field to the anterior border of the heart to <1.5 cm. If these parameters cannot be met, then we recommend that either full CT planning or the use of a lead cardiac shield on the medial field should be used.

A simulator film or digital image must be taken on the medial field to verify the above parameters have been met. A minimum of one transverse outline, taken on the central axis of the length of the tangential fields should be taken.

3.3 Axilla and supra-clavicular field.

Where the clinician feels these are a necessity, an anterior supraclavicular field with an opposed posterior axillary field will be used. The upper border will cover the supraclavicular fossa and is about 3 cm above the head of the clavicle. It is suggested that a gantry angle (usually of about 15%) is used to angle the field away from the spine. The medial border is the ipsilateral edge of the vertebral bodies. The lateral border should be placed at the insertion of Teres major onto the humerus. The lower field border should be matched onto the upper border of the tangential fields. If no chest-wall fields are to be used, then the lower border of the supra-clavicular field should be at the level of the lower end of the head of the clavicle. The posterior axillary field should cover the apex of the axilla superiorly, the lower edge of anterior supraclavicular field inferiorly, and about to the lateral ends of the ribs medially. The use of a surgical clip is ideal to define the lower border of radiotherapy and upper border surgery, in the event of a level one clearance/sampling. Any shielding blocks will be indicated on a simulation film.

3.4 Supra-clavicular field.

Where the clinician feels this is a necessity, a single anterior field will be used. The infero-lateral corner should lie at the marker placed at the supra-medial limit of the axillary dissection. The upper border will cover the supraclavicular fossa and is about 3 cm above the head of the clavicle. It is suggested that a gantry angle (usually of about 15%) is used to angle the field away from the spine. The medial border is the ipsilateral edge of the vertebral bodies, the lateral border is guided by surgical clips if available, otherwise at the lateral extent of the second rib. The lower field border should be matched onto the upper border of the tangential fields. If no chest-wall fields are to be used then the lower border of the supra-clavicular field should be at the level of the lower end of the head of the clavical. Any shielding blocks will be indicated on a simulation film.

4 Dose and Fractionation

The dose distribution should be shown at least in the plane through the beam axes. The target area (PTV) in this plane should be outlined.

The tumour dose is specified at the reference point or iso-centre for the tangential fields, to the mid-plane for axillary fields and as an incident dose for the supraclavicular field.
A number of different dose/ fractionation schedules are in routine use. The following schedules are acceptable, to both the breast and nodal fields:

50 Gy / 25 daily fractions over 5 weeks
46 Gy / 23 daily fractions over 4½ weeks
45 Gy / 20 daily fractions over 4 weeks
40 Gy / 15 daily fractions over 3 weeks

or as specified by the protocol of an NCRN-approved radiotherapy protocol.

For patients having had conservative surgery, a boost to the tumour bed may be given in accordance with local protocol.

5 Treatment verification

We recognise that NHS funding constraints mean that verification films are not part of standard practice, in contrast to much of Western Europe and North America. However, where local resources do allow, it is recommended that a weekly portal imaging film (or other recording when using on-line portal imaging systems) be obtained during the course of treatment. Portal films should be compared to the simulator film. Field adjustments should be made in case of clinically important difference. This is not a requirement of the study. It should not discourage clinicians from participating.

6 Alternative methods

Some centres have developed their own specific irradiation techniques for breast, chest wall, and supraclavicular treatments. Irradiation techniques and dosages differing from those described in the protocol, e.g. electron fields for chest wall irradiation, can be allowed, provided a detailed description is given.

Alternative dose schedules are allowed if these are routinely employed by any centre, but the doses must remain constant for all arms of the trial and must be described in advance. The description of any alternative techniques and/or dose/ fractionation schedules will be reviewed by the Steering Committee prior to inclusion as a trial participant.

APPENDIX 6 ECOG Performance Status

Status	Description
**0:**	Asymptomatic, fully active and able to carry out all pre-disease performance without restriction
**1:**	Symptomatic, fully ambulatory but restricted in physically strenuous activity and able to carry out performance of a light or sedentary nature e.g. light housework, office work
**2:**	Symptomatic, ambulatory and capable of all self-care but unable to carry out any work activities. Up and about more than 50% of waking hours: in bed less than 50% of day
**3:**	Symptomatic, capable of only limited self-care, confined to bed or chair more than 50% of waking hours, but not bed-ridden
**4:**	Completely disabled. Cannot undertake any self-care. Totally bed-ridden

APPENDIX 7 New York Heart Association Functional Classification

NYHA CLASS:

Class I: Patients with cardiac disease but without resulting limitation of physical activity. Ordinary physical activity does not cause undue fatigue, palpitation, dyspnea, or anginal pain.

Class II: Patients with cardiac disease resulting in slight limitation of physical activity. They are comfortable at rest. Ordinary physical activity results in fatigue, palpitation, dyspnea, or anginal pain.

Class III: Patients with cardiac disease resulting in marked limitation of physical activity. They are comfortable at rest. Less than ordinary activity causes fatigue, palpitation, dyspnea, or anginal pain.

Class IV: Patients with cardiac disease resulting in inability to carry on any physical activity without discomfort. Symptoms of heart failure or the anginal syndrome may be present even at rest. If any physical activity is undertaken, discomfort is increased.

APPENDIX 8 Common Terminology Criteria for Adverse Events

In the present study, toxicities will be recorded according to the Common Terminology Criteria for Adverse Events (CTCAE), version 3.0. The full CTCAE document is available on the National Cancer Institute (NCI) website, at the following address:
http://ctep.cancer.gov/reporting/ctc.html

APPENDIX 9 Sample Patient Information Sheet, consent forms & GP letter

These are provided as separate documents.

APPENDIX 10 Compatibility with Other Studies

Given that this trial involves a 2x2 randomisation, there is a concern that patients might find it stressful to have to think about this study and another breast cancer systemic therapy trial at the same time. Therefore, in order to avoid the problem of "over-burdening" patients with trial choices, it is not permitted to enter patients into another systemic therapy trial within two months either side of enrolment in the TACT2 trial, nor to enter into the Quality of Life sub-study of the TACT2 trial if they have already enrolled into an ongoing QL sub-study of another trial. Similarly, if they enrol into the TACT2 Quality of Life sub-study, they should not be enrolled into another trial's QL sub-study.

The following studies are compatible with TACT2:

OPTION

This trial is only open to patients who are pre-menopausal and with ER/PgR negative tumours. It asks the question as to whether the use of a LHRH agonist with the chemotherapy would reduce the risk of premature ovarian failure and its consequent symptoms and QL effects.

REACT

The primary aim of the proposed REACT trial is to assess the disease-free survival benefit of 2 years of adjuvant therapy with celecoxib versus placebo. Possible enrolment and treatment with celecoxib/placebo will commence only on completion of adjuvant chemotherapy for primary breast cancer. The trial will be open to ER negative patients and to postmenopausal ER positive patients. ER positive patients will receive exemestane for 5 years, starting concurrently with celecoxib/placebo, exemestane and/or celecoxib in post-menopausal patients with ER positive breast cancer. The use of aromatase inhibitors in the adjuvant setting is anticipated to increase in the next few years as a result of the data from the ATAC, MA17 and IES exemestane studies, such that entering patients in a trial randomising between tamoxifen and exemestane is not thought to be detrimental to the primary questions of TACT2. Many patients could potentially be enrolled into both the REACT and TACT 2 trials, hence this trial could potentially enrol a lot of patients in TACT2, so that the TMG and IDMC will have to monitor the proportion entering both the studies and whether any selection bias is being introduced to ensure that there is no danger that one study could unduly influence the other. However, since the QL instruments used are different between the two studies, patients in the QL sub-study of TACT2 will not be eligible for REACT.

SOFT

This trial asks the question as to what is the optimum adjuvant endocrine therapy after chemotherapy in pre-menopausal patients with early breast cancer who are still menstruating post-chemotherapy. It is therefore mutually exclusive with the above proposed REACT trial. Potential women already in the TACT2 trial would be enrolled only upon completion of chemotherapy, and are randomised between 5 years' tamoxifen, 5 years ovarian ablation plus tamoxifen, and 5 years' ovarian ablation plus exemestane. Since the

majority of patients anticipated to be enrolled in TACT2 are likely to be older, the proportion of women who would meet this fundamental criteria for SOFT will be a very small group within the whole of the TACT2 population.

SUPREMO

This is an MRC trial of chest wall radiotherapy in patients with 1 – 3 nodes positive. Radiotherapy for these patients is not mandated within TACT2, and so SUPREMO is therefore compatible with TACT2.

IMPORT High

IMPORT high is a Phase III, randomised, clinical trial to test dose escalated intensity modulated radiotherapy (IMRT) after breast conservation surgery and appropriate systemic therapy in woman with higher than average risk of local tumour recurrence risk. Patients are randomised to either the control arm (40Gy to whole breast, 15 treatments over 3 weeks), Test arm 1 (36Gy to whole breast, 40Gy to partial breast, 15 treatments over 3 weeks) or Test arm 2 (40Gy to partial breast, 15 treatment over 3 weeks). The primary endpoint of IMPORT High is different to that of TACT2, being palpable induration in the irradiated breast since this is a common late effect of curative radiotherapy for early breast cancer. Those patients considered at high risk of recurrence may be eligible for entry into IMPORT High after completing chemotherapy. The invitation to take part in would occur during TACT2 follow up, patients taking part in the QL sub-study of TACT2 should not be entered into the IMPORT High QL sub-study.

ALTTO

ALLTO is a Phase III randomised, study of adjuvant lapatanib, trastuzumab, their sequence and their combination in patients with HER2 positive primary beast cancer. Patients are randomised to either (1) trastuzumab for one year, (2) lapatanib for one year, (3) trastuzumab (12 weeks) followed by a 6-week treatment free interval followed by lapatanib or (4) trastuzumab in combination with lapatanib for one year. The primary endpoint of the study is disease-free survival. HER2 positive patients who are planned for treatment with herceptin may wish to take part in ALLTO, the invitation to do so would occur during TACT2 follow up. Although the primary endpoints of both studies are the same, only a small minority of patients in either trial will be enrolled in the two studies, and both trials are randomized therefore the statistical validity of the data is maintained.

POETIC

POETIC is a phase III, randomised, clinical trial of peri-operative endocrine therapy in post-menopausal women with ER &/or PgR positive breast cancer. The primary endpoint is to determine whether four weeks perioperative treatment with an aromatase inhibitor will improve the relapse free survival interval compared with standard adjuvant treatment. Patients will be randomised in a 2:1 ratio to receive 2 weeks pre-operative and 2 weeks post-operative treatment with an aromatase inhibitor (either anastrozole or letrozole) versus no perioperative treatment. Patients will be followed up as per local practice for early breast cancer. Patients that participate in POETIC may subsequently wish to participate in TACT2,

MAPLE

MAPLE is a double-blind, short term, pre-surgical study of lapatinib in patients with primary breast cancer. Patients are randomised to receive either lapatinib treatment or placebo for 2 weeks prior to surgery and are then followed up for 30 days post-operatively. The primary endpoint is to identify molecular predictors of the anti-proliferative effects of lapatinib.

Lapatinib Presurgical (Charing Cross)

This is a Phase II, pre-surgical, study of lapatinib in patients with primary breast cancer. Patients entered into this trial will be treated for 4-6 weeks prior to surgey with lapatinib then followed up for 30 days post-surgery. The primary endpoint of this study is clinical response as assessed by RECIST after 2 weeks treatment and then prior to surgery.
